# Using Color to Control Conformation in a Chemical System Containing Multiple Tricyanofuran Photoacids

**DOI:** 10.1002/anie.202502437

**Published:** 2025-03-18

**Authors:** Matthew M. Wootten, Sofja Tshepelevitsh, Ivo Leito, Jonathan Clayden

**Affiliations:** ^1^ School of Chemistry University of Bristol Cantock's Close Bristol BS8 1TS UK; ^2^ Institute of Chemistry University of Tartu Ravila 14a Tartu 50411 Estonia

**Keywords:** Circular dichroism, Out‐of‐equilibrium systems, Photoacids, Reversible molecular logic, Wavelength selectivity

## Abstract

Color vision relies on selective, reversible isomerization by visible light of a mixture of retinyl chromophores in photoreceptor cells. Synthetic molecular mimics of this wavelength‐dependent induction of function are rare, despite the attractiveness of controlling chemical processes solely by the wavelength of incident light. Here, we report a color‐responsive chemical system that is composed of a cationic receptor complex, two competing chiral anionic ligands, and two metastable photoacids with contrasting absorption properties. Tricyanofuran photoacids are synthesized with absorption maxima of varying wavelengths across the whole visible spectrum. Protons released by the photoacids upon selective irradiation reversibly mask the more basic receptor‐bound ligand, leading to ligand exchange that can be observed as a shift in the circular dichroism (CD) spectrum of the reporter complex. A ≈90 nm separation between the absorbance maxima of the photoacids allowed each to be selectively photoisomerized in the presence of the other. The concentration of released protons, and hence the magnitude of the shift in CD response, are controlled by changing the wavelength of the incident visible light. Different output behaviors (OR/AND logic gates and wavelength detection) are programmed into the system by varying the relative proportions of the photoacids.

## Graphical abstract

In a solution of two metastable photoacids with contrasting absorption maxima, the solution pH may be control by varying the wavelength of the visible light input. This pH change governs the interaction between the Zn^2+^ cation and two competing anionic chiral ligands, detected by a change in circular dichroism (CD) spectra. Variation of the relative proportions of the photoacids allows the construction of an AND/OR logic gate and a wavelength detector.

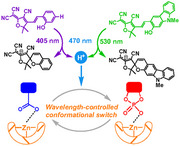

## Introduction

The development of visual systems is one of evolution's greatest marvels. For vertebrates, a complex signal transduction pathway begins with *Z*‐to‐*E* photoisomerization of an opsin‐bound retinyl chromophore within a photoreceptor cell.^[^ ^1^ ^]^ Color vision, or the ability to distinguish between different wavelengths of light, arises from multiple types of photoreceptor cells with different spectral sensitivities. For example, humans have trichromatic color vision, with three types of cone cells that are sensitive to “short” (≈420 nm), “medium” (≈530 nm), or “long” (≈560 nm) wavelengths of light.^[^ ^2^ ^]^ Incident monochromatic light stimulates each type of cone cell by different amounts, and the brain interprets these differences in photoreceptor response as color.^[^ ^3^ ^]^ Thus, a system exhibiting “color vision” may be broadly defined as one containing multiple chromophores with contrasting absorbance wavelengths, giving different outputs depending on which chromophores are selectively stimulated by electromagnetic irradiation.

Over the last few decades, diverse approaches toward color‐responsive synthetic photochromic systems have been reported. Some utilize mixtures of discrete photochromic molecules in solution,^[^ ^4^ ^]^ as a single crystal,^[^ ^5^ ^]^ bound to a polymer,^[^ ^6^, ^7^ ^]^ or doped onto a solid substrate.^[^ ^8^, ^9^, ^10^ ^]^ Others have reported single molecules possessing multiple chromophores.^[^ ^11^, ^12^, ^13^, ^14^, ^15^ ^]^ In these systems, the multicolor photochromism itself is the primary function, with applications in rewritable photopatterning,^[^ ^16^ ^]^ optical memory media,^[^ ^17^ ^]^ and optical logic gates.^[^ ^18^ ^]^ Systems with multicolor photochromism coupled to a chemical output are comparatively rare; a single example reported by Feringa et al. exploited light‐dependent solubility of one chromophore and host binding ability of another to construct a single molecule that could bind a cyclodextrin in the aqueous phase of a biphasic system only when both solubilizing and binding chromophores had been photoisomerized.^[^ ^19^ ^]^

**Figure 1:**
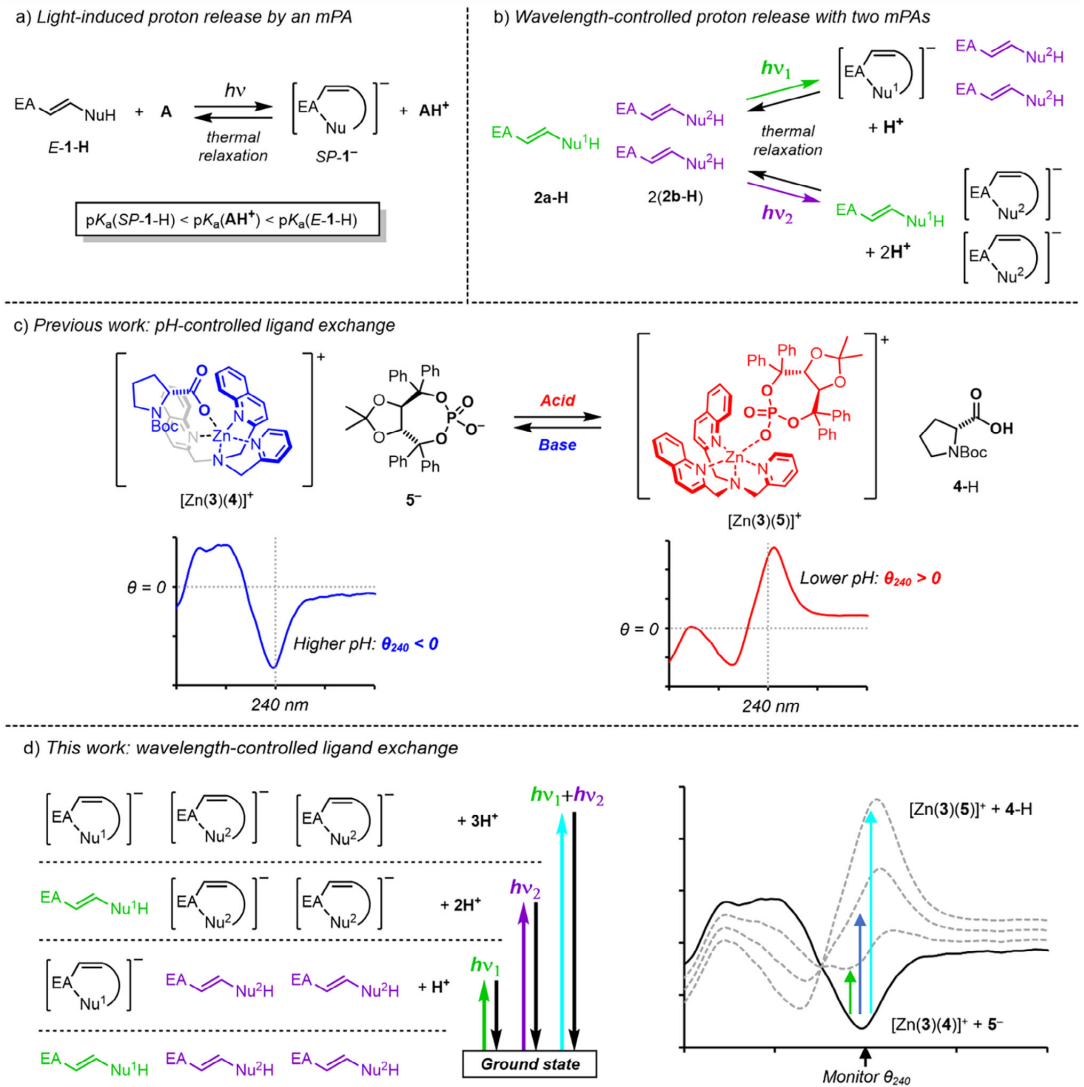
a) Light‐induced protonation of proton acceptor **A** by a general mPA **1‐H**. b) Variation in a number of released protons by irradiation wavelength. c) Protonation‐induced ligand exchange between carboxylate **3^−^** and phosphate **5^−^** at the metal centre of receptor [Zn(**3**)]^2+^, observed by CD spectroscopy. d) Varying degrees of protonation of **4^−^** according to irradiation wavelength, observed as shifts in the CD output at 240 nm.

In this paper, we describe an approach to artificial mimicry of color vision by using metastable photoacids (mPA) as chromophores.^[^ ^20^ ^]^ mPAs are composed of a protonated nucleophile (NuH) linked to an electron acceptor (EA) through a photoisomerizable carbon–carbon double bond (Figure 1a). Upon irradiation, the ground state *E*‐**1‐H** undergoes *E* to *Z* isomerization and cyclization in the presence of a proton acceptor **A** (e.g., base or solvent) to form a spirocyclic anion *SP*‐**1^−^**. The p*K*_a_ of the conjugate acid *SP*‐**1‐H** is typically several units lower than *E*‐**1‐H**, allowing acid‐promoted transformations to be activated by light. Once irradiation has ceased, the photostationary state dominated by *SP*‐**1^−^** relaxes to *E*‐**1‐H** with a half‐life ranging from seconds to hours, taking back the proton from **AH^+^** as it does so.

Despite the availability of mPAs with a variety of absorption wavelengths, reported applications are limited to single‐mPA systems, with no examples using simultaneously two or more mPAs with different absorption wavelengths.^[^ ^21^ ^]^ Nonetheless, mixtures of mPAs offer possibilities for fine‐tuned control of function. In a solution of two mPAs with contrasting λ_max_ (**2a** and **2b**) in a 1:2 ratio (Figure 1b), selective photoisomerization of **2a** or **2b** releases 1 or 2 equivalents of protons respectively, while simultaneous photoisomerization of both mPAs releases 3 equivalents of protons, assuming ideal behavior. In this way, the number of released protons can be finely controlled by tuning the wavelength of incident light.

As proof of concept, we envisaged coupling this wavelength‐selective controlled release of protons to a pH‐responsive ligand exchange. We previously showed that protonation (by physical addition of an “exogenous” acid) of a carboxylate ligand **4^−^** bound to circular dichroism (CD) active receptor complex [Zn(**3**)]^2+^ allowed a second, less basic phosphate ligand **5^−^** to displace it from the receptor binding site (Figure 1c).^[^ ^22^, ^23^ ^]^ This process was observed by monitoring the CD response at 240 nm (θ_240_), arising from the quinoline chromophores of the bis(2‐quinolylmethyl)‐(2‐pyridylmethyl)amine [BQPA] ligand **3**.^[^ ^24^ ^]^ The configuration of each ligand was chosen so that the carboxylate–receptor complex [Zn(**3**)(**4**)]^+^ gave a negative response (θ_240_ <0), while the phosphate−receptor complex [Zn(**3**)(**5**)]^+^ gave a positive response (θ_240_ >0). A greater degree of carboxylate protonation led to a greater degree of phosphate binding, and hence a greater negative to positive shift in θ_240_. We hoped that variation in the concentration of photoacidic protons through selective irradiation of multiple “endogenous” mPAs would be observable in a similar way: in other words, the system would respond to different wavelengths of light through ligand‐induced changes in the CD response of contrasting magnitude (Figure 1d).

## Results and Discussion

### Development of a Longer Wavelength Tricyanofuran Photoacid

Although zwitterionic merocyanine‐type photoacids are by far the most studied class of mPA, we instead chose to use mPAs with a tricyanofuran (TCF) acceptor group due to their greater solubility in organic solvents. However, reported TCF‐type photoacids all possess blue‐absorbing chromophores (λ_max_ ranging between 422–485 nm).^[^ ^25^, ^26^, ^27^, ^28^ ^]^ The absorption bands of the components of a mixture of these photoacids would significantly overlap, making independent photoisomerization impossible. We, therefore, needed to synthesize TCF‐type photoacids with longer absorption wavelengths, in order to identify a pair of photoacids with well‐separated absorption bands.

**Figure 2:**
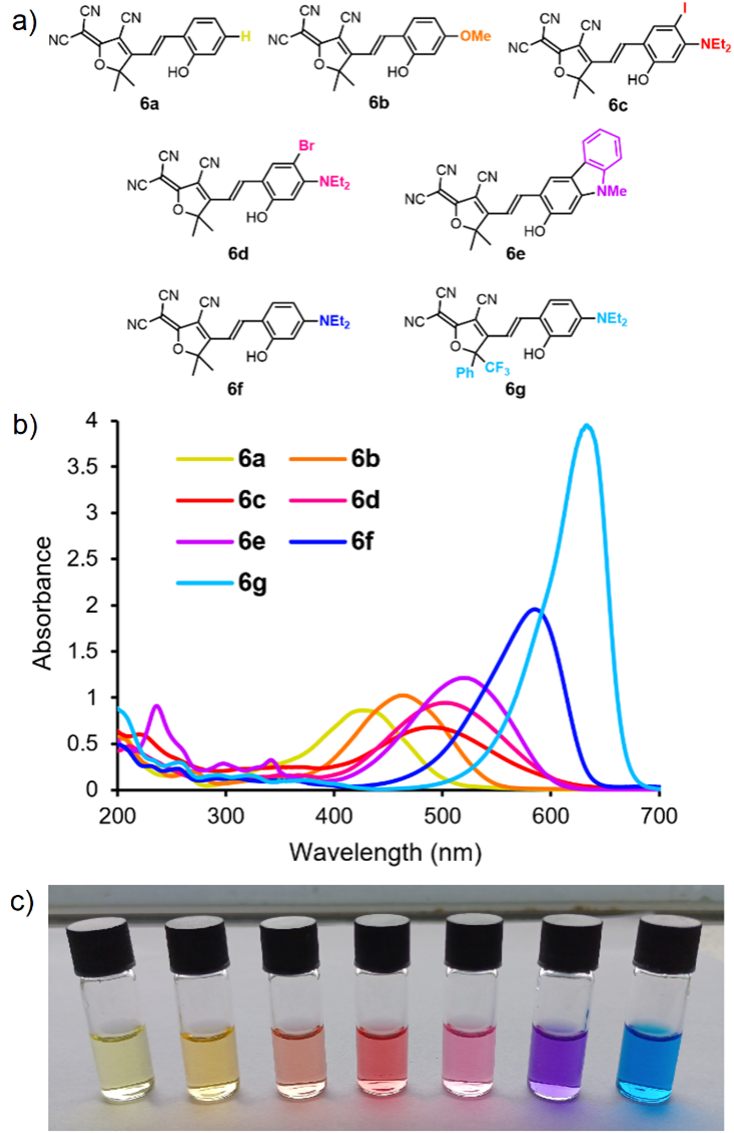
a) Structures of compounds **6a–6g**; b) UV–vis spectra and c) photograph of **6a–6g** solutions (from left to right) in acetonitrile (25 µm).

In addition to the simplest TCF‐type photoacid **6a**,^[^ ^26^ ^]^ we designed and synthesized further candidate compounds **6b**‐**6g**  (see Supporting Information for synthetic details). Compounds **6a**‐**6** **g** (Figure 2a) have increasingly electron‐rich phenolic rings, while **6g** additionally possesses a more electron‐deficient CF_3_‐bearing TCF‐acceptor group to increase the electronic “push–pull” effect. UV–vis spectra of **6a‐g** in acetonitrile displayed absorption bands covering the entire visible spectrum (Figure 2b, Table 1). **6a**, which had an unsubstituted phenol group, had the shortest λ_max_ = 425 nm. At the other end of the scale, **6g**, with λ_max_ = 634 nm, lies comfortably in the red region of the visible spectrum.

**Table 1:** Physical property data for compounds **6a**–6**g**.

Compound	λ_max_[MeCN]/nm	ε/10^6^ m ^−1^ m^−1^
**6a**	425	3.45
**6b**	464	4.09
**6c**	488	2.70
**6d**	500	3.78
**6e**	520	4.86
**6f**	586	7.83
**6g**	634	15.8

### Ligand Exchange with Individual Photoacids

**Scheme 1:**
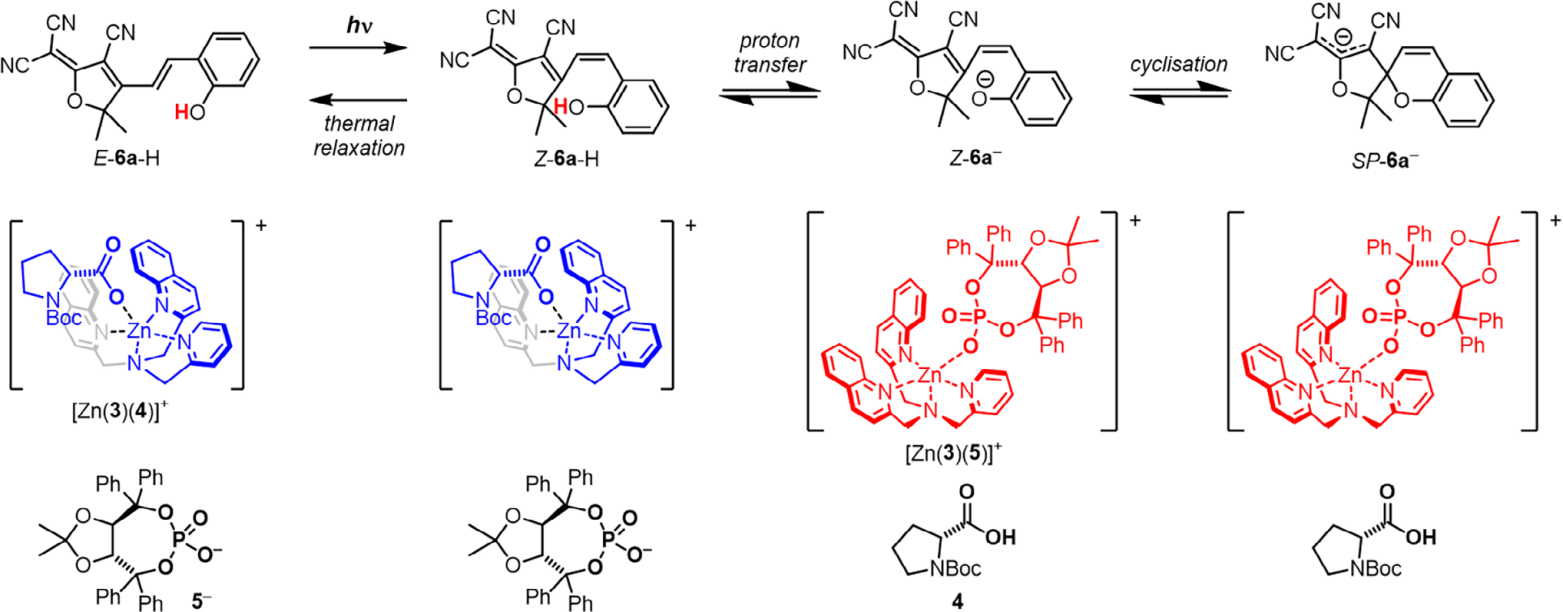
Exchange of **4** ^−^ and **5** ^−^ at [Zn(**3**)]^2+^ mediated by photoisomerisation of **6a**.

**Figure 3:**
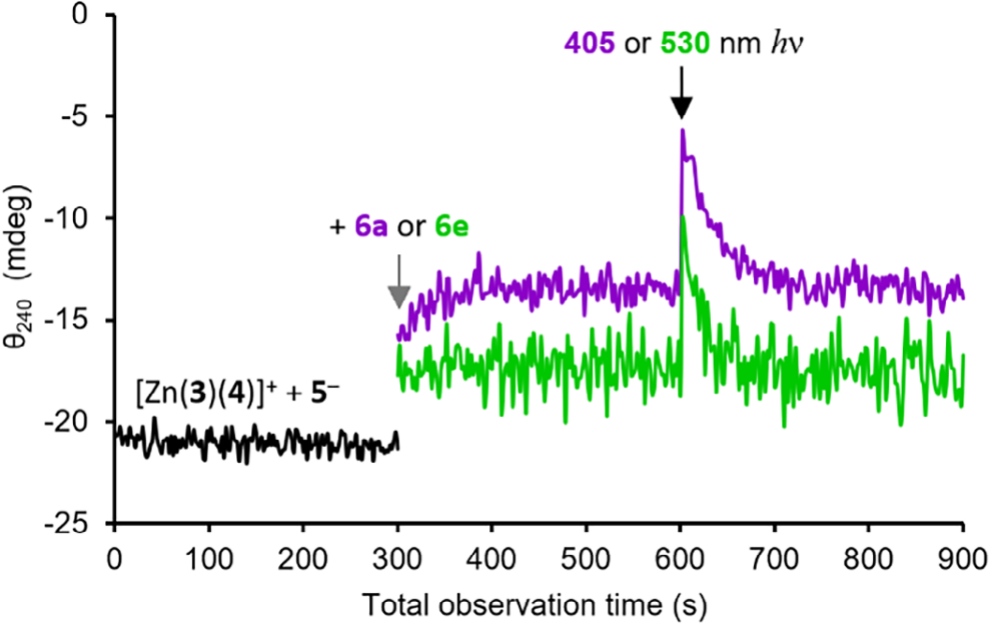
Time course CD spectroscopy measurements at 240 nm of Zn(**3**)·2ClO_4_ (25 µm), **4**‐H (1 equiv.), **5**‐H (1 equiv.), NEt_3_ (2 equiv.) in MeCN containing 5 vol% MeOH (black), followed by addition (grey arrow) of **6a** (1 equiv., purple) or **6e** (1 equiv., green). The black arrow indicates a 10 s pulse of light (405 nm for **6a**, 530 nm for **6e**).

To explore the ability of the photoacids to control ligand exchange, we introduced compound **6a** to the acid‐responsive carboxylate **4** ^−^/phosphate **5** ^−^ competitive binding equilibrium (Scheme 1). A 2000 µL solution of Zn(**3**)·2ClO_4_ (25 µm), **4**‐H (1 equiv.), **5**‐H (1 equiv.), triethylamine (2 equiv.) in acetonitrile containing 5 vol% methanol was first prepared. The CD response at 240 nm (θ_240_) of this photoacid‐free system was monitored over 5 min and an average initial response of −21.0 mdeg was observed, corresponding to the carboxylate–receptor complex [Zn(**3**)(**4**)]^+^ (Figure 3, black curve). Addition of 1 equiv. of **6a** caused θ_240_ to increase to −13.5 mdeg after equilibration (Figure 3, purple curve), suggesting that the ground state species *E*‐**6a**‐H is acidic enough to partially protonate **4** ^−^, allowing some phosphate **5** ^−^ to bind to [Zn(**3**)]^2+^. After 10 s of irradiation at 405 nm, θ_240_ increased to −5.8 mdeg (Δθ_240_ = +7.7), consistent with further protonation of **4** ^−^ by the photoisomerized photoacid *Z*‐**6a**‐H allowing more of **5** ^−^ to bind to [Zn(**3**)]^2+^. After irradiation ceased, the CD signal decayed back to the pre‐irradiation value over ca. 2 min, indicating thermal relaxation of *SP*‐**6a** ^−^ back to *E*‐**6a**‐H. Although the apparent partial protonation of **4** ^−^ even by the ground state of the photoacid was not ideal, this experiment established that the photoisomerization of **6a** could indeed alter the position of the competitive binding equilibrium.

When analogous experiments were performed with compounds **6b‐6g**, only the carbazole‐bearing derivative **6e** displayed similar photoacidic behavior. With **6e**, an average pre‐irradiation response of −17.3 mdeg increased to −10.0 mdeg (Δθ_240_ = +7.3) after 10 s of irradiation at 530 nm, again relaxing back to the pre‐irradiation equilibrium within 2 min (Figure 3, green curve). No significant light‐induced shift in θ_240_ was observed for **6b,c,d,f,g** after irradiation at an appropriate wavelength (see the Supporting Information). Evidently they do not photodegrade under these conditions, but possible reasons for the unchanged spectra in these cases are i) that the photoisomerized state of **6** is not acidic enough to protonate **4^−^**, ii) that the photoisomerized compound can protonate **4^−^** but does not cyclise, or iii) that the *E/Z* photoisomerization does not occur.

p*K*_a_(MeCN) values for the *E* and *Z* isomers of **6a** and **6e** were calculated using the COSMO‐RS method^[^ ^29^ ^]^ with empirical corrections (see the Supporting Information for further details); the results are shown in Table 2 alongside p*K*_a_ values of **4**‐H and **5**‐H. While the influence of 5 vol% MeOH on p*K*_a_ cannot be accurately predicted with this method, these p*K*_a_(MeCN) values can still serve as a guide to rationalize the photoacidic behavior of **6a** and **6e**. The added methanol is expected to systematically lower all p*K*_a_ values, but the differences in p*K*a values between *E*‐ and *Z*‐isomers is expected to remain close to or over 3 units.

**Table 2:** Calculated p*K*_a_ data (with standard uncertainty estimates) for **4**‐H, **5**‐H, **6a,** and **6e**. Cyclization of the anion was taken into account in the case of *Z*‐isomers.

Species	p*K*_a_[MeCN]
**4‐H**	22.6 ± 1.5^[^ ^22^ ^]^
*E*‐**6e‐H**	20.0 ± 0.5
*E*‐**6a‐H**	18.5 ± 0.5
**5‐H**	17.0 ± 1.5^[^ ^22^ ^]^
*Z*‐**6e‐H**	15.8 ± 0.8
*Z*‐**6a‐H**	15.0 ± 0.8

Ground state p*K*_a_ values of 18.5 and 20.0 were calculated for the *E*‐isomers of **6a** and **6e** respectively (Table 2). While these are several units lower than that of **4**‐H, the *effective* p*K*_a_ of **4**‐H is lowered due to the anion‐stabilizing effect of receptor complex [Zn(**3**)]^2+^. The degree of carboxylate protonation by the ground state of the photoacid is therefore lower than the p*K*_a_ values would suggest. The higher p*K*_a_ of *E*‐**6e**‐H accounts for the lesser degree of ground‐state protonation. In contrast, *Z*‐**6a**‐H and *Z*‐**6e**‐H had calculated p*K*_a_ values of 15.0 and 15.8, respectively, corresponding to a drop in p*K*_a_ of 3.5 and 4.2 units upon photoisomerization. (Cursory preliminary calculations show that p*K*_a_ values of *E*‐ and *Z*‐isomers of the remaining members of the set of compounds **6a‐g** typically differ by about 3–5 units.) This increase in acidity suggests that light‐induced protonation of **4** ^−^ should be feasible with both **6a** and **6e**, with **6e** being the weaker photoacid due to the higher p*K*_a_ of its *Z* isomer.

We also attempted to quantify the degree of ligand competition at the receptor binding site between **4** ^−^ and **5** ^−^ by measuring the effective association constants of **4** ^−^ and **5** ^−^ in the presence of HNEt_3_^+^ as the counterion. For each ligand, a 1:1 mixture of NEt_3_ and conjugate acid (**4**‐H or **5**‐H) was titrated into a solution of Zn(**3**)·2ClO_4_ (25 µm) in acetonitrile containing 5 vol% methanol, and the CD response at 240 nm was recorded after each addition (see Supporting Information for full details). **4**·HNEt_3_ displayed a 1:1 binding isotherm with [Zn(**3**)]^2+^ with an effective association constant of *K*_assoc_ = 7.2 × 10^5^ m ^−1^. However, **5**·HNEt_3_ displayed complex binding behavior that could not be adequately fitted to simple 1:1, 1:2, or 2:1 binding models, presumably because of other competitive binding interactions, so the degree of ligand competition was not quantified.

### Monitoring Relaxation Kinetics by UV–Vis

**Figure 4:**
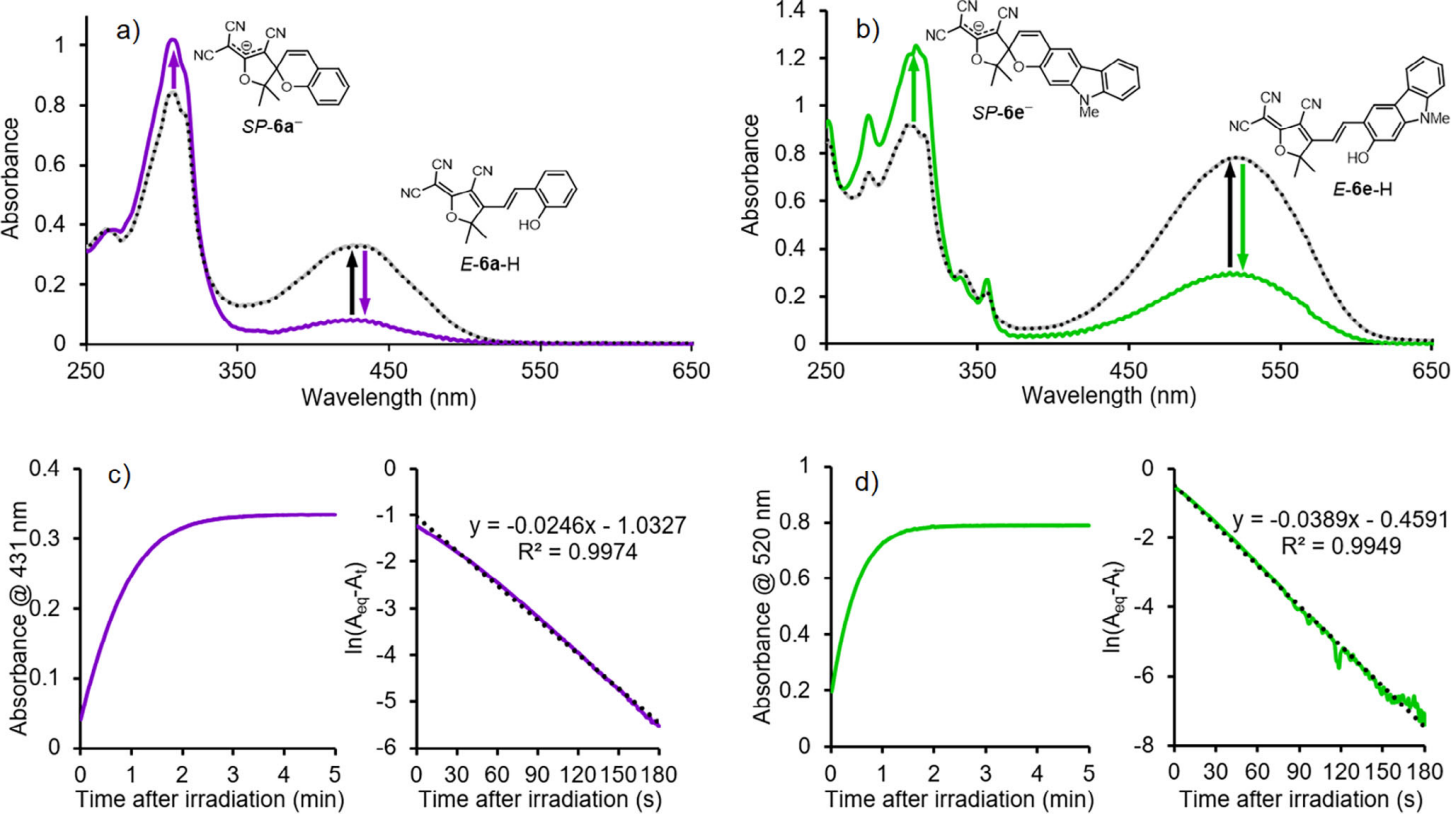
UV–vis spectra of Zn(**3**) 2ClO_4_ (25 µm), **4**‐H (1 equiv.), **5**‐H (1 equiv.), NEt_3_ (2 equiv.) and a) **6a** or b) **6e** (1 equiv.) in acetonitrile containing 5 vol% methanol prior to irradiation (grey spectra), after 10 s of irradiation with 405 or 530 nm light (purple/green spectra), and after 5 min relaxation in the dark (black dotted spectra); Time‐course absorbance measurements and 1^st^ order kinetic plots for the c) **6a** peak (431 nm) or d) **6e** peak (520 nm), following 10 s of irradiation.

The light‐induced ligand exchange systems using **6a** and **6e** were analyzed by UV–vis spectroscopy. UV–vis spectra of 2000 µL solutions of Zn(**3**)·2ClO_4_ (25 µm), **4**‐H (1 equiv.), **5**‐H (1 equiv.), triethylamine (2 equiv.), and either **6a** or **6e** (1 equiv.) in acetonitrile containing 5 vol% methanol were acquired (Figure 4a,b). Prior to irradiation, λ_max_ values of 431 nm for *E*‐**6a**‐H (Figure 4a, grey spectrum) and 520 nm for *E*‐**6e**‐H (Figure 4b, grey spectrum) were observed, confirming that the other system components and the change in solvent composition had a minimal effect on the absorption wavelength of the photoacids. A strong absorbance at ≈305 nm in both samples can be attributed to a combination of absorbance profiles of the receptor–ligand mixture and the spirocyclic conjugate base of each photoacid (*SP*‐**6a** ^−^ and *SP*‐**6e** ^−^).^[^ ^26^ ^]^ The presence of *SP*‐**6a** ^−^ and *SP*‐**6e** ^−^ prior to irradiation suggests that *E*‐**6a**‐H and *E*‐**6e**‐H can spontaneously isomerize to *SP*‐**6a** ^−^/*SP*‐**6e** ^−^ after deprotonation by **4** ^−^ in the dark. Although this reduced the magnitude of the shift in CD response upon photoisomerization, the outputs for the different wavelength inputs remained satisfactorily distinguishable.

Each sample was irradiated for 10 s at the appropriate wavelength (405 nm for **6a** and 530 nm for **6e**) and their UV–vis spectra were immediately re‐acquired (Figure 4a,b, purple and green spectra). A substantial reduction in the magnitude of the peaks at 431 and 520 nm was accompanied by an increase in the peaks at ≈305 nm, consistent with isomerization, deprotonation, and cyclization of each photoacid to form *SP*‐**6a** ^−^ and *SP*‐**6e** ^−^. After leaving the samples in the dark for 5 min, the UV–vis spectra were acquired again (Figure 4a,b, black dotted spectra). For both samples, these spectra were identical to those of the samples prior to irradiation, indicating that the relaxation of *SP*‐**6a** ^−^ and *SP*‐**6e** ^−^ back to *E‐* **6a**‐H and *E‐* **6e**‐H was complete.

The kinetics of photoacid relaxation were examined by monitoring the return of the ground‐state photoacid peaks by time‐course absorbance measurement. Both samples were irradiated again for 10 s at the same wavelength as above, and the absorbance at 431 nm (for the sample containing **6a**) or 520 nm (for the sample containing **6e**) was monitored over 5 min as the photoacids relaxed in the dark (Figure 4c,d). The data showed that the relaxation approximated to first‐order kinetics, giving rate constants of 2.46 × 10^−2^ s^−1^ for **6a** and 3.89 × 10^−2^ s^−1^ for **6e**.

### Selective Isomerisation of a Mixture of Two Photoacids

To achieve selective behavior, irradiation conditions were optimized so that each of **6a** and **6e** could be independently isomerized in the presence of the other (see Supporting Information for details). Spectral overlap between the absorption band of **6a** and the emission profile of the 530 nm LED made it necessary to use a bandpass spectral filter (FWHM = 10 nm ≈530 nm) to minimize isomerization of **6a** by green light. In contrast, the isomerization of **6e** by violet light could be minimized by simply reducing the current through the 405 nm LED from 1000 to 50 mA.

**Figure 5:**
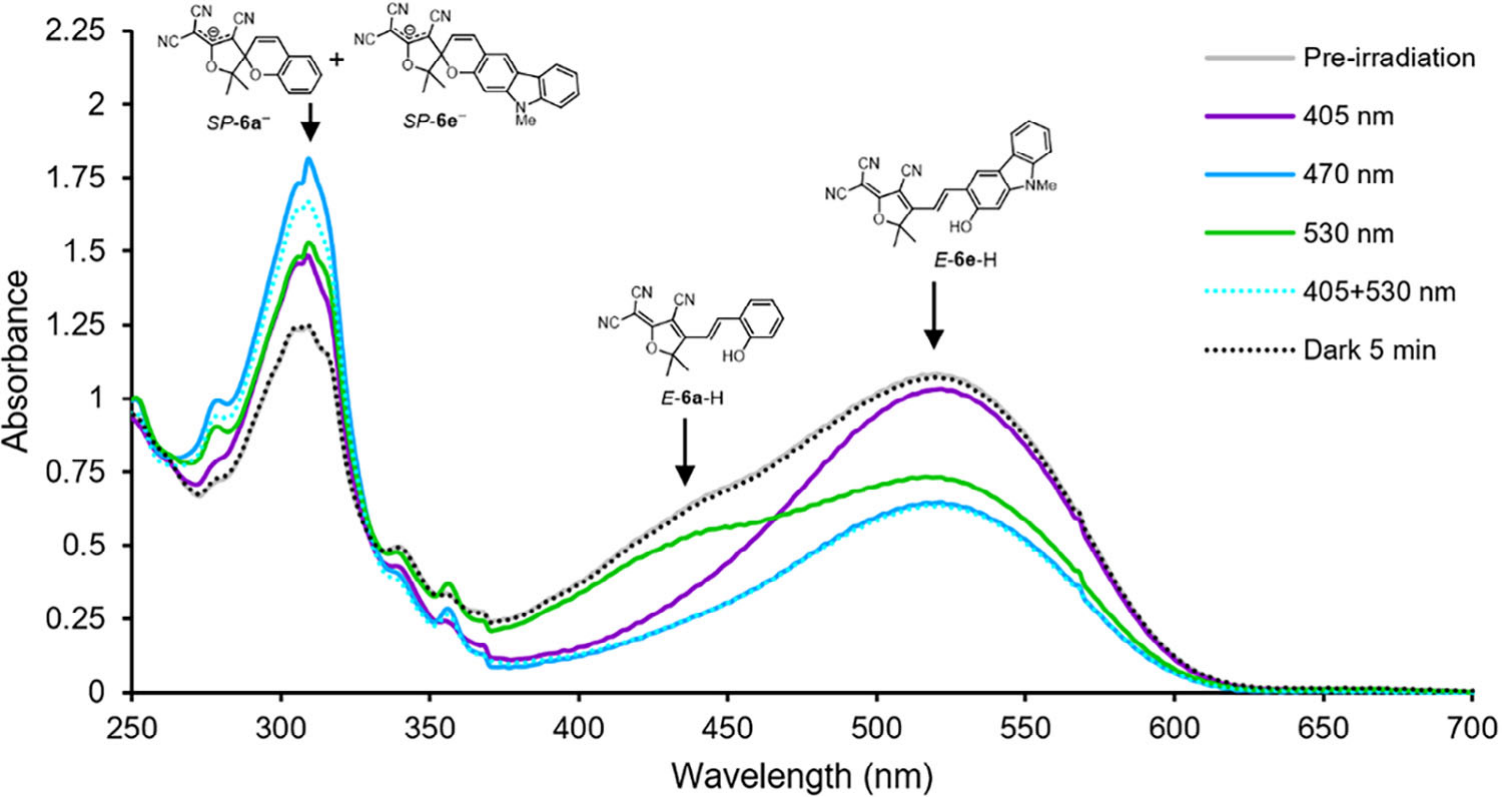
UV–vis spectra of Zn(**3**)·2ClO_4_ (25 µm), **4**‐H (1 equiv.), **5**‐H (1 equiv.), NEt_3_ (2 equiv.), **6a** (1 equiv.) and **6e** (1 equiv.) in MeCN containing 5 vol% MeOH i) Prior to irradiation (grey curve), ii) after 30 s irradiation @ 405 nm (purple curve), iii) after 30 s irradiation @ 530 nm (with bandpass filter, green curve), iv) after 30 s irradiation with @ 405 and 530 nm (dotted cyan curve), v) after 30 s irradiation @ 470 nm (blue curve), vi) after 5 mins of thermal relaxation in the dark (dotted black curve).

The independent isomerization of each of the two photoacids in a mixture was monitored by UV–vis spectroscopy. A 25 µm equimolar mixture of **6a** and **6e** in the presence of Zn(**3**)·2ClO_4_ (1 equiv.), **4**‐H (1 equiv.), **5**‐H (1 equiv.) and triethylamine (2 equiv.) in acetonitrile containing 5 vol% methanol displayed a broad absorption band between 375–600 nm, with two shoulders (at ≈430 and ≈520 nm) arising from **6a** and **6e** respectively (Figure 5, grey curve). The system was irradiated with 405 nm light for 30 s and its UV–vis absorption spectrum was immediately reacquired. This revealed a substantial reduction of the 430 nm shoulder while the 520 nm shoulder was largely unchanged (Figure 5, purple curve), consistent with isomerization primarily of **6a**. By contrast, irradiation with filtered 530 nm light for 30 s led to a loss of the 520 nm shoulder, with a smaller loss of the 430 nm shoulder, indicating primarily the isomerization of **6e**. Irradiation with both 405 nm and 530 nm gave a reduction in both shoulders of the band as **6a** and **6e** were isomerized simultaneously (Figure 5, dotted cyan curve). This could also be achieved by using a single light source with an intermediate emission maximum (470 nm), giving a post‐irradiation absorption spectrum (Figure 5, blue curve) almost identical to that after irradiation with both 405 and 530 nm light. Keeping the system in the dark for 5 min after each irradiation pulse restored the pre‐irradiation absorption band (Figure 5, black dotted curve) as **6a** and/or **6e** relaxed back to their ground states.

### Constructing an OR Gate

**Figure 6:**
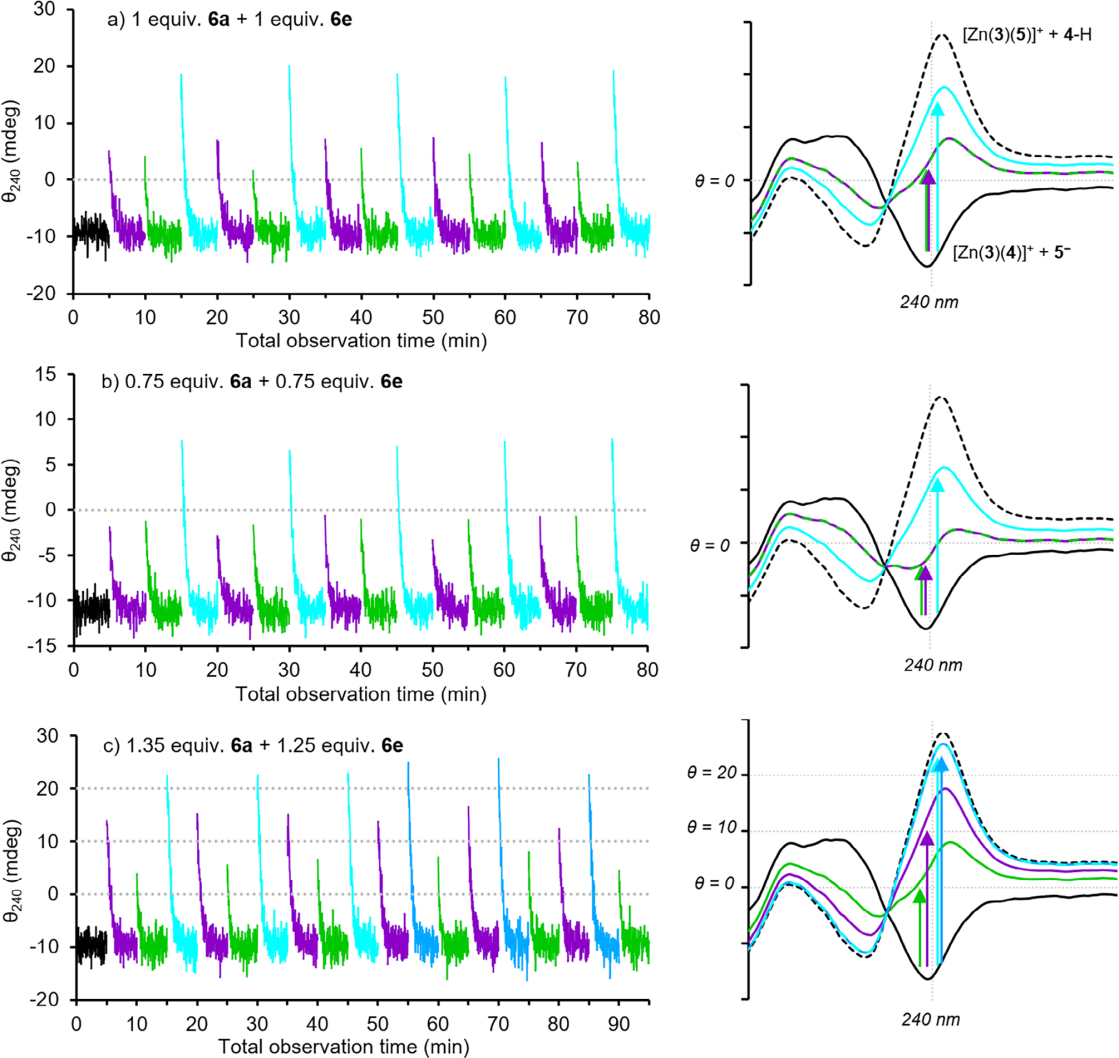
Time‐course ellipticity measurements at 240 nm of Zn(**3**)·2ClO_4_ (25 µm), **4**‐H (1 equiv.), **5**‐H (1 equiv.), NEt_3_ (2 equiv.), **6a** [a) 1 equiv., b) 0.75 equiv., c) 1.35 equiv.] and **6e** [(a) 1 equiv., b) 0.75 equiv., c) 1.25 equiv.] in MeCN containing 5 vol% MeOH for 5 min prior to irradiation (black curve), then after 30 s irradiation at 405 nm (purple curves), 530 nm (green curves), both 405 and 530 nm (cyan curves) or 470 nm (blue curves). The graphs on the right are qualitative representations of how the CD spectrum of the system might appear after irradiation, compared to the spectra of [Zn(**3**)(**4**)]^+^ (solid black curve) and [Zn(**3**)(**5**)]^+^ (dotted black curve).

The same system was studied by CD spectroscopy. First, a 2000 µL solution of Zn(**3**)·2ClO_4_ (*c* = 25 µm), **4**‐H (1 eq.), **5**‐H (1 equiv.), triethylamine (2 equiv.), **6a** (1 equiv.) and **6e** (1 equiv.) in acetonitrile with 5 vol% methanol was prepared. The system was allowed to equilibrate in the dark for 5 min and an average ground state CD response of θ_240_ = −9.2 mdeg was observed (Figure 6a). The system was then irradiated with each wavelength individually, then the CD response was monitored for 5 min while the sample was kept in the dark. After irradiation with 405 nm light for 30 s, θ_240_ swung to a positive value of +5.1 mdeg (Δθ_240_ = +14.3), relaxing back to the equilibrium value over 3 min. Irradiation with 530 nm light for 30 s gave a CD response of θ_240_ = +4.1 mdeg (Δθ_240_ = +13.3), which again relaxed back to the equilibrium value over 3 min.

Simultaneous irradiation with both 405 and 530 nm light gave a significantly increased positive response of +18.4 mdeg (Δθ_240_ = +27.6), consistent with a greater degree of protonation of the carboxylate ligand when both photoacids are irradiated instead of just one. The system was then cycled through pulses of 405 nm, 530 nm, and both 405 and 530 nm light for a further 4 full cycles. Comparable responses were observed for each wavelength (or wavelength‐combination) pulse, and the average resting state response remained largely unchanged throughout the experiment, demonstrating the system's resilience toward multiple irradiation–relaxation cycles. However, the response with 530 nm irradiation was consistently lower in magnitude than with 405 nm irradiation due to the lower photoacidity of **6e** compared to **6a**.

If the *sign* of the CD response is taken as a Boolean output instead (negative = 0 or *off*, positive = 1 or *on*), then the behavior of the system is equivalent to that of a reversible OR logic gate, i.e., the system responds with an *on* output when either or both inputs are active but relaxes to an *off* output once the inputs are removed. Conventional solution‐state^[^ ^30^, ^31^, ^32^ ^]^ molecular logic gates, first reported by de Silva in 1993,^[^ ^33^ ^]^ typically require chemical inputs such as the addition of protons or metal cations.^[^ ^34^, ^35^ ^]^ These often suffer from the lack of reversibility – once the input species are bound to the molecular logic gate, it is entropically unfavorable to separate them. In our approach, however, a reversible chemical response (i.e., ligand exchange) is coupled to out‐of‐equilibrium isomerization of the photoacids. Once the irradiation input is removed, the system can relax back to its equilibrium *off* state and repeatedly perform logic operations.

### Constructing an AND Gate

Next, the concentration of both **6a** and **6e** was reduced by 25% while leaving the concentrations of the other components unchanged. A slightly lower average ground state response of θ_240_ = −10.9 mdeg was observed, because of reduced protonation of **4** ^−^ by the ground state photoacids (Figure 6b, black curve). A response of −1.9 mdeg (Δθ_240_ = +9.0) was recorded after a 405 nm light pulse, relaxing back to the pre‐irradiation resting state over 3 min in the absence of light (Figure 6b, purple curves). Similarly, a response of −1.2 mdeg (Δθ_240_ = +8.3) was recorded after a 530 nm light pulse, relaxing back to −11 mdeg over 2 min (Figure 6b, green curves). Irradiation with both 405 and 530 nm light sources instead gave a positive initial response of +7.7 mdeg (Δθ_240_ = +18.6), again relaxing back to −11 mdeg over 3 min (Figure 6b, cyan curves).

In contrast to the previous system, the reduced number of released protons caused the response to remaining negative (i.e., *off*) when the system was irradiated with either wavelength individually due to insufficient protonation of the carboxylate ligand, only becoming positive (i.e., *on*) when both **6a** and **6e** were isomerized. This system therefore constitutes a reversible AND gate, consistently displaying this output pattern over 5 cycles of irradiation with 405 nm, 530 nm then both 405 and 530 nm light.

### Constructing a Wavelength Detector

The responses described above, resulting from irradiation with either 405 or 530 nm light, were very similar, so the behavior of the above systems cannot be described as mimicking color vision. By changing the stoichiometry of the photoacids, we were able to develop a system that could distinguish between the two monochromatic inputs.

In the OR gate system, there is a greater degree of carboxylate protonation with **6a** than with **6e**. This difference was amplified in a system with a higher concentration of **6a** than **6e**. A 2000 µL solution of Zn(**3**)·2ClO_4_ (*c* = 25 µm), **4**‐H (1 eq.), **5**‐H (1 equiv.), triethylamine (2 equiv.), **6a** (1.35 equiv.) and **6e** (1.25 equiv.) in acetonitrile with 5 vol% methanol was prepared. After equilibration in the dark, a pre‐irradiation average response of −9.5 mdeg was recorded (Figure 6c, black curve). Irradiation with separate pulses of 405 and 530 nm light gave initial responses of +13.9 mdeg (Δθ_240_ = +23.4) and +3.4 mdeg (Δθ_240_ = +12.9) respectively (Figure 6c, purple and green curves), while simultaneous irradiation with both wavelengths gave a significantly higher initial response of +22.5 mdeg (Δθ_240_ = +32.0) (Figure 6c, cyan curves). As before, the system relaxed back to the pre‐irradiation state within 5 min in the absence of light.

By choosing convenient cut‐off points at θ_240_ = 0, +10, and + 20 mdeg, the system could reliably distinguish between inputs of either or both wavelengths; a response of θ_240_ <0 mdeg corresponds to the *off* state, 0 ≤ θ_240_ <10 mdeg corresponds to a 530 nm input, 10 ≤ θ_240_ <20 mdeg corresponds to a 405 nm input, while θ_240_ ≥20 mdeg corresponds to simultaneous 405 and 530 nm inputs. Irradiation with monochromatic 470 nm light gave similar responses to simultaneous irradiation with 405 and 530 nm light (Figure 6c, blue curves). This property allowed the system to behave as a color detector, giving contrasting responses in response to violet, blue, and green light inputs.

It is important to note that although comparable wavelength‐dependent behavior could be achieved with a single photoacid, a second photoacid that can be independently photoisomerized provides an additional variable (the relative proportions of **6a** and **6e**) that may be systematically controlled to tune the output CD response for each wavelength input. This proved to be helpful for optimizing the wavelength detector system to give well‐separated output values. Any output between the minimum and maximum values could of course be trivially engineered by tuning the irradiation intensity. However, such a system responds to photon flux as a *continuous* variable, whereas logic gates require, by definition, a discrete binary input.

## Conclusion

In conclusion, we have developed a wavelength‐responsive chemical system that reversibly translates the wavelength of irradiation with visible light input into an orthogonal CD response by coupling the photoisomerization of two different photoacids with protonation‐induced ligand exchange at a CD reporter complex.

Converting the sign of the CD response into a Boolean output led to emergent AND gate and OR gate behavior depending on the concentration of the photoacids relative to the other components, while wavelength‐detecting behavior was achieved by having unequal photoacid stoichiometries. Additionally, the inverse NAND and NOR gates could be theoretically accessed by using the opposite enantiomers of the carboxylate and phosphate ligands. The use of light as a non‐chemical input alongside thermally reversible photochromism allows input–output cycles to be performed repeatedly, overcoming the problem of irreversibility seen with molecular logic gates with chemical inputs.

The selective irradiation of chromophores with contrasting absorption profiles is reminiscent of the action of opsins in biological color vision. In our case, the use of two chromophores leads to rudimentary dichromatic vision, but the addition of a red‐responsive chromophore suggests the possibility of a full RGB color system, much like in human trichromatic vision. By considering the isomerization state of each photoacid as one bit of information, we envision that up to eight states (2^3^) of varying proton concentration could be programmed in this way.

## Supporting Information

Synthetic procedures, characterization of novel compounds, CD and UV–vis spectroscopy methodology, and p*K*_a_ calculation methodology may be found in the Supporting Information.

## Conflict of Interests

The authors declare no conflict of interest.

## Supporting information



Supporting Information

## Data Availability

The data that support the findings of this study are available in the supplementary material of this article.

## References

[1] T. Ebrey , Y. Koutalos , Prog. Retinal Eye Res. 2001, 20, 49–94. 10.1016/s1350-9462(00)00014-8 11070368

[2] J. K. Bowmaker , H. J. Dartnall , J. Physiol. 1980, 298, 501–511. 7359434 10.1113/jphysiol.1980.sp013097 PMC1279132

[3] S. G. Solomon , P. Lennie , Nat. Rev. Neurosci. 2007, 8, 276–286. 17375040 10.1038/nrn2094

[4] G. M. Tsivgoulis , J. M. Lehn , Adv. Mater. 1997, 9, 627–630.

[5] M. Morimoto , S. Kobatake , M. Irie , J. Am. Chem. Soc. 2003, 125, 11080–11087. 12952490 10.1021/ja035277o

[6] H. Nishi , T. Namari , S. Kobatake , J. Mater. Chem. 2011, 21, 17249–17258.

[7] S. Ulrich , J. R. Hemmer , Z. A. Page , N. D. Dolinski , O. Rifaie‐Graham , N. Bruns , C. J. Hawker , L. F. Boesel , J. Read De Alaniz , ACS Macro Lett. 2017, 6, 738–742. 35650854 10.1021/acsmacrolett.7b00350

[8] I. Kawashima , H. Takahashi , S. Hirano , R. Matsushima , J. Soc. Inf. Disp. 2004, 12, 81–85.

[9] T. Qin , J. Han , Y. Geng , L. Ju , L. Sheng , S. X.‐A. Zhang , Chem. ‐ Eur. J. 2018, 24, 12539–12545. 29935037 10.1002/chem.201801692

[10] S. Zhou , S. Guo , W. Liu , R. Ding , H. Sun , J. Chen , Z. Qian , H. Feng , J. Mater. Chem. C 2021, 9, 8249–8257.

[11] K. Higashiguchi , K. Matsuda , N. Tanifuji , M. Irie , J. Am. Chem. Soc. 2005, 127, 8922–8923. 15969548 10.1021/ja051467i

[12] M. Frigoli , G. H. Mehl , Angew. Chem., Int. Ed. 2005, 44, 5048–5052. 10.1002/anie.200462575 16035008

[13] A. Fihey , A. Perrier , W. R. Browne , D. Jacquemin , Chem. Soc. Rev. 2015, 44, 3719–3759. 25921433 10.1039/c5cs00137d

[14] F. Zhao , L. Grubert , S. Hecht , D. Bléger , Chem. Commun. 2017, 53, 3323–3326. 10.1039/c7cc00504k 28210737

[15] K. Mutoh , K. Yamamoto , J. Abe , Photochem. Photobiol. Sci. 2022, 21, 1445–1458. 35527290 10.1007/s43630-022-00234-y

[16] Q. Zhu , J. Zuo , X. Ping , Y. Zhu , X. Cai , Z. Xiong , Z. Qian , H. Feng , J. Mater. Chem. C 2022, 10, 8674–8683.

[17] K. Uchida , M. Saito , A. Murakami , T. Kobayashi , S. Nakamura , M. Irie , Chem. ‐ Eur. J. 2005, 11, 534–542. 15565721 10.1002/chem.200400575

[18] S. D. Straight , P. A. Liddell , Y. Terazono , T. A. Moore , A. L. Moore , D. Gust , Adv. Funct. Mater. 2007, 17, 777–785.

[19] M. M. Lerch , M. J. Hansen , W. A. Velema , W. Szymanski , B. L. Feringa , Nat. Commun. 2016, 7, 12054. 27401266 10.1038/ncomms12054 PMC4945879

[20] Y. Liao , Acc. Chem. Res. 2017, 50, 1956–1964. 28692282 10.1021/acs.accounts.7b00190

[21] Y. Liao , Phys. Chem. Chem. Phys. 2022, 24, 4116–4124. 35129187 10.1039/d1cp05627a

[22] M. M. Wootten , B. A. F. Le Bailly , S. Tshepelevitsh , I. Leito , J. Clayden , Chem. Sci. 2022, 13, 2258–2269. 35310487 10.1039/d1sc06812a PMC8864710

[23] M. M. Wootten , S. Tshepelevitsh , I. Leito , J. Clayden , Chem. ‐ Eur. J. 2022, 28, e202202247. 35880579 10.1002/chem.202202247 PMC9804598

[24] L. A. Joyce , M. S. Maynor , J. M. Dragna , G. M. Da Cruz , V. M. Lynch , J. W. Canary , E. V. Anslyn , J. Am. Chem. Soc. 2011, 133, 13746–13752. 21780788 10.1021/ja205775g PMC3179184

[25] V. K. Johns , P. Peng , J. DeJesus , Z. Wang , Y. Liao , Chem. ‐ Eur. J. 2014, 20, 689–692. 24318615 10.1002/chem.201304226

[26] C. Yang , T. Khalil , Y. Liao , RSC Adv. 2016, 6, 85420–85426.

[27] M. Y. Belikov , M. Y. Ievlev , S. V. Fedoseev , O. V. Ershov , New J. Chem. 2019, 43, 8414–8417.

[28] M. Y. Belikov , M. Y. Ievlev , I. N. Bardasov , New J. Chem. 2021, 45, 10287–10295.

[29] F. Eckert , I. Leito , I. Kaljurand , A. Kütt , A. Klamt , M. Diedenhofen , J. Comput. Chem. 2009, 30, 799–810. 18727157 10.1002/jcc.21103

[30] Solid state optical logic gates have been previously reported T. Zhang , C. Zhang , G. Fu , Y.‐D. Li , L. Gu , G. Zhang , Q. W. Song , B. Parsons , R. R. Birge , Opt. Eng. 2000, 39, 527–534.

[31] J. Matsui , M. Mitsuishi , A. Aoki , T. Miyashita , J. Am. Chem. Soc. 2004, 126, 3708–3709. 15038712 10.1021/ja039871+

[32] K. Szaciłowski , W. Macyk , G. Stochel , J. Am. Chem. Soc. 2006, 128, 4550–4551. 16594673 10.1021/ja060694x

[33] P. A. De Silva , N. H. Q. Gunaratne , C. P. McCoy , Nature 1993, 364, 42–44.

[34] S. Erbas‐Cakmak , S. Kolemen , A. C. Sedgwick , T. Gunnlaugsson , T. D. James , J. Yoon , E. U. Akkaya , Chem. Soc. Rev. 2018, 47, 2228–2248. 29493684 10.1039/c7cs00491e

[35] L. Liu , P. Liu , L. Ga , J. Ai , ACS Omega 2021, 6, 30189–30204. 34805654 10.1021/acsomega.1c02912 PMC8600522

