# Supplementary material for: Using Color to Control Conformation in a Chemical System Containing Multiple Tricyanofuran Photoacids
Source: Angew Chem Int Ed Engl. 2025 Mar 18;64(21):e202502437. doi: 10.1002/anie.202502437 (PMC12087846; doi:10.1002/anie.202502437)
Supplement: Supplementary file 1 — Supporting Information [file ANIE-64-e202502437-s001.pdf]

Table of Contents

S1 General information & Instrumentation 2

S2 Synthetic details 4

S3 Stock solution preparation 16

S4 Additional CD spectroscopy methodology 16

S4.1 Light-induced ligand exchange with **6a-g** (Fig. 3) 16

S4.2 Optimisation of irradiation conditions 17

S4.3 Association constant measurements 19

S5 Calculation of pK_a values 20

S6 ^{1}H and ^{13}C NMR spectra of photoacids and intermediates 22

S7 References 39

S1 General information & Instrumentation

Air and/or moisture sensitive reactions were performed under a nitrogen atmosphere with glassware that was oven-dried or dried by heat gun, using standard anhydrous techniques. Air and/or moisture sensitive liquids and solutions were transferred via syringe through rubber septa.

All reagents and solvents were purchased from commercial suppliers and used as received, unless otherwise stated. Triethylamine was stored over KOH pellets. Anhydrous dichloromethane (DCM) and tetrahydrofuran (THF) were dried using an Anhydrous Engineering Grubbs-type solvent system. Anhydrous acetonitrile (MeCN) was purchased from Acros Organics and had a water content of 4.1 ppm, measured by Karl Fischer titration (average of 3 measurements). Anhydrous methanol (MeOH) had a water content of 42.6 ppm, measured by Karl Fischer titration (average of 3 measurements). P.E. refers to fractions of petroleum ether with boiling points in the range 40–60 °C.

Flash column chromatography was performed on silica gel (VWR 40-63 µm, 230-300 mesh). Analytical thin-layer chromatography (TLC) was performed on aluminium-backed silica (Merck 60 Å F_{254}), visualising using a UV lamp (254 nm), potassium permanganate or phosphomolybdic acid stain.

Receptor complex **3**·$2ClO_4$ and phosphate ligand **5**-H were prepared according to our previous work.[1]

^{1}H and ^{13}C NMR spectra were acquired on Bruker (400 or 500 MHz) or Jeol ECS (400 MHz) spectrometers. Chemical shifts (δ) are reported in parts per million (ppm) and coupling constants (*J*) are reported in Hertz (Hz) to the nearest 0.1 Hz. ^{1}H-NMR spectra are referenced to the residual solvent peak ($CDCl_3$ 7.26, CD_3OD 3.31) and ^{13}C-NMR were referenced to the ^{13}C resonance of the solvent ($CDCl_3$ 77.16, CD_3OD 49.00). Multiplicities are reported as singlet (s), doublet (d), triplet (t), quartet (q), multiplet (m), broad (br) or combinations thereof.

Infrared spectra were recorded on a Perkin Elmer Spectrum Two spectrometer. Only major absorption maxima (ν_{max}) above 1000 cm^{-1} are reported and quoted in wavenumbers (cm^{-1}).

High-resolution ESI spectra were acquired on Bruker micrOTOF II or Thermo Scientific Orbitrap Elite spectrometers. High-resolution Nanospray spectra were acquired on a Waters Synapt G2S spectrometer with Advion Nanomate installed.

UV-vis spectra were acquired on an Agilent Cary 300 UV-vis spectrometer at the stated concentration and ambient temperature in a 10 mm path length quartz cuvette.

CD spectra were acquired on a JASCO J-815 CD spectrometer at 25 °C and at the stated concentration using a 10 mm path length quartz cuvette.

Visible light irradiation was performed at ambient temperature using a Thorlabs DC4100 4-channel LED driver at the stated current with Thorlabs M405L4, M470L5, M530L4, MINTL5 (nominal wavelength 554 nm) and M625L4 mounted LEDs with attached collimators. For systems containing both **6a** and **6e**, a FBH530-10 bandpass spectral filter was attached to the 530 nm LED (M530L4) via an SM1QU filter holder.

Throughout this study, sample irradiation was performed in a cardboard box (width approx. 11 cm) with two apertures on opposite sides in which mounted LEDs could be inserted. This setup allowed a 10 mm cuvette placed in the centre of the box to be freely irradiated with either or both LEDs at a distance of 5 cm. Figure **S1** shows a sample being irradiated with 405 nm, 530 nm and both 405 and 530 nm LEDs.

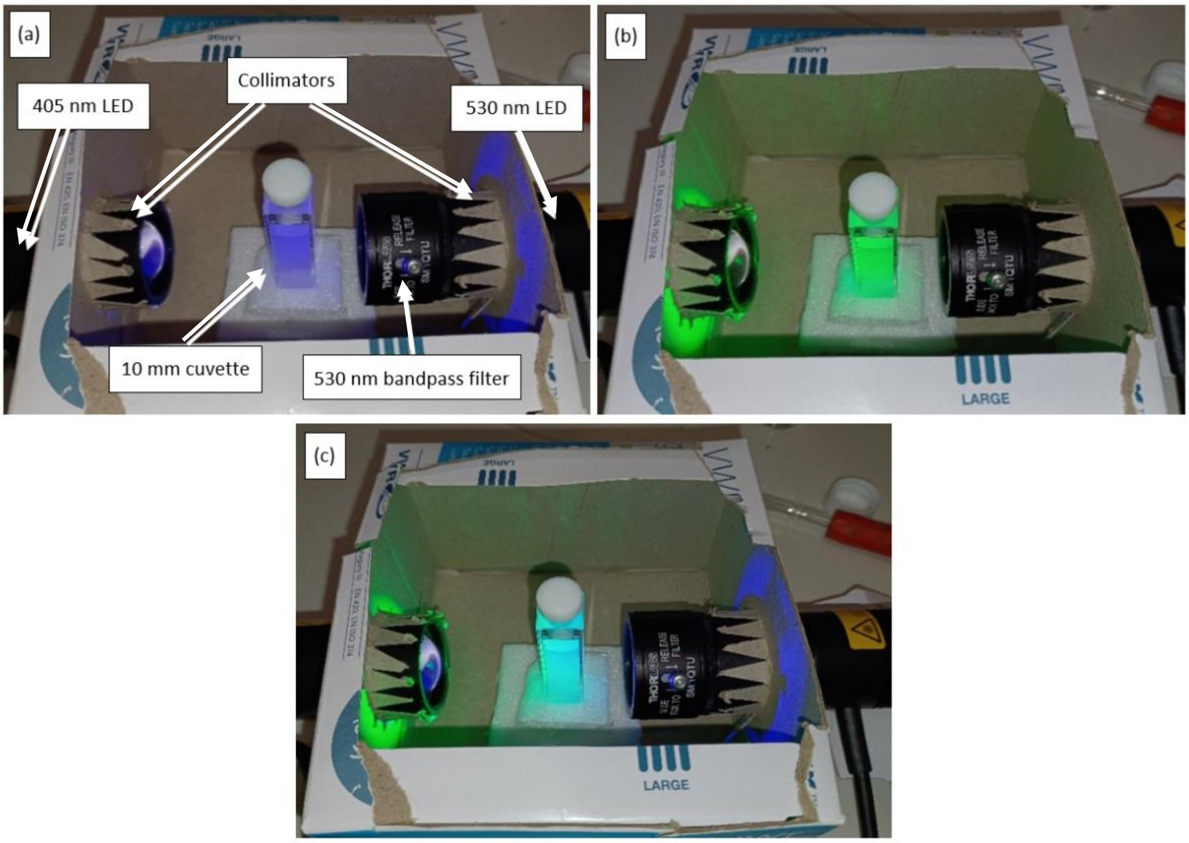

Figure S1: Photographs of the irradiation setup used in this study, showing a 10 mm cuvette (containing water mixed with a few drops of milk) under irradiation with (a) 405 nm, (b) 530 nm or (c) both 405 nm and 530 nm LEDs.

S2 Synthetic details

General procedure for photoacid synthesis by condensation of TCF acceptors with aldehydes

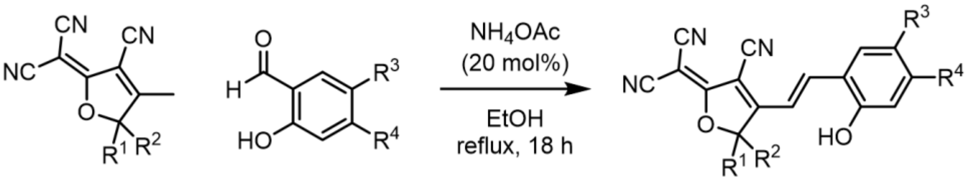

A mixture of TCF acceptor (1 equiv.), aldehyde (1.2 equiv.) and NH_4OAc (0.2 equiv.) was dissolved in EtOH (10 mL mmol^{-1}) and heated under reflux for 18 hours. Except where specified, the photoacid product was isolated by cooling the reaction mixture to 0 °C and collecting the precipitated product by filtration. The filter cake was washed with ice-cold EtOH followed by ice-cold Et_2O, then dried under reduced pressure to give the product. In some cases, azeotropic removal of residual EtOH with $CHCl_3$ was required.

Synthesis of 6a

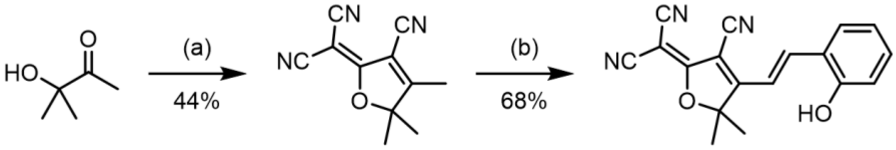

7 6a

Reagents and conditions: (a) $CH_2(CN)_2$, NaOEt, EtOH, 90 °C (µw), 15 min; (b) Salicylaldehyde, NH_4OAc, EtOH, 78 °C, 18 h.

2-(3-Cyano-4,5,5-trimethylfuran-2(5H)-ylidene)malononitrile (7)

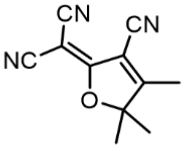

Prepared according to a modified literature procedure.[2] A solution of sodium ethoxide was prepared by dissolving sodium metal (22 mg, 0.95 mmol) in ethanol (1 mL) in a microwave vial. Freshly recrystallised malononitrile[3] (1.26 g, 19.0 mmol), 3-hydroxy-3-methyl-2-butanone (1.0 mL, 9.5 mmol) and a Teflon-coated stirrer bar were added, then the microwave vial was sealed. The mixture was heated at 90 °C (microwave irradiation) for 15 minutes, then cooled and poured into aq. HCl (1 M). The resulting precipitate was collected, washed with cold ethanol, then dried under vacuum to give the title compound (840 mg, 44%) as a bright yellow solid.

^{1}H NMR (400 MHz, $CDCl_3$): δ_H 2.36 (s, 3H, CH_3), 1.63 (s, 6H, $C(CH_3)_2$)

^{13}C NMR (101 MHz, DMSO-d_6): δ_C 185.7, 177.3, 112.2, 111.5, 110.0, 103.6, 101.3, 54.8, 23.2 ($C(\mathbf{C}H_3)_2$), 14.2 (CH_3)

Spectroscopic data are consistent with those previously reported.[4]

(*E*)-2-(3-Cyano-4-(2-hydroxystyryl)-5,5-dimethylfuran-2(5*H*)-ylidene)malononitrile (6a)

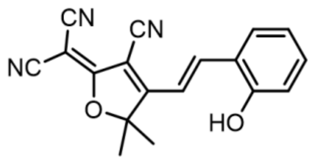

Prepared according to the general procedure using **7** (50 mg, 0.25 mmol), salicylaldehyde (32 µL, 0.31 mmol), and NH_4Cl (4 mg, 0.05 mmol). The title compound (52 mg, 68%) was obtained as a yellow solid.

^{1}H NMR (400 MHz, DMSO-*d*$_6$): δ_H 10.86 (s, 1H, OH), 8.17 (d, *J* = 16.5, 1H, HC=C**H**), 7.84 (dd, *J* = 7.9, 1.6, 1H, ArH), 7.42 (d, *J* = 16.5, 1H, **H**C=CH), 7.37 (ddd, *J* = 8.5, 7.3, 1.7, 1H, ArH), 6.97–6.90 (m, 2H, ArH), 1.77 (s, 6H, $C(CH_3)_2$)

^{13}C NMR (101 MHz, DMSO-*d*$_6$): δ_C 177.4, 176.3, 158.5, 144.0 (HC=**C**H), 134.1 (ArH), 130.6 (ArH), 121.3, 119.9 (ArH), 116.6 (ArH), 115.2 (H**C**=CH), 112.9, 112.1, 111.3, 99.2, 97.5, 53.8, 25.2 (C(**C**H$_3$)$_2$)

Spectroscopic data are consistent with those previously reported.[5]

Synthesis of 6b

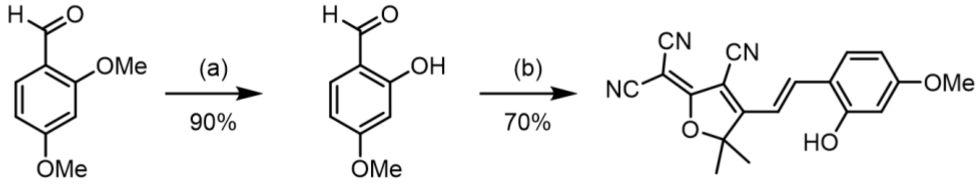

8 6b

Reagents and conditions: (a) BBr_3, DCM, rt, 18 h; (b) **7**, NH_4OAc, EtOH, 78 °C, 18 h.

2-Hydroxy-4-methoxybenzaldehyde (8)

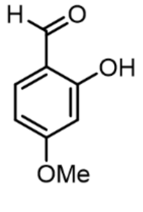

Boron tribromide (1.05 mL, 1 M in DCM, 1.05 mmol) was added dropwise to a solution of 2,4-dimethoxybenzaldehyde (166 mg, 1.0 mmol) in dichloromethane (4 mL). The mixture was stirred at room temperature for 18 hours, then quenched by the addition of ice (5 g). The mixture was extracted with EtOAc with a small amount of MeOH to aid solubility, and the combined organic extracts were washed with brine, then dried (Na_2SO_4), filtered and evaporated under reduced

pressure to give the title compound (137 mg, 90%) as a yellow oil, which crystallised on standing.

^{1}H NMR (400 MHz, DMSO-d_6): δ_H 11.06 (br s, 1H, OH), 9.98 (s, 1H, CHO), 7.61 (d, J = 8.7, 1H, ArH), 6.56 (dd, J = 8.7, 2.4, 1H, ArH), 6.47 (d, J = 2.4, 1H, ArH), 3.80 (s, 3H, CH_3)

^{13}C NMR (101 MHz, DMSO-d_6): δ_C 191.4 (CHO), 166.1 (Ar), 163.2 (Ar), 132.5 (Ar), 116.3 (Ar), 107.6 (Ar), 100.9 (Ar), 55.8 (CH_3)

Spectroscopic data are consistent with those previously reported.[6]

(*E*)-2-(3-Cyano-4-(2-hydroxy-4-methoxystyryl)-5,5-dimethylfuran-2(5H)-ylidene) malononitrile (6b)

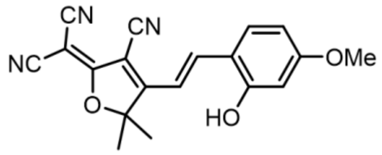

Prepared according to the general procedure, using **7** (50 mg, 0.25 mmol), **8** (46 mg, 0.3 mmol) and NH_4Cl (4 mg, 0.05 mmol). The title compound (58 mg, 70%) was obtained as an orange solid.

^{1}H NMR (400 MHz, DMSO-d_6): δ_H 11.07 (s, 1H, OH), 8.15 (d, J = 16.3, 1H, HC=C**H**), 7.82 (d, J = 8.8, 1H, ArH), 7.27 (d, J = 16.3, 1H, **H**C=CH), 6.57 (dd, J = 8.8, 2.0, 1H, ArH), 6.48 (d, J = 2.0, 1H, ArH), 3.81 (s, 3H, OCH_3), 1.75 (s, 6H, $C(CH_3)_2$)

^{13}C NMR (101 MHz, DMSO-d_6): δ_C 177.4, 176.7, 164.8, 160.9, 144.6 (HC=**C**H), 132.6 (Ar), 115.1, 113.1, 112.2, 112.1 (H**C**=CH), 111.6, 107.7 (Ar), 100.9 (Ar), 98.7, 94.8, 55.6 (OCH_3), 52.5, 25.4 (C(**C**H$_3$)$_2$)

IR (neat): 3250 (br), 2998, 2944, 2230, 1567, 1532, 1283

HRMS (ESI$^+$): Calc. for $C_{19}H_{16}N_3O_3$ $[M+H]^+$ 334.1186, found 334.1189

Spectroscopic data are consistent with those previously reported.[7]

Synthesis of 6c

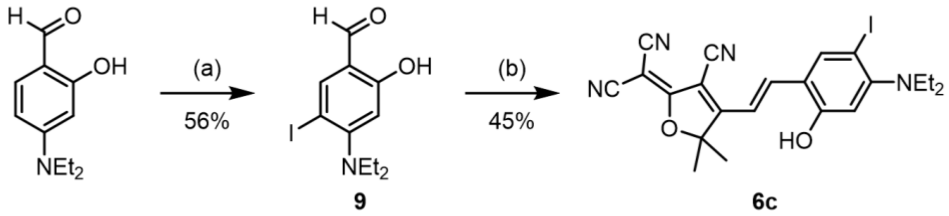

Reagents and conditions: (a) *N*-Iodosuccinimide, hexafluoroisopropanol, DCM, rt, 18 h; (b) **7**, NH_4OAc, EtOH, 78 °C, 18 h.

4-(Diethylamino)-2-hydroxy-5-iodobenzaldehyde (9)

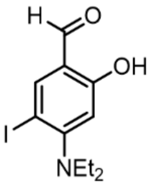

Prepared according to a modified literature procedure.[8] Hexafluoroisopropanol (1 mL) was added to a solution of 4-(diethylamino)-2-hydroxybenzaldehyde (193 mg, 1.0 mmol) and *N*-iodosuccinimide (225 mg, 1.0 mmol) in dichloromethane (4 mL) at room temperature. The mixture was stirred at room temperature for 18 hours, then the volatiles were evaporated under reduced pressure. The crude residue was purified by silica column chromatography (5:95 to 15:85 Et_2O:P.E., R_f = 0.49 at 10:90) to give the title compound (178 mg, 56%) as a yellow oil.

^{1}H NMR (400 MHz, $CDCl_3$): δ_H 11.11 (s, 1H, OH), 9.65 (d, *J* = 0.6, 1H, CHO), 7.93 (s, 1H, ArH), 6.54 (s, 1H, ArH), 3.21 (q, *J* = 7.1, 4H, C**H**$_2$CH$_3$), 1.10 (t, *J* = 7.1, 6H, CH$_2$C**H**$_3$)

^{13}C NMR (101 MHz, $CDCl_3$): δ_C 193.6 (CHO), 162.8 (Ar), 160.6 (Ar), 145.6 (Ar), 118.1 (Ar), 110.7 (Ar), 82.9 (Ar), 46.5 (NCH_2), 12.3 (CH_3)

IR (neat): 3112, 2972, 2931, 2833, 1639, 1610, 1207, 1195, 1128

HRMS (ESI$^+$): Calc. for $C_{11}H_{14}O_2NI$ $[M+H]^+$ 320.0142, found 320.0132

(*E*)-2-(3-cyano-4-(4-(diethylamino)-2-hydroxy-5-iodostyryl)-5,5-dimethylfuran-2(5H)-ylidene)malononitrile (6c)

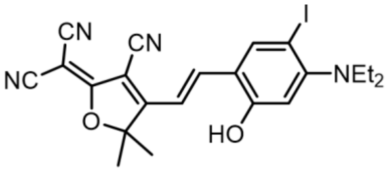

Prepared according to the general procedure using **7** (50 mg, 0.25 mmol), **9** (95 mg, 0.30 mmol) and NH_4OAc (4 mg, 0.05 mmol). The reaction mixture was evaporated to dryness, and the residue was redissolved in EtOAc and passed through a silica plug (eluting with EtOAc). The filtrate was concentrated, and the residue was recrystallised from EtOAc/hexane to give the title compound (56 mg, 45%) as a black solid.

^{1}H NMR (400 MHz, Acetone-*d*$_6$): δ 9.84 (br s, 1H, OH), 8.26 (s, 1H, ArH), 8.10 (d, *J* = 16.4, 1H, HC=C**H**), 7.49 (d, *J* = 16.4, 1H, **H**C=CH), 6.78 (s, 1H, ArH), 3.18 (q, *J* = 7.1, 4H, C**H**$_2$CH$_3$), 1.88 (s, 6H, C(CH$_3$)$_2$), 1.07 (t, *J* = 7.1, 6H, CH$_2$C**H**$_3$)

^{13}C NMR (101 MHz, Acetone-*d*$_6$): δ 177.7, 176.9, 159.9, 158.4, 143.4 (HC=**C**H), 142.8 (Ar), 120.1, 115.1 (H**C**=CH), 113.3, 112.5, 111.8, 111.4 (Ar), 99.3, 98.3, 86.4, 55.5, 47.3 (**C**H$_2$CH$_3$), 26.2 (C(**C**H$_3$)$_2$), 12.6

$(CH_2\mathbf{C}H_3)$

IR: 3307 (br), 2978, 2932, 2226, 1552, 1515, 1249, 1181

HRMS (ESI$^+$): Calc. for $C_{22}H_{21}O_2N_4I$ $[M+H]^+$ 501.0782, found 501.0768

Synthesis of 6d

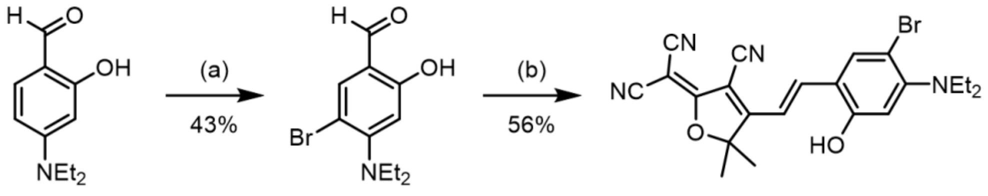

Reagents and conditions: (a) HBr (aq.), DMSO, EtOAc, 60 °C, 3 h; (b) **7**, NH_4OAc, EtOH, 78 °C, 18 h.

5-Bromo-4-(diethylamino)-2-hydroxybenzaldehyde (10)

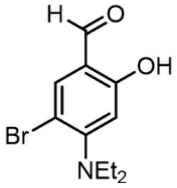

Prepared according to a modified literature procedure.[9] Aqueous hydrobromic acid (185 mg, 48 wt%, 0.55 mmol) was added to a solution of 4-(diethylamino)salicylaldehyde (97 mg, 0.5 mmol) and dimethyl sulfoxide (39 µL, 1.1 mmol) in EtOAc (2 mL). The mixture was stirred under air at 60 °C for 3 h. After cooling to room temperature, the mixture was diluted with EtOAc, washed successively with water and brine, then dried (Na_2SO_4), filtered and concentrated under reduced pressure. The crude product was purified by silica column chromatography (5:95 Et_2O:P.E., R_f = 0.19) to give the title compound (58 mg, 43%) as a yellow oil.

^{1}H NMR (400 MHz, CDCl$_3$): δ_H 11.13 (s, 1H, OH), 9.62 (d, *J* = 0.6, 1H, CHO), 7.63 (s, 1H, ArH), 6.49 (s, 1H, ArH), 3.29 (q, *J* = 7.1, 4H, CH_2), 1.13 (t, *J* = 7.1, 6H, CH_3)

^{13}C NMR (101 MHz, CDCl$_3$): δ_C 193.3 (C=O), 162.0 (Ar), 157.4 (Ar), 139.1 (Ar), 116.3 (Ar), 109.2 (Ar), 107.0 (Ar), 45.9 (CH_2), 12.5 (CH_3)

Spectroscopic data are consistent with those previously reported.[10]

(*E*)-2-(4-(5-Bromo-4-(diethylamino)-2-hydroxystyryl)-3-cyano-5,5-dimethylfuran-2(5H)-ylidene) malononitrile (6d)

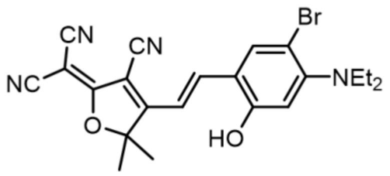

Prepared according to the general procedure, using **7** (50 mg, 0.25 mmol), **10** (68 mg, 0.3 mmol) and NH_4Cl (4 mg, 0.05 mmol), to give the title compound (63 mg, 56%) as a golden solid.

^{1}H NMR (400 MHz, Acetone-*d*$_6$): δ_H 8.14 (d, *J* = 16.3, 1H, HC=C**H**), 8.00 (s, 1H, ArH), 7.43 (d, *J* = 16.3, 1H, **H**C=CH), 6.77 (s, 1H (ArH), 3.28 (q, *J* = 7.0, 4H, C**H**$_2$CH$_3$), 1.87 (s, 6H, C(CH$_3$)$_2$), 1.11 (t, *J* = 7.0, 6H, CH$_2$C**H**$_3$).

^{13}C NMR (101 MHz, Acetone-*d*$_6$): δ_C 177.8, 176.9, 159.2, 155.3, 143.6 (HC=**C**H), 136.5 (Ar), 118.2, 114.5 (HC=**C**H), 113.3, 112.6, 111.9, 110.2 (Ar), 109.6, 99.2, 97.7, 55.3, 46.6 (**C**H$_2$CH$_3$), 26.2 (C(**C**H$_3$)$_2$), 12.7 (CH$_2$**C**H$_3$)

IR (neat): 3250, 2965, 2925, 2851, 2226, 1544, 1512, 1248, 1229

HRMS (ESI$^+$): Calc. for $C_{22}H_{22}(^{79}Br)N_4O_2$ $[M+H]^+$ 453.0921, found 453.0929

Synthesis of 6e

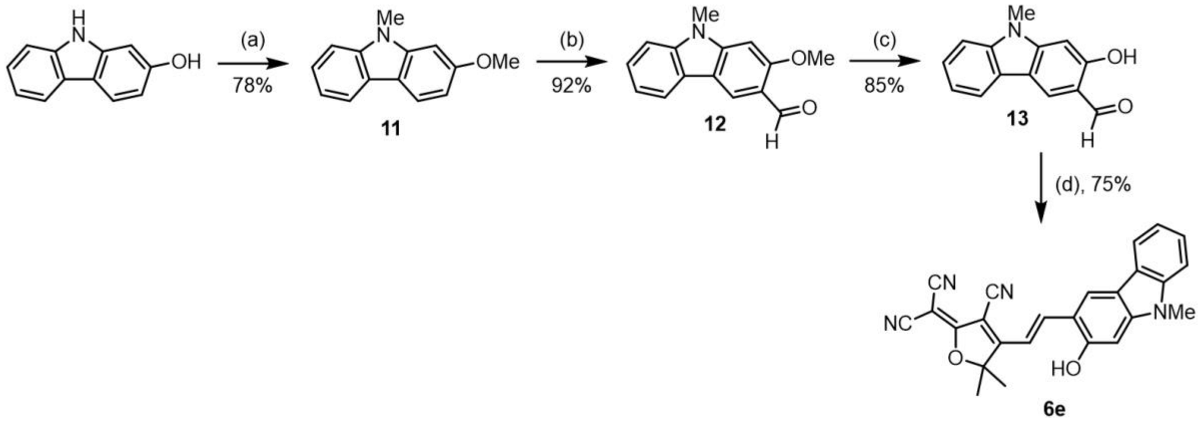

Reagents and conditions: (a) MeI, NaH, DMF, 0 °C to rt, 6 h; (b) $POCl_3$, DMF, 0 °C to 85 °C, 7.5 h; (c) BBr_3, DCM, 0 °C to rt, 20 h; (d) **7**, NH_4OAc, EtOH, 78 °C, 18 h.

2-Methoxy-9-methyl-9*H*-carbazole (11)

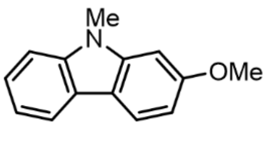

Synthesised according to a modified patent procedure.[11] 2-Hydroxycarbazole (413 mg, 2.25 mmol) and iodomethane (0.56 mL, 9.0 mmol) were dissolved in DMF (10 mL) and the solution was cooled to 0 °C. Sodium hydride (360 mg, 60 wt% dispersion in mineral oil, 15 mmol) was added in portions, then the mixture was stirred at room temperature for 6 h. The reaction was quenched by the addition of water (15 mL) and diluted with Et_2O (50 mL). The mixture was successively washed with water, aqueous LiCl (5 wt%) and brine, then dried ($MgSO_4$), filtered and concentrated under reduced pressure. The crude product was purified by silica column chromatography (1:9 Et_2O:P.E., R_f = 0.20) to give the title compound (372 mg, 78%) as an off-white solid.

^{1}H NMR (400 MHz, $CDCl_3$): δ_H 8.02–7.99 (m, 1H, ArH), 7.98–7.96 (m, 1H, ArH), 7.44–7.39 (m, 1H, ArH), 7.37–7.35 (m, 1H, ArH), 7.24–7.20 (m, 1H, ArH), 6.88–6.85 (m, 2H, ArH), 3.95 (s, 3H, CH_3), 3.81 (s, 3H, CH_3)

^{13}C NMR (101 MHz, $CDCl_3$): δ_C 159.3 (Ar), 142.5 (Ar), 141.2 (Ar), 124.5 (Ar), 123.1 (Ar), 121.2 (Ar), 119.6 (Ar), 119.1 (Ar), 116.8 (Ar), 108.3 (Ar), 107.4 (Ar), 93.0 (Ar), 55.8 (OCH_3), 29.2 (NCH_3)

Spectroscopic data are consistent with those previously reported.[12]

2-Methoxy-9-methyl-9*H*-carbazole-3-carbaldehyde (12)

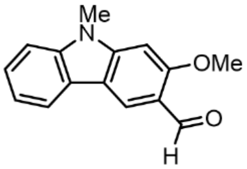

Phosphoryl chloride (0.17 mL, 1.78 mmol) was added dropwise to DMF (7 mL) at 0 °C. After stirring for 20 min at 0 °C, a solution of **11** (289 mg, 1.37 mmol) in DMF (3 mL) was added dropwise at 0 °C. The mixture was heated at 85 °C for 7.5 h, then cooled to room temperature and quenched by the addition of water (15 mL). The precipitate was collected, washed with water, and dried under vacuum to yield the title compound (301 mg, 92%) as a pale yellow solid.

^{1}H NMR (400 MHz, $CDCl_3$): δ_H 10.48 (s, 1H, CHO), 8.56 (s, 1H, ArH), 8.02 (ddd, J = 7.8, 1.2, 0.8, 1H, ArH), 7.45 (ddd, J = 8.3, 7.1, 1.2, 1H, ArH), 7.36–7.33 (m, 1H, ArH), 7.27 (ddd, J = 7.8, 7.3, 1.1, 1H, ArH), 6.73 (s, 1H, ArH), 4.03 (OCH_3), 3.80 (NCH_3)

^{13}C NMR (101 MHz, $CDCl_3$): δ_C 189.5 (CHO), 161.8 (Ar), 146.3 (Ar), 141.8 (Ar), 125.7 (Ar), 123.5 (Ar), 121.8 (Ar), 120.6 (Ar), 120.2 (Ar), 118.5 (Ar), 117.0 (Ar), 108.8 (Ar), 90.3 (Ar), 56.0 (OCH_3), 29.4 (NCH_3)

Spectroscopic data are consistent with those previously reported.[13]

2-Methoxy-9-methyl-9*H*-carbazole-3-carbaldehyde (13)

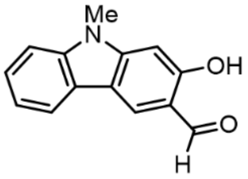

12 (371 mg, 1.55 mmol) was dissolved in dichloromethane (12 mL) and cooled to 0 °C. Boron tribromide (3.1 mL, 1 M in heptane, 3.1 mmol) was added dropwise and the mixture was stirred for 20 h at room temperature. The reaction was quenched by the addition of ice (10 g) and methanol (5 mL) whilst stirring. After the ice had melted, the biphasic mixture was diluted with water (10 mL) and separated. The aqueous layer was washed with DCM, and the combined organic layers were washed with aq. HCl (1 M) and brine, then dried (Na_2SO_4), filtered and evaporated under reduced pressure to give the title compound (296 mg, 85%) as a yellow-green solid.

^{1}H NMR (400 MHz, $CDCl_3$): δ_H 11.53 (s, 1H, CHO), 9.88 (d, *J* = 0.6, 1H, OH), 8.08 (s, 1H, ArH), 7.97 (ddd, *J* = 7.7, 1.2, 0.8, ArH), 7.46 (ddd, *J* = 8.2, 7.2, 1.2, ArH), 7.34 (dt, *J* = 8.2, 0.8, ArH), 7.29 (ddd, *J* = 7.7, 7.2, 1.0, ArH), 6.76 (ArH), 3.74 (s, 3H, NCH_3)

^{13}C NMR (101 MHz, $CDCl_3$): δ_C 195.1 (CHO), 161.4 (Ar), 146.9 (Ar), 142.2 (Ar), 127.2 (Ar), 126.1 (Ar), 123.2 (Ar), 120.8 (Ar), 119.8 (Ar), 117.3 (Ar), 115.0 (Ar), 109.0 (Ar), 95.0 (Ar), 29.5 (CH_3)

Spectroscopic data are consistent with those previously reported.[14]

(*E*)-2-(3-Cyano-4-(2-(2-hydroxy-9-methyl-9H-carbazol-3-yl)vinyl)-5,5-dimethylfuran-2(5*H*)-ylidene)malononitrile (6e)

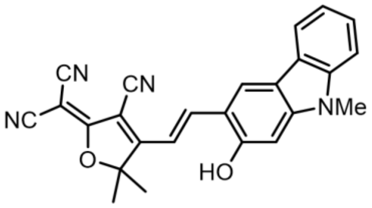

Prepared according to the general procedure using **7** (50 mg, 0.25 mmol), **13** (68 mg, 0.30 mmol) and NH_4Cl (4 mg, 0.05 mmol). The title compound (77 mg, 75%) was obtained as a dark red solid.

^{1}H NMR (400 MHz, Acetone-*d*$_6$): δ_H 8.70 (s, 1H, ArH), 8.52 (d, *J* = 16.3, 1H, HC=C**H**), 8.06 (ddd, *J* = 7.7, 1.2, 0.7, 1H, ArH), 7.53–7.49 (m, 2H, **H**C=CH + ArH), 7.44 (ddd, *J* = 8.2, 7.1, 1.2, 1H, ArH), 7.24 (ddd, *J* = 7.7, 7.1, 1.1, 1H, ArH), 3.84 (s, 3H, NCH_3), 1.91 (s, 6H, $C(CH_3)_2$).

^{13}C NMR (126 MHz, Acetone-*d*$_6$): δ_C 178.0, 177.2, 159.0, 147.0, 146.6, 143.0, 126.7, 123.9, 123.6, 121.3, 120.7, 119.0, 116.4, 113.5, 112.8, 112.7, 112.1, 110.2, 98.9, 96.2, 95.8, 54.5, 26.5

IR (neat): 3306 (br), 3057, 2990, 2225, 2175, 1497, 1235, 1204

HRMS (ESI+): Calc. for $C_{25}H_{18}N_4NaO_2$ $[M+Na]^+$ 429.1322, found 429.1307

(*E*)-2-(3-Cyano-4-(4-(diethylamino)-2-hydroxystyryl)-5,5-dimethylfuran-2(5H)-ylidene) malononitrile[15] (6f)

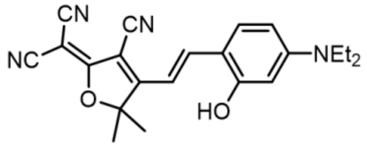

Prepared according to the general procedure, using **7** (50 mg, 0.25 mmol), 4-(diethylamino)salicylaldehyde (58 mg, 0.3 mmol) and NH_4Cl (4 mg, 0.05 mmol). The title compound (35 mg, 37%) was obtained as a dark blue solid.

^{1}H NMR (400 MHz, Acetone-*d*$_6$): δ_H 8.26 (d, *J* = 15.7, 1H, HC=C**H**), 7.68 (d, *J* = 9.2, 1H, ArH), 7.06 (d, *J* = 15.7, 1H, **H**C=CH), 6.50 (dd, *J* = 9.2, 2.5, 1H, ArH), 6.31 (d, *J* = 2.5, 1H, ArH), 3.52 (q, *J* = 7.1, 4H, C**H**$_2$CH$_3$), 1.80 (s, 6H, C(CH$_3$)$_2$), 1.22 (t, *J* = 7.1, 6H, CH$_2$C**H**$_3$)

^{13}C NMR (126 MHz, Acetone-*d*$_6$): δ_C 178.2, 176.7, 162.4, 155.0, 145.9 (HC=**C**H), 133.4 (Ar), 114.2, 113.5, 112.9, 112.6, 108.7 (H**C**=CH), 107.6 (Ar), 97.91, 96.85 (Ar), 91.5, 52.0, 45.6 (**C**H$_2$CH$_3$), 26.7 (C(**C**H$_3$)$_2$), 13.0 (CH$_2$**C**H$_3$)

IR (neat): 3269, 2980, 2935, 2221, 1486, 1240, 1176

HRMS (ESI+): Calc. for $C_{22}H_{22}N_4NaO_2$ $[M+Na]^+$ 397.1635, found 397.1640

Synthesis of 6g

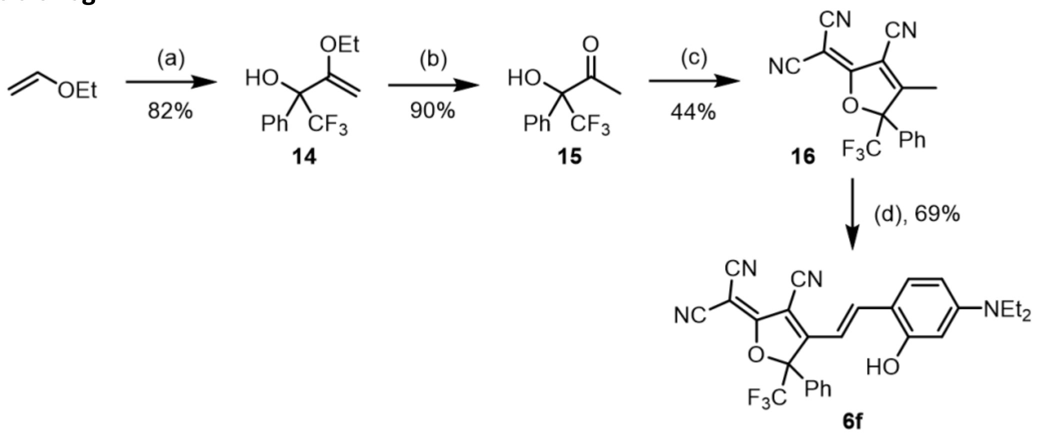

Reagents and conditions: (a) *t*BuLi, 2-MeTHF, -78 to -20 °C, 30 min, *then* CF_3COPh, 2-MeTHF, -78 °C, 2 h; (b) HCl (aq.), MeOH, rt, 3.5 h; (c) $CH_2(CN)_2$, NaOEt, EtOH, 100 °C, 1 h; (d) 4-(Diethylamino)salicylaldehyde, NH_4OAc, EtOH, 78 °C, 18 h.

3-Ethoxy-1,1,1-trifluoro-2-phenylbut-3-en-2-ol (14)

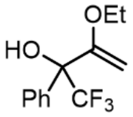

tert-Butyllithium (7.55 mL, 1.7 M in pentane, 12.8 mmol) was added dropwise over 10 minutes to a solution of ethyl vinyl ether (926 mg, 12.8 mmol) in anhydrous 2-MeTHF (12 mL) at -78 °C. The reaction vessel was transferred to a second cooling bath at -20 °C, and the mixture was stirred for 30 minutes while maintaining a temperature between -20 and -25 °C. The mixture was cooled to -78 °C and a solution of 2,2,2-trifluoroacetophenone (1.12 g, 6.4 mmol) in 2-MeTHF (3 mL) was added dropwise over 5 minutes. After stirring for 2 hours at -78 °C, the mixture was warmed to 0 °C and stirred for a further 30 minutes, then quenched by the addition of sat. aq. NH_4Cl. The biphasic mixture was diluted with water and extracted with Et_2O. The combined organic extracts were dried ($MgSO_4$), filtered and concentrated under reduced pressure. The crude product was purified by silica column chromatography (5:95 to 30:70 Et_2O:P.E., R_f = 0.36 at 20:80), affording the title compound (1.30 g, 82%) as a colourless oil.

^{1}H NMR (400 MHz, $CDCl_3$): δ_H 7.70–7.65 (m, 2H, ArH), 7.42–7.35 (m, 3H, ArH), 4.45 (d, *J* = 3.6, 1H, C=C**H**H), 4.38 (d, *J* = 3.6, 1H, C=CH**H**), 3.84 (app. qq, *J* = 9.6, 7.0, 2H, CH_2), 3.68 (br s, 1H, OH), 1.31 (t, *J* = 7.0, 3H, CH_3)

^{13}C NMR (101 MHz, $CDCl_3$): δ_C 157.7 (**C**=CH_2), 136.7 (Ar), 128.9 (Ar), 128.2 (Ar), 127.1 (q, *J* = 1.6, Ar), 124.5 (q, *J* = 286.1, CF_3), 86.8 (q, *J* = 1.9, C=**C**H_2), 78.6 (q, *J* = 29.0, **C**CF_3), 64.2 (CH_2), 14.2 (CH_3)

IR (neat): 3550 (br), 2985, 1262, 1160, 1063

HRMS (APCI$^+$): Calc. for $C_{12}H_{14}O_2F_3$ $[M+H]^+$ 247.0940, found 247.0937

4,4,4-Trifluoro-3-hydroxy-3-phenylbutan-2-one (15)

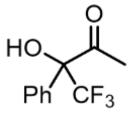

Aqueous HCl (10.4 mL, 1 M, 10.4 mmol) was added to a solution of **14** (1.28 g, 5.2 mmol) in methanol (10 mL). The mixture was stirred at room temperature for 3.5 hours, then concentrated under reduced pressure and extracted with Et_2O. The combined organic extracts were dried ($MgSO_4$) filtered and concentrated under reduced pressure to give the title compound (1.02 g, 90%) as a colourless oil, which was used without further purification.

^{1}H NMR (400 MHz, $CDCl_3$): δ_H 7.61–7.57 (m, 2H, ArH), 7.47–7.41 (m, 3H, ArH), 4.97 (s, 1H, OH), 2.32 (q, *J* = 0.9, 3H, CH_3)

^{13}C NMR (101 MHz, $CDCl_3$): δ_C 201.6 (C=O), 133.4 (Ar), 129.7 (Ar), 129.2 (Ar), 126.6 (q, J = 2.1, Ar), 123.7 (q, J = 286.1, CF_3), 82.6 (q, J = 28.8, **C**CF_3), 25.3 (q, J = 2.4, CH_3)

Spectroscopic data are consistent with those previously reported.[16]

2-(3-Cyano-4-methyl-5-phenyl-5-(trifluoromethyl)furan-2(5H)-ylidene)malononitrile (16)

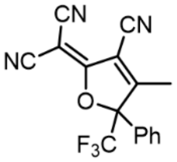

15 (252 mg, 1.16 mmol) and malononitrile (160 mg, 2.43 mmol) were added to an ethanolic solution of sodium ethoxide (0.50 mL, 0.12 mmol, prepared by dissolving sodium metal [10.6 mg, 0.46 mmol] in 2.0 mL ethanol) in a microwave vial with a Teflon-coated stirrer bar. The sealed vial was heated at 100 °C under microwave irradiation for 1 hour. The mixture was diluted with aq. HCl (0.5 M), then extracted with chloroform. The combined organic layers were dried ($MgSO_4$), filtered and concentrated under reduced pressure. The crude product was purified by silica column chromatography (1:1 acetone:P.E., R_f = 0.24) to give a salt of **16**, which was added to aq. HCl (1 M) and extracted with chloroform. The combined organic extracts were dried ($MgSO_4$), filtered and evaporated under reduced pressure to give the racemic title compound (159 mg, 44%) as a dark green solid.

^{1}H NMR ($CDCl_3$, 400 MHz): δ_H 7.61–7.52 (m, 3H, ArH), 7.45–7.41 (m, 2H, ArH), 2.48 (s, 3H, CH_3)

^{13}C NMR ($CDCl_3$, 101 MHz): δ_C 174.1, 172.0, 131.9, 130.2, 127.4, 125.7 (q, J = 1.9, Ar), 121.6 (q, J = 287.4, CF_3), 109.8 (q, J = 1.2), 109.4, 109.0, 108.0, 98.4 (q, J = 32.2, **C**CF_3), 63.1, 15.4 (q, J = 1.6, CH_3)

Spectroscopic data are consistent with those previously reported.[17]

(*E*)-2-(3-Cyano-4-(4-(diethylamino)-2-hydroxystyryl)-5-phenyl-5-(trifluoromethyl)furan-2(5H)-ylidene)malononitrile (6g)

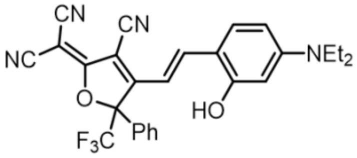

Prepared according to the general procedure, using **16** (79 mg, 0.25 mmol), 4-(diethylamino)salicylaldehyde (58 mg, 0.3 mmol) and NH_4Cl (4 mg, 0.05 mmol). The title compound (85 mg, 69%) was obtained as a metallic-green solid.

^{1}H NMR ($CDCl_3$, 400 MHz): δ_H 8.95 (br s, 1H, HC=C**H**), 8.54 (br s, 1H, OH), 7.56–7.45 (m, 5H, ArH), 7.40

(br d, *J* = 6.0, 1H, ArH), 6.48 (br s, 1H, **H**C=CH), 6.36 (dd, *J* = 9.2, 2.4, 1H, ArH), 6.17 (d, *J* = 2.4, 1H, ArH), 3.52 (q, *J* = 7.2, 4H, C**H**$_2$CH$_3$), 1.25 (t, *J* = 7.2, 6H, CH$_2$C**H**$_3$)

^{13}C NMR (CDCl$_3$, 126 MHz): δ_C 177.5, 163.9, 161.4, 156.8, 145.9, 131.3, 131.0 (Ar), 129.5 (Ar), 126.7 (Ar), 122.5 (q, *J* = 286.1, CF$_3$), 114.9, 113.9, 113.0, 112.8, 109.3 (Ar), 96.8 (Ar), 95.1 (q, *J* = 31.6), 51.6, 46.0 (**C**H$_2$CH$_3$), 13.1 (CH$_2$**C**H$_3$)

IR (neat): 3266 (br), 2979, 2928, 2221, 1482, 1173, 1141

HRMS (ESI$^+$): Calc. for $C_{27}H_{22}N_4O_2F_3$ [M+H]$^+$ 491.1689, found 419.1679

S3 Stock solution preparation

Stock solutions of the CD receptor (Zn(**3**)·2ClO$_4$), ligands (**4**-H and **5**-H) and compounds **6a,c,d,f,g** were prepared as 2.50 mM solutions by weighing out the appropriate mass of compound in a 5 mL volumetric flask, then dissolving in anhydrous MeCN. Compounds **6b** and **6e** were instead prepared as 1.25 mM solutions due to their poorer solubility in MeCN. For triethylamine, an arbitrary amount (20-40 mg) of compound was weighed out in a pre-weighed, stoppered 5 mL volumetric flask (to minimise compound evaporation and atmospheric water absorption) and dissolved in MeCN to give a solution of arbitrary, but known, concentration. An aliquot of this solution was then diluted to the desired concentration (2.50 mM) by the addition of an appropriate volume of solvent, as required.

S4 Additional CD spectroscopy methodology

S4.1 Light-induced ligand exchange with **6a-g** (Fig. 3)

For each dye, an initial sample was prepared by mixing solutions of Zn(**3**)·2ClO$_4$ (20 µL, 2.50 mM, 50 nmol), **4**-H (20 µL, 2.50 mM, 50 nmol), **5**-H (20 µL, 2.50 mM, 50 nmol), NEt$_3$ (40 µL, 2.50 mM, 100 nmol), dye (20 µL, 2.50 mM, 50 nmol for **6a**, **6c**, **6d**, **6f** and **6g**, or 40 µL, 1.25 mM, 50 nmol for **6b** and **6e**), MeOH (100 µL) and made up to 2000 µL with MeCN. Each sample was allowed to equilibrate in the dark inside the CD spectrometer while θ_{240} was recorded over 5 minutes. After the equilibration period, each sample was placed into the irradiation box and irradiated for 10 seconds (1000 mA current) at the appropriate wavelength: 405 nm (**6a**), 470 nm (**6b** and **6c**), 530 nm (**6d** and **6e**), 554 nm (**6f**), 625 nm (**6g**). Each sample was then immediately placed back inside the CD spectrometer and θ_{240} was recorded over 5 minutes. Only samples with **6a** or **6e** displayed significant light-induced shifts in θ_{240} (**Fig. 3, main article**); time-course CD data for samples containing **6b**, **6c**, **6d**, **6f** or **6g** are shown below (Fig. **S2**).

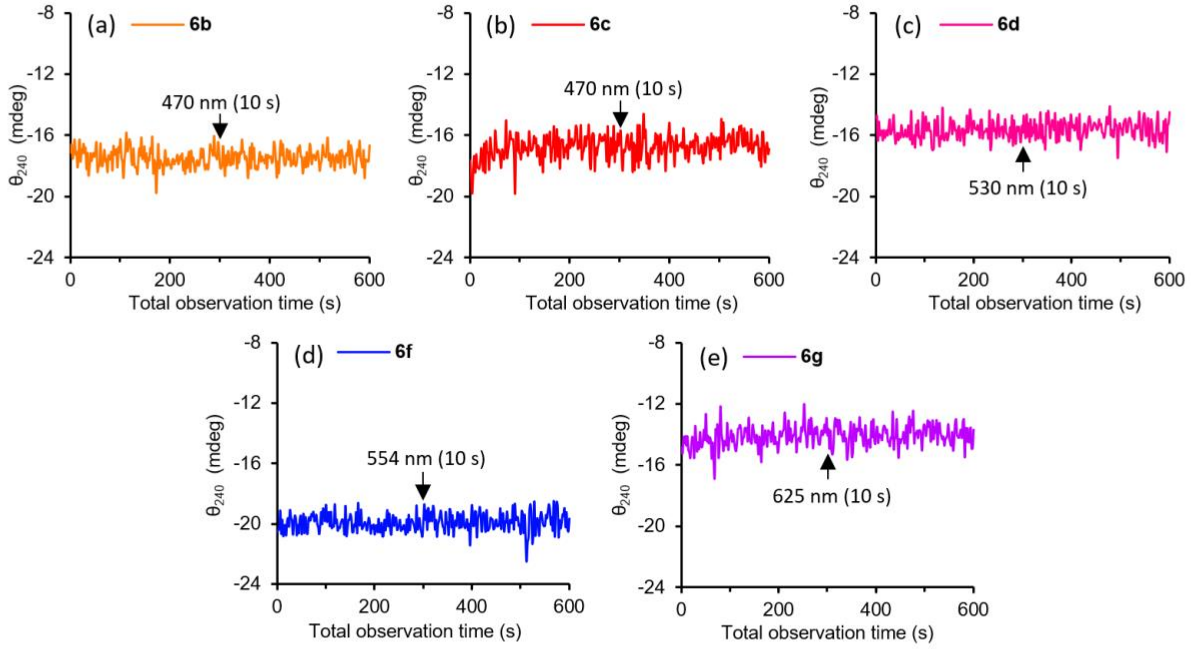

Figure S2 Time course CD spectroscopy measurements at 240 nm of Zn(**3**)·2ClO$_4$ (25 µM), **4**-H (1 equiv.), **5**-H (1 equiv.), NEt$_3$ (2 equiv.) with (a) **6b**, (b) **6c**, (c) **6d**, (d) **6f** or (e) **6g** in MeCN containing 5 vol% MeOH. Black arrows indicate a 10 second pulse of LED light of the given wavelength.

S4.2 Optimisation of irradiation conditions

The effect of varying the irradiation wavelength, LED power and irradiation duration on the isomerisation of photoacids **6a** and **6e** was examined, to find conditions where each photoacid could be selectively photoisomerised in the presence of the other. An initial sample for each photoacid was prepared by mixing solutions of Zn(**3**)·$2ClO_4$ (20 µL, 2.50 mM, 50 nmol), **4**-H (20 µL, 2.50 mM, 50 nmol), **5**-H (20 µL, 2.50 mM, 50 nmol), NEt_3 (40 µL, 2.50 mM, 100 nmol), photoacid (20 µL, 2.50 mM, 50 nmol for **6a**, or 40 µL, 1.25 mM, 50 nmol for **6e**), MeOH (100 µL) and made up to 2000 µL with MeCN. The mixtures were allowed to equilibrate in the dark for 5 minutes.

Each sample was irradiated for 10 seconds with 405 nm light while the LED controller was set to the maximum input current of 1000 mA, then immediately placed into the CD spectrometer and θ_{240} was recorded over 5 minutes. This process was repeated at lower input currents (500 mA, 250 mA, 125mA and 50 mA) and with different irradiation wavelengths (470 nm and 530 nm). The sample containing photoacid **6a** displayed comparable shifts in θ_{240} after irradiation with 405 nm (Fig. **S3a**) or 470 nm light (Fig. **S3c**) at every input current tested, while the shifts in θ_{240} for the sample containing photoacid **6e** decreased as the input current was decreased for both 405 nm (Fig. **S3b**) and 470 nm (Fig. **S3d**) light. Therefore, **6a** could be selectively isomerised in the presence of **6e** with 405 nm light by using a low input current (50 mA).

However, diminishing shifts in θ_{240} were observed for both **6a**- (Fig. **S3e**) and **6e**-containing samples (Fig. **S3f**) as the input current of the 530 nm LED was decreased from 1000 mA to 50 mA, indicating that **6e** could not be selectively isomerised by simply reducing the input current. Due to this poor selectivity of photoacid isomerisation, a 530 nm bandpass filter was attached to the LED to reduce the spectral overlap with the absorbance profile of **6a**. Irradiation for 10 seconds with filtered 530 nm light (1000 mA current) gave negligible shifts in θ_{240} both **6a**- (Fig. **S3g**) and **6e**-containing samples (Fig. **S3h**). Increasing the irradiation time to 30 seconds and 60 seconds appeared to give greater shifts in θ_{240} for the **6e**-containing sample than the **6a**-containing sample, though the noise in the CD response made data interpretation problematic.

These irradiation conditions were tested on a sample containing both **6a** and **6e** and instead analysed by UV-vis spectroscopy (**Fig. 5, main article**). A sample composed of Zn(**3**)·$2ClO_4$ (20 µL, 2.50 mM, 50 nmol), **4**-H (20 µL, 2.50 mM, 50 nmol), **5**-H (20 µL, 2.50 mM, 50 nmol), NEt_3 (40 µL, 2.50 mM, 100 nmol), **6a** (20 µL, 2.50 mM, 50 nmol), **6e** (40 µL, 1.25 mM, 50 nmol), MeOH (100 µL) and made up to 2000 µL with MeCN. After 5 minutes of equilibration in the dark, the UV-vis spectrum of the sample was acquired. As discussed in the main article, subsequent irradiation with 405 nm light (50 mA, 30 seconds) gave primarily loss of the **6a** absorbance band, while irradiation with filtered 530 nm light (1000 mA, 30 seconds) gave primarily loss of the **6e** absorbance band, confirming the selective isomerisation of **6e** under these conditions. Finally, simultaneous irradiation with 405 nm (50 mA, 30 seconds) and filtered 530 nm (1000 mA, 30 seconds), or monochromatic irradiation with 470 nm (125 mA, 30 seconds) led to isomerisation of both **6a** and **6e**.

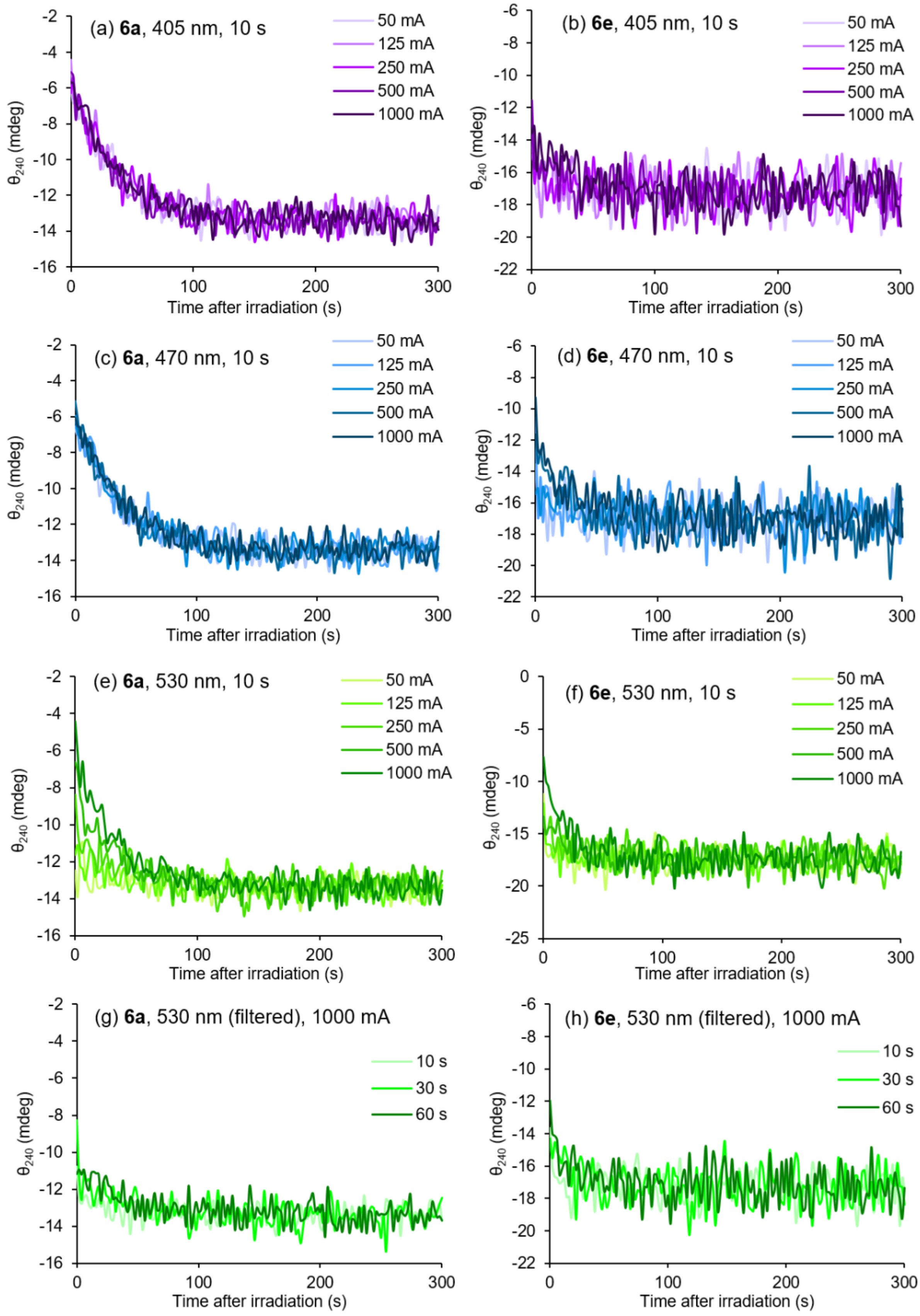

Figure S3: Time course CD spectroscopy measurements at 240 nm of Zn(**3**)·2ClO$_4$ (25 µM), **4**-H (1 equiv.), **5**-H (1 equiv.), NEt$_3$ (2 equiv.) in MeCN containing 5 vol% MeOH, following 10–60 seconds of irradiation (50–1000 mA input current) at (a) 405 nm with **6a** (1 equiv.), (b) 405 nm with **6e** (1 equiv.), (c) 470 nm with **6a** (1 equiv.), (d) 470 nm with **6e** (1 equiv.), (e) 530 nm with **6a** (1 equiv.), (f) 530 nm with **6e** (1 equiv.), (g) bandpass-filtered 530 nm light with **6a** or (h) bandpass-filtered 530 nm light with **6e**.

S4.3 Association constant measurements

Titration experiments were carried out with Zn(**3**)·2ClO$_4$ using 1:1 mixtures of ligand (**4**-H or **5**-H) and triethylamine to estimate the effective binding constants. A 2000 µL sample of Zn(**3**)·2ClO$_4$ (c = 25.0 µM, 5:95 MeOH:MeCN) in a 10 mm path length cuvette was titrated with a solution composed of ligand (**4**-H or **5**-H, 1.50 mM), triethylamine (1.50 mM), Zn(**3**)·2ClO$_4$ (25.0 µM) in 5:95 MeOH:MeCN at 25 °C. The CD response at 240 nm (θ_{240}) was recorded after addition of ligand solution, for a total of 18 data points up to 120 µL of ligand solution (3.4 equiv.). For **4**-H, this process was repeated for a total of 5 independent runs and the titration data was fit to a 1:1 host:guest binding model using Bindfit.[18] A representative binding isotherm is shown in fig. **S4a**. An average of five runs gave an effective binding constant of K = 7.2 ±1.9 ×10^5 M^{-1} where the uncertainty is the standard deviation (Table **S1**).

The titration data with **5**-H could not be adequately fit to 1:1, 1:2 or 2:1 host:guest binding models using Bindfit (Fig. **S4b**). Although the 2:1 fit appeared to match the experimental data visually, implausible binding constant values and extremely large errors given by the fitting software (59.2 M^{-1} ±346% for K_{11} and 2.86×10^8 M^{-1} ±365% for K_{21}) indicated that this was not an appropriate binding model. Therefore, the precise binding interaction between **5**$^-$ and [Zn(**3**)]$^{2+}$ was not determined.

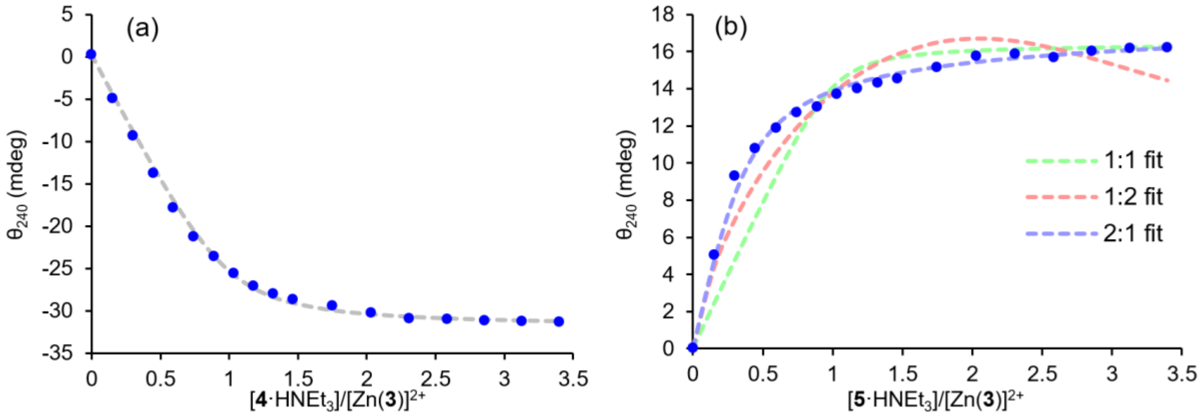

Figure S4: (a) Experimental data (blue points) and 1:1 fit (grey dashed line) from a titration of Zn(**3**)·2ClO$_4$ (c = 25.0 µM) with a 1:1 mixture of ligand **4**-H and NEt$_3$ in 5:95 MeOH:MeCN at 25 °C, monitoring the CD response at 240 nm. (b) Experimental data (blue points) and 1:1 (green dashed line), 1:2 (red dashed line) and 2:1 (blue dashed line) host:guest fits from a titration of Zn(**3**)·2ClO$_4$ (c = 25.0 µM) with a 1:1 mixture of ligand **5**-H and NEt$_3$ in 5:95 MeOH:MeCN at 25 °C, monitoring the CD response at 240 nm. All fits performed using Bindfit.

Table S1: Results from fitting data from titration of Zn(**3**)·2ClO$_4$ (c = 25.0 µM) with 1:1 mixture of ligand **4**-H and triethylamine in 5:95 MeOH:MeCN at 25 °C to a 1:1 binding model using BindFit.[18]

**Run**	**K_{assoc} (10^5 M^{-1})**
1	7.51
2	5.90
3	9.09
4	9.12
5	4.32
Mean	7.19
Standard deviation	1.86

S5 Calculation of pK_a values

The pK_a values of compounds **6a** and **6e** were determined using COSMO-RS method with empirical corrections.[19–21] Starting geometries of all possible conformers of protonated and deprotonated *E*- and *Z*-isomers, as well as cyclised anions, were created. The geometries were optimised at DFT RI-BP86/def-TZVP level of theory with COSMO model ($e = \infty$). Frequency analysis was carried out to ensure that the optimised structures correspond to local energy minima (evidenced by absence of negative frequencies in the spectrum). Then, single-point calculation was carried out at RI-BP86/def2-TZVPD/COSMO level with Fine cavity parameter. The results – geometries, energies and partial charge distributions on molecular surface – serve as the input for the following COSMOtherm calculation. All DFT calculations were carried out using Turbomole V7.7 software.[22]

The pK_a values in acetonitrile were calculated using COSMOtherm software (release 2023)[23] with parametrisation BP_TZVPD_FINE_23, taking all conformers into account. The software assigns weights to the conformers/isomers according to their calculated relative energies in the solution in question. Both cyclised (SP^-) and non-cyclised (Z^-) forms of anions were taken into account in the pK_a calculations. As expected, in acetonitrile the weights of *Z*-forms are insignificant compared to *SP*-forms.

To evaluate and correct the systematic error of computational values, the correlation was created between experimental[24] and corresponding computational pK_a values of 12 various phenolic compounds with pK_a values ranging from 14.2 to 29.2. The computed pK_a values of **6a** and **6e** were corrected using the obtained regression equation. The standard uncertainty of the results for *E*-isomers was estimated from the standard error of regression (0.5 pK_a units). Uncertainties of pK_a values of *Z*-isomers were conservatively estimated as higher (0.8 pK_a units), because none of the reference compounds form cycles upon (de)protonation.

Accurate prediction of the effect of 5 vol% of methanol on pK_a values would require extensive additional computations, because preferential solvation of anions by methanol molecules likely takes place. However, we can get approximate estimates of the effect from free energies of species in solutions computed by COSMO-RS.

The change of an acid's pK_a upon addition of methanol to acetonitrile can be rationalised via two phenomena: (1) change in solvation of the proton, and (2) change in solvation of the anion and, to a lesser degree, the neutral acid molecule. Both are improved by addition of a polar protic solvent like methanol. The first effect is constant for all acids and can be estimated from the free energies of proton transfer between solvents; in the present case this effect is in the range of –3 to –5 pK_a units. The second effect depends on the acid and is the stronger the more localised is the charge on its anion.[25] The calculations show that solvation of cyclic anions (SP^-) is almost unaffected by the added methanol. Anions of *E*-isomers (and non-cyclised anions of *Z*-isomers), however, have charge hotspots accessible to solvent molecules, so their solvation free energy becomes more negative upon addition of methanol. The pK_a values of *E*-isomers will be lowered by 0.5-0.7 units in addition to the systematic

effect (1). The pK_a value of carboxylic acid **4-H** is expected to be lowered even more strongly by adding methanol.

S6 ^{1}H and ^{13}C NMR spectra of photoacids and intermediates

^{1}H NMR spectrum (400 MHz, DMSO-d_6) of compound **6a**

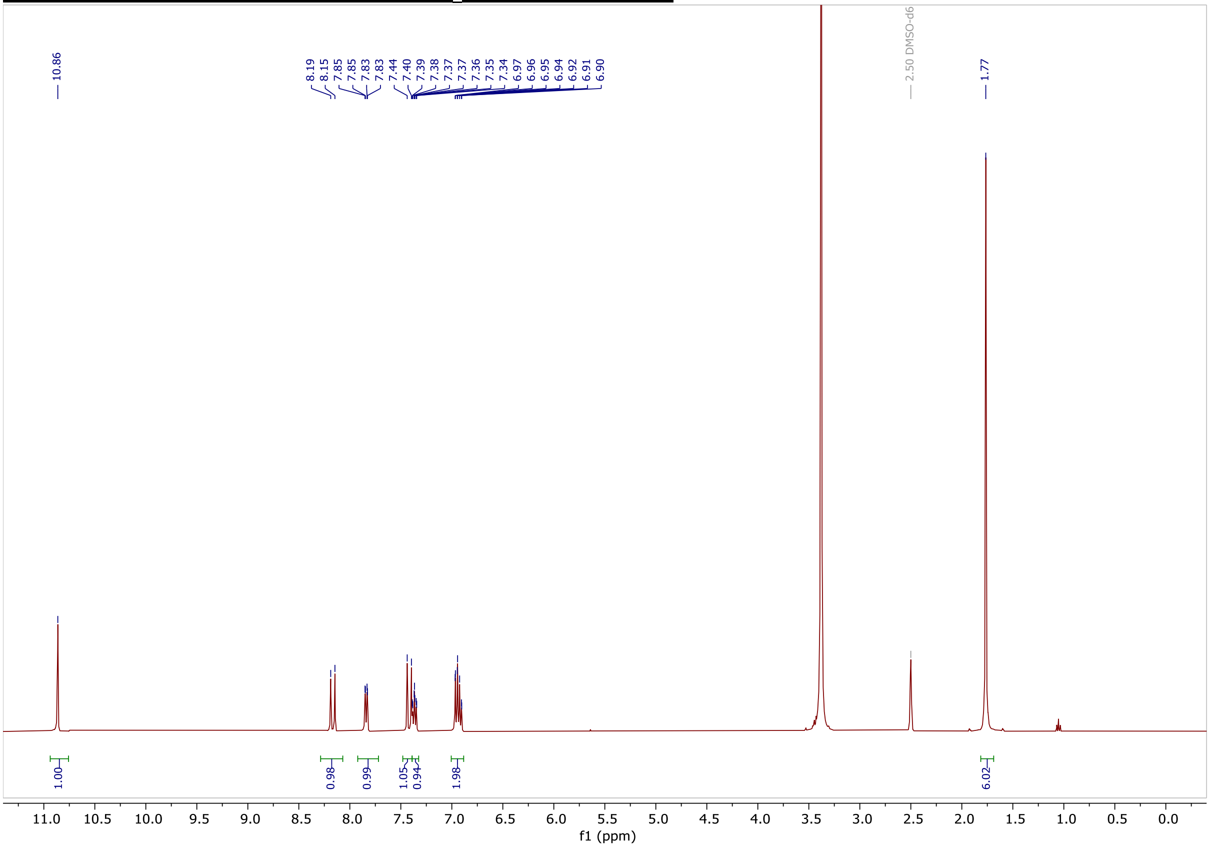

^{13}C NMR spectrum (101 MHz, DMSO-d_6) of compound **6a**

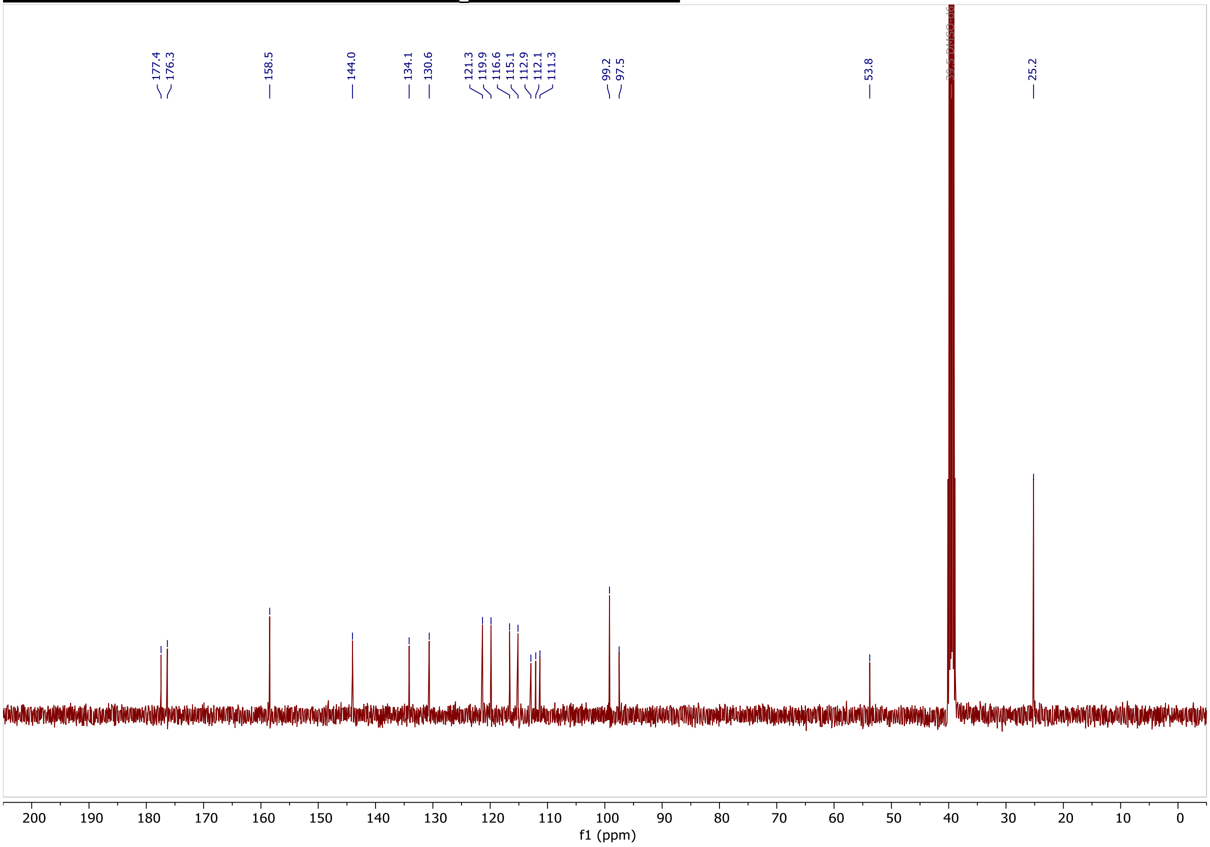

^{1}H NMR spectrum (400 MHz, DMSO-d_6) of compound **6b**

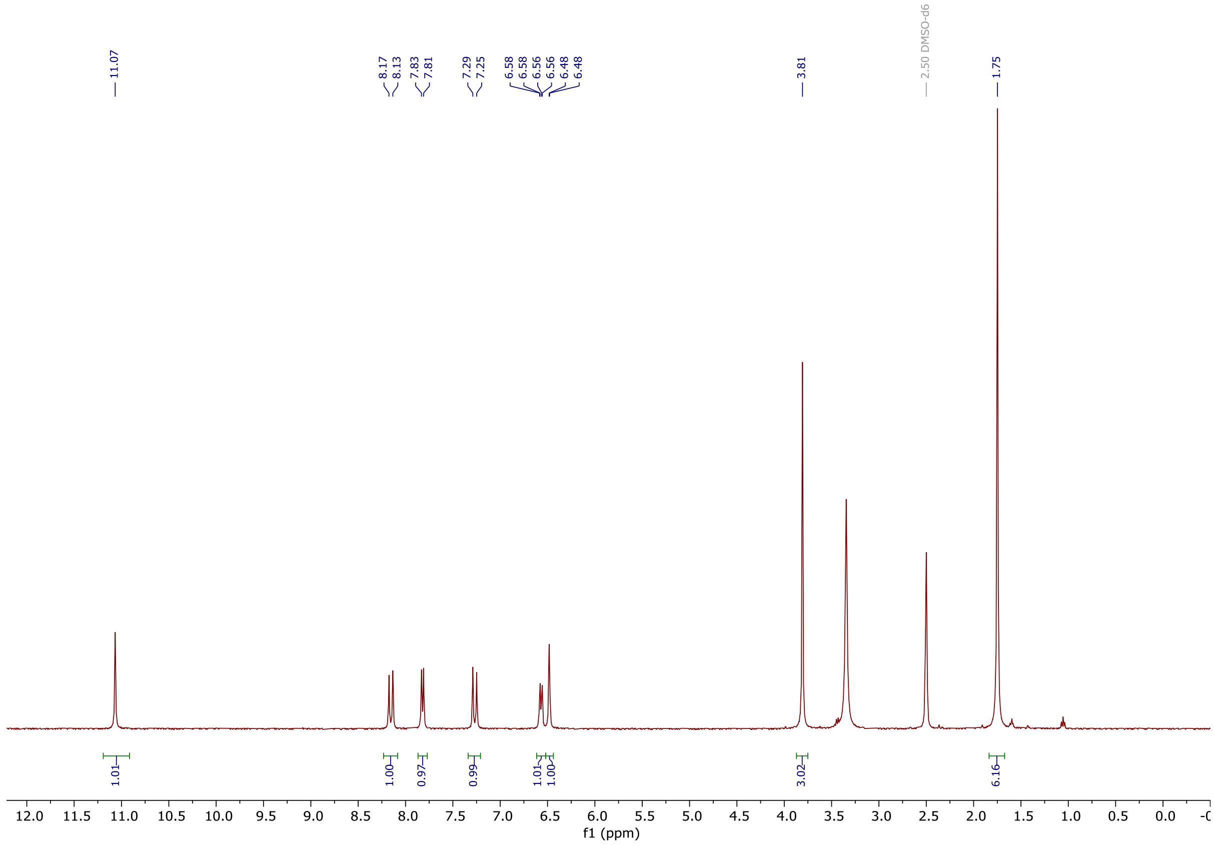

^{13}C NMR spectrum (101 MHz, DMSO-d_6) of compound **6b**

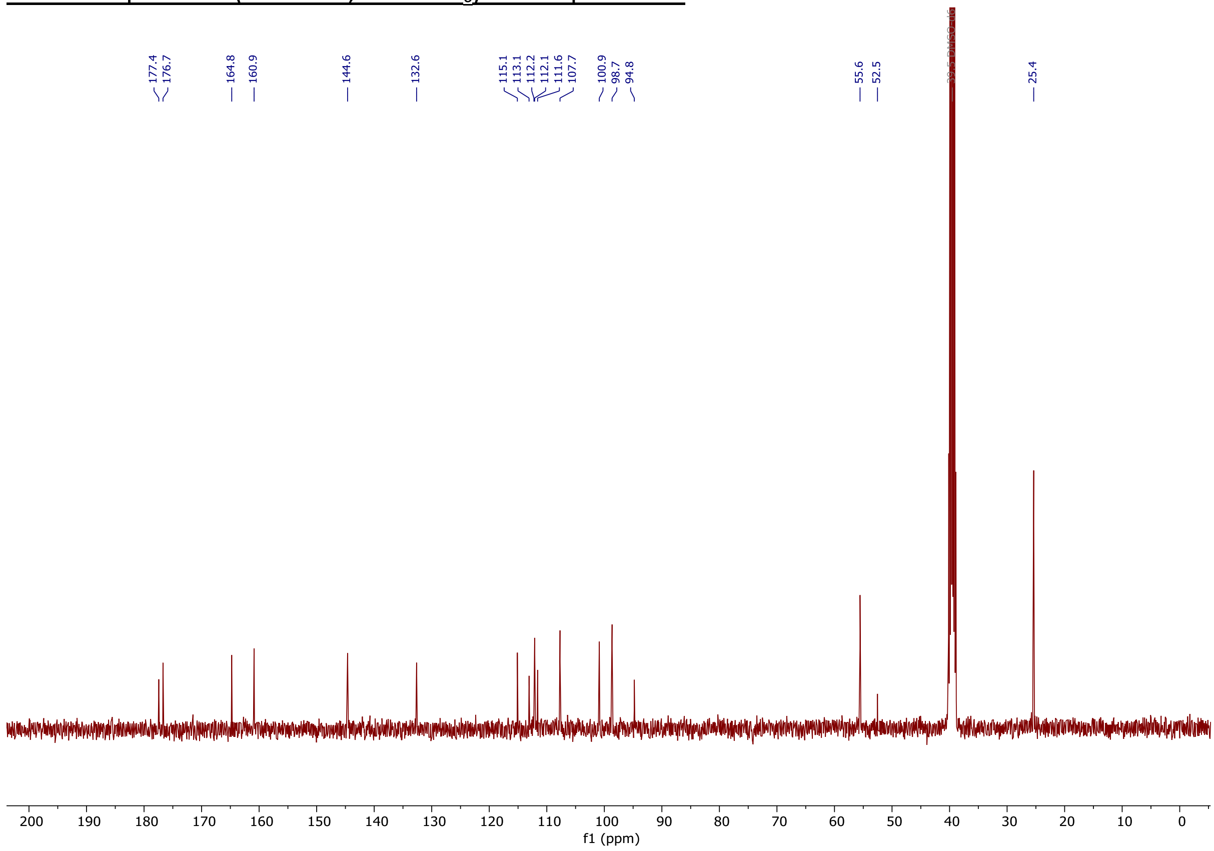

^{1}H NMR spectrum (400 MHz, Acetone-d_6) of compound **6c**

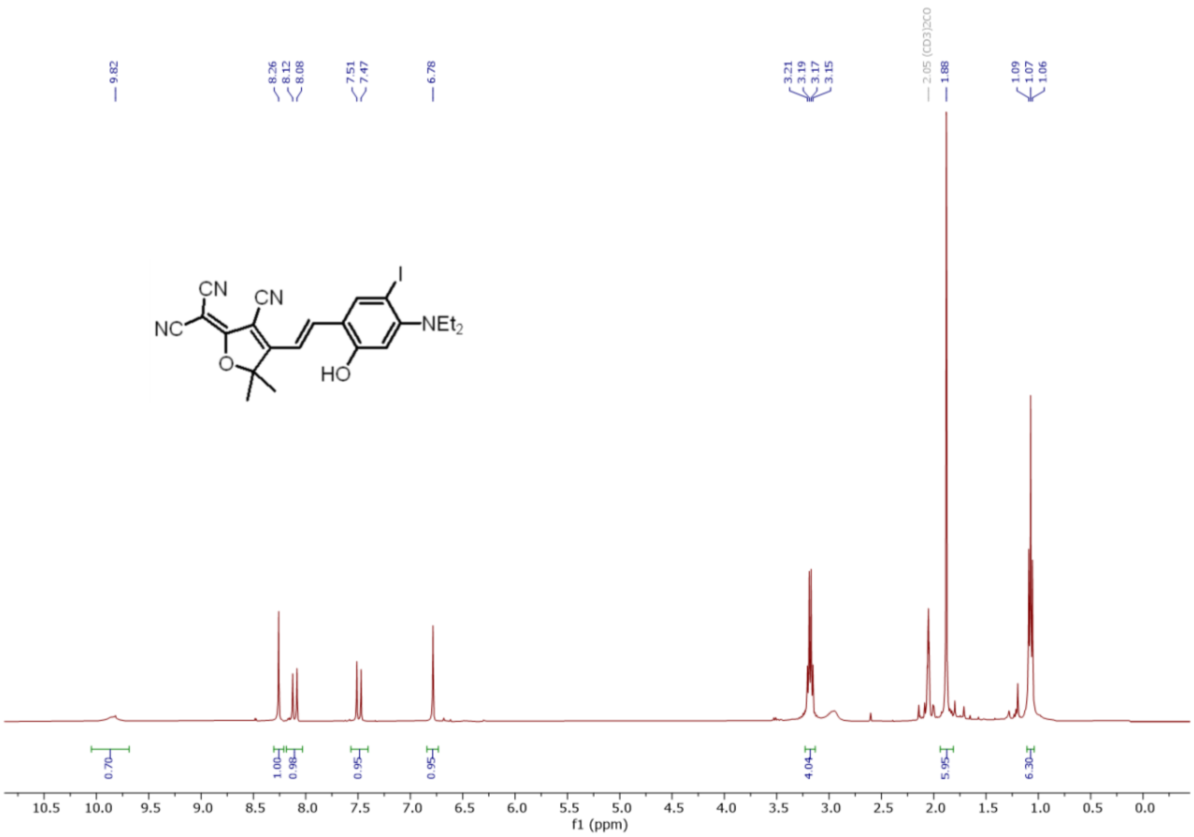

^{13}C NMR spectrum (101 MHz, Acetone-d_6) of compound **6c**

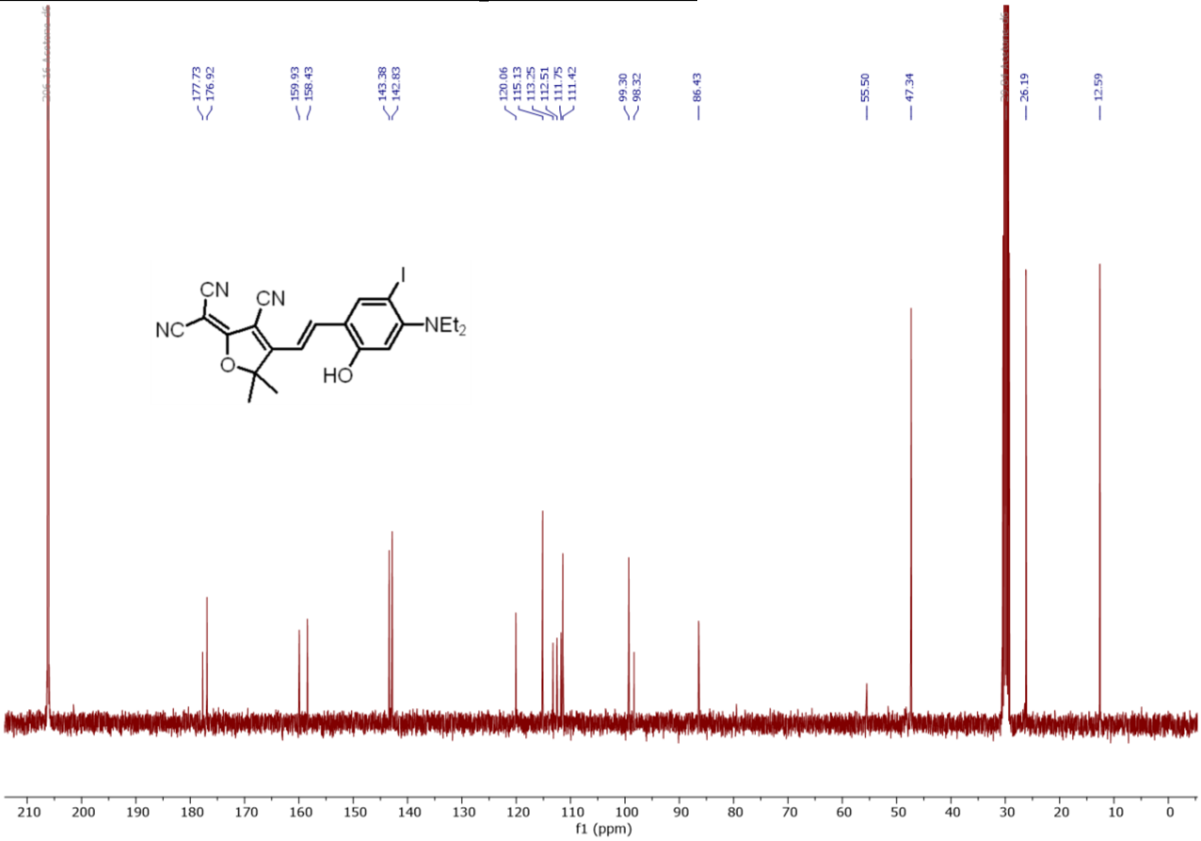

^{1}H NMR spectrum (400 MHz, Acetone-d_6) of compound **6d**

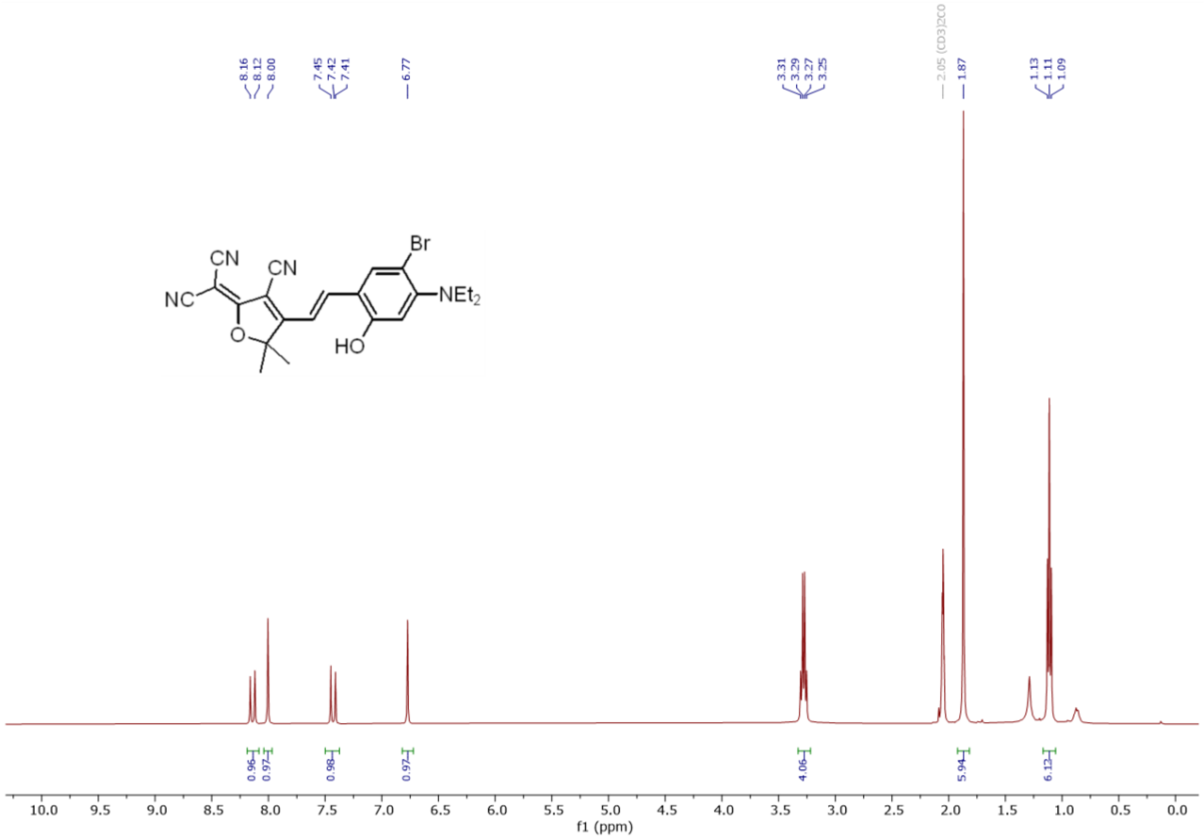

^{13}C NMR spectrum (101 MHz, Acetone-d_6) of compound **6d**

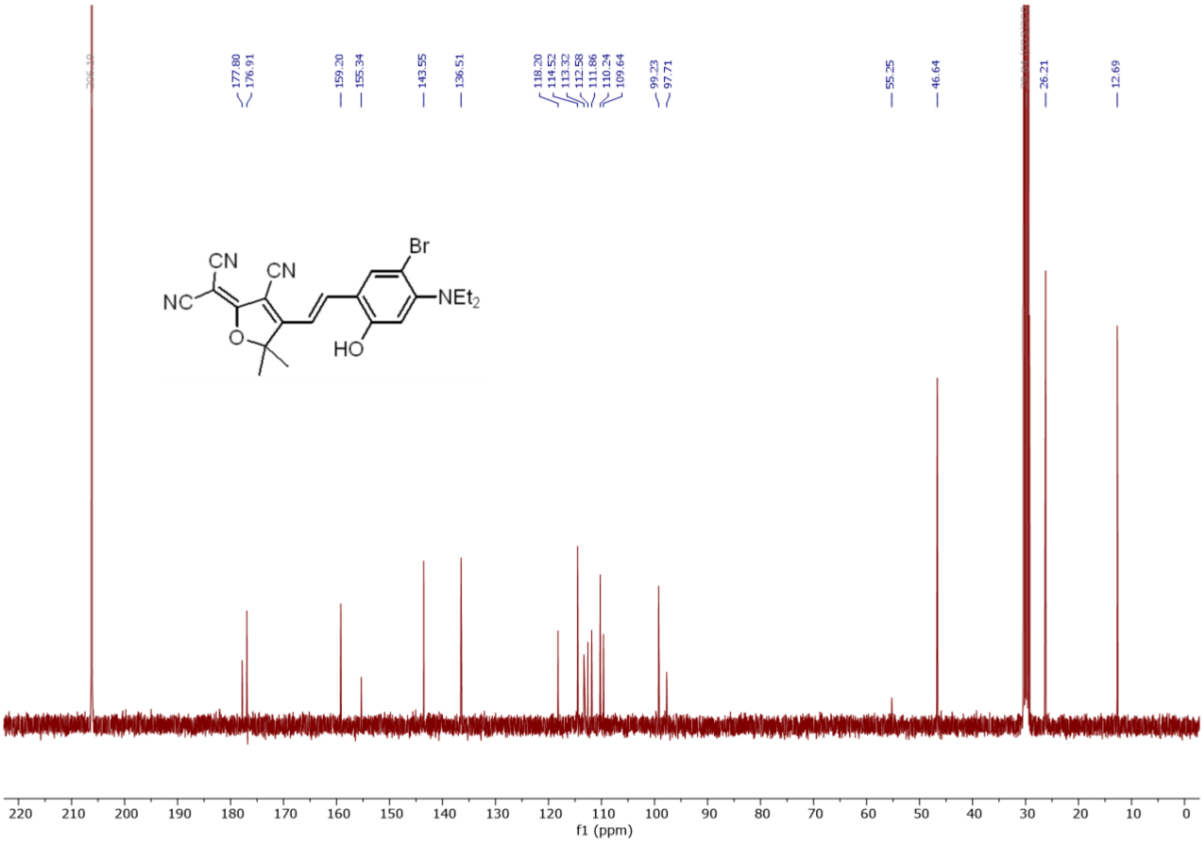

^{1}H NMR spectrum (400 MHz, Acetone-d_6) of compound **6e**

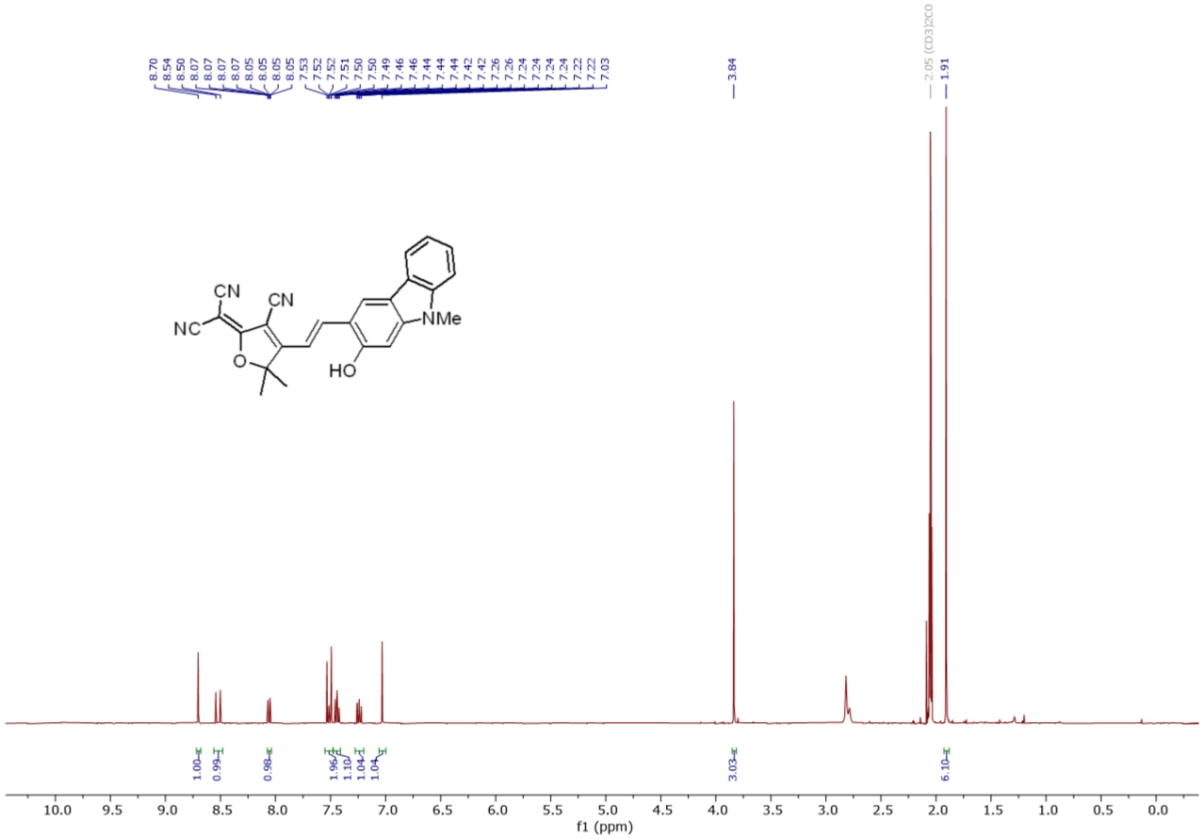

^{13}C NMR spectrum (126 MHz, Acetone-d_6) of compound **6e**

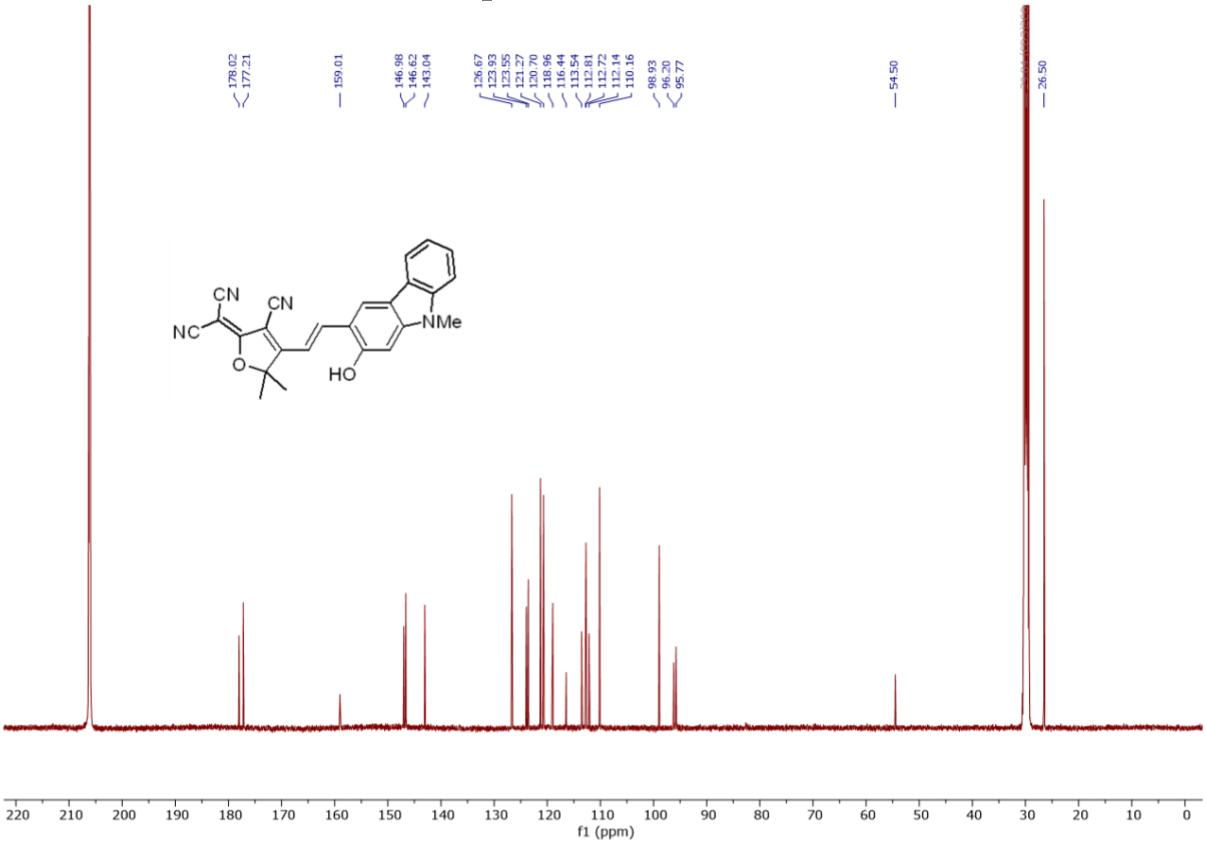

^{1}H NMR spectrum (400 MHz, Acetone-d_6) of compound **6f**

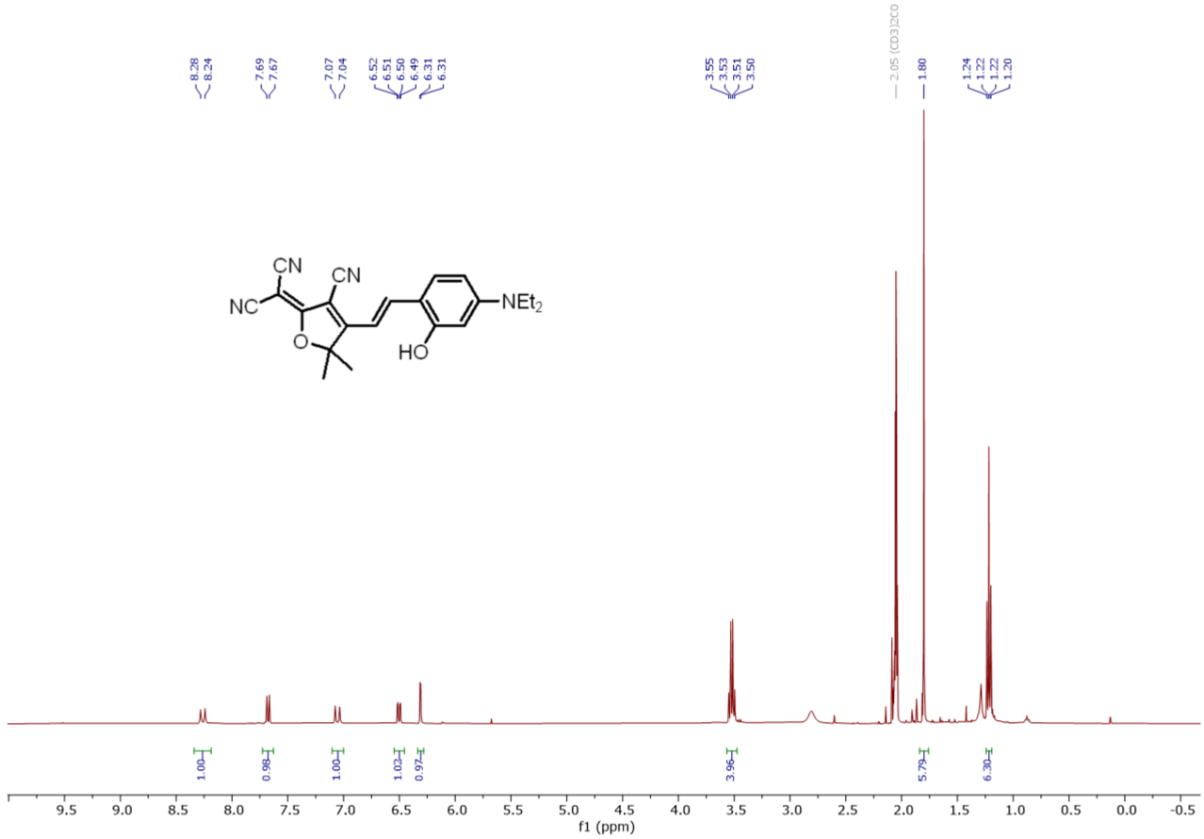

^{13}C NMR spectrum (126 MHz, Acetone-d_6) of compound **6f**

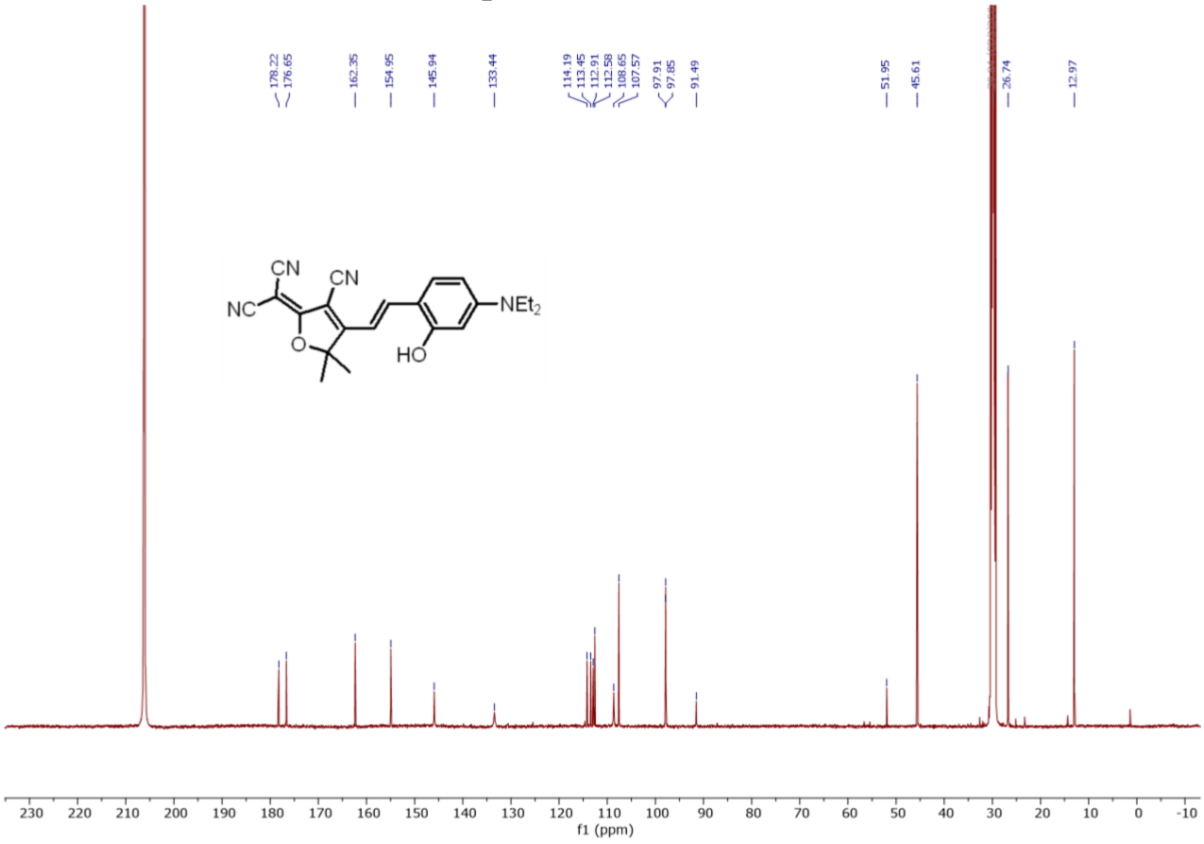

^{1}H NMR spectrum (400 MHz, $CDCl_3$) of compound **6g**

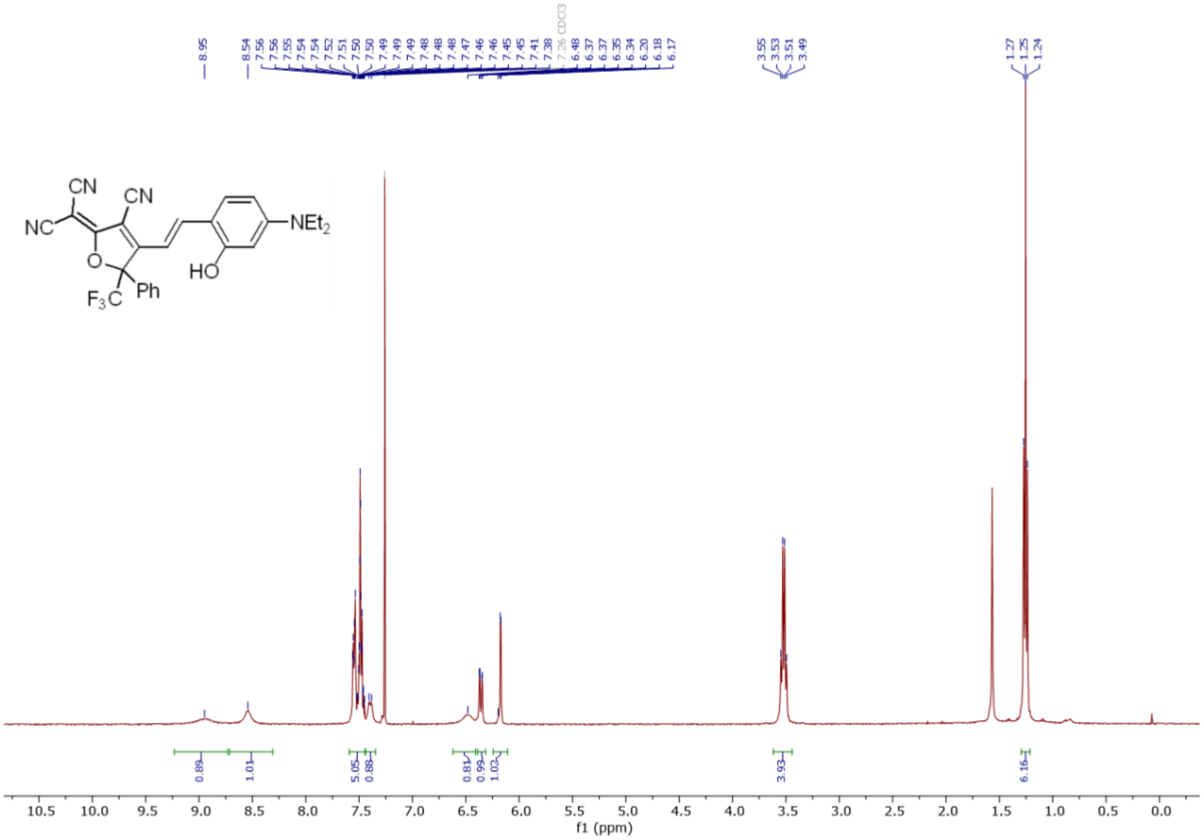

^{13}C NMR spectrum (126 MHz, $CDCl_3$) of compound **6g**

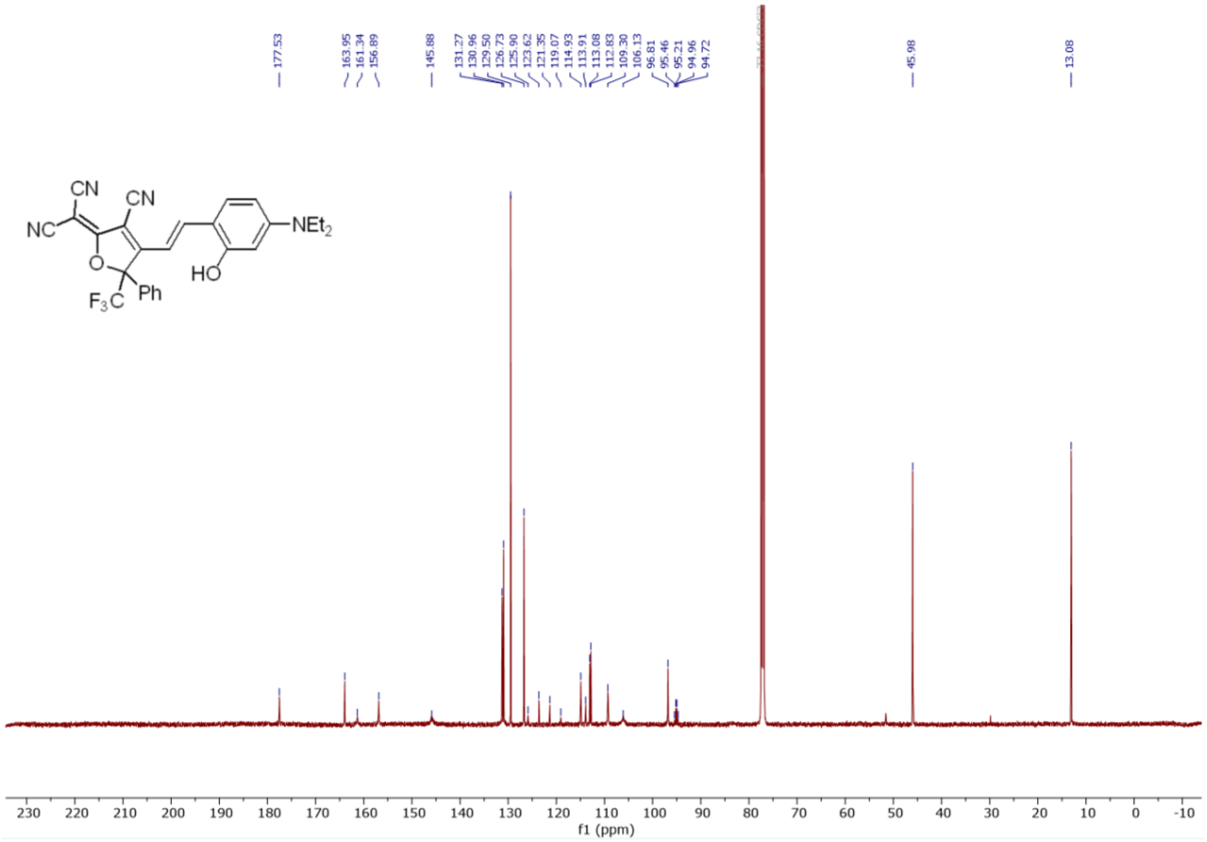

^{1}H NMR spectrum (400 MHz, $CDCl_3$) of compound **7**

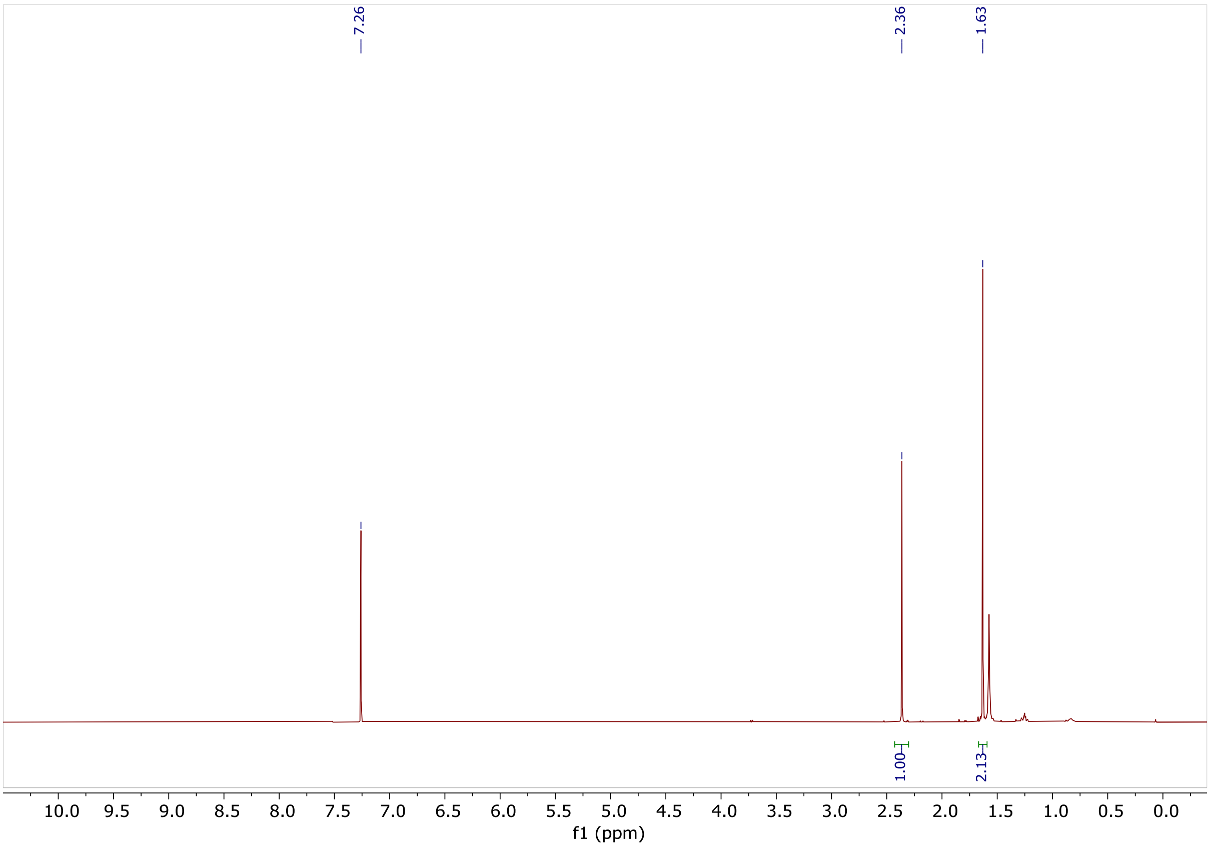

^{13}C NMR spectrum (101 MHz, DMSO-d_6) of compound **7**

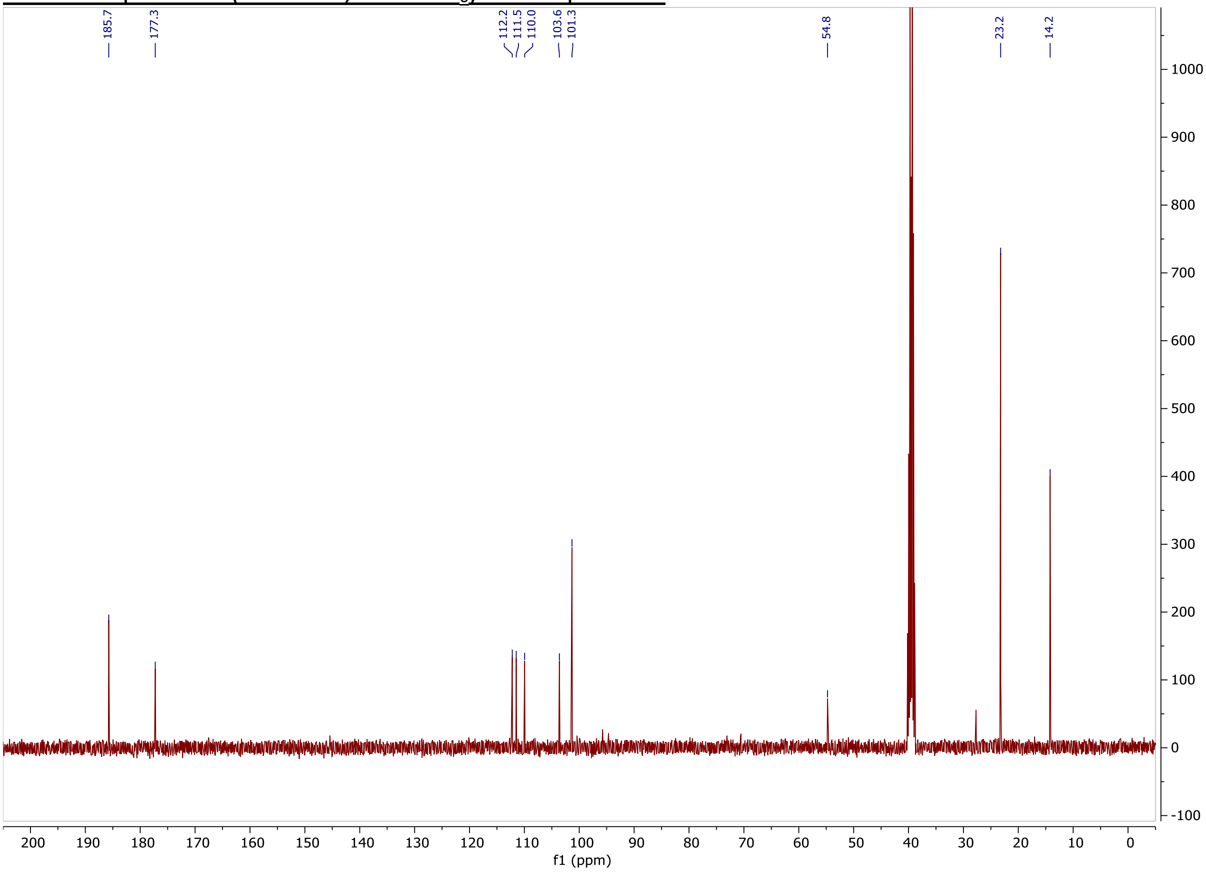

^{1}H NMR spectrum (400 MHz, DMSO-d_6) of compound **8**

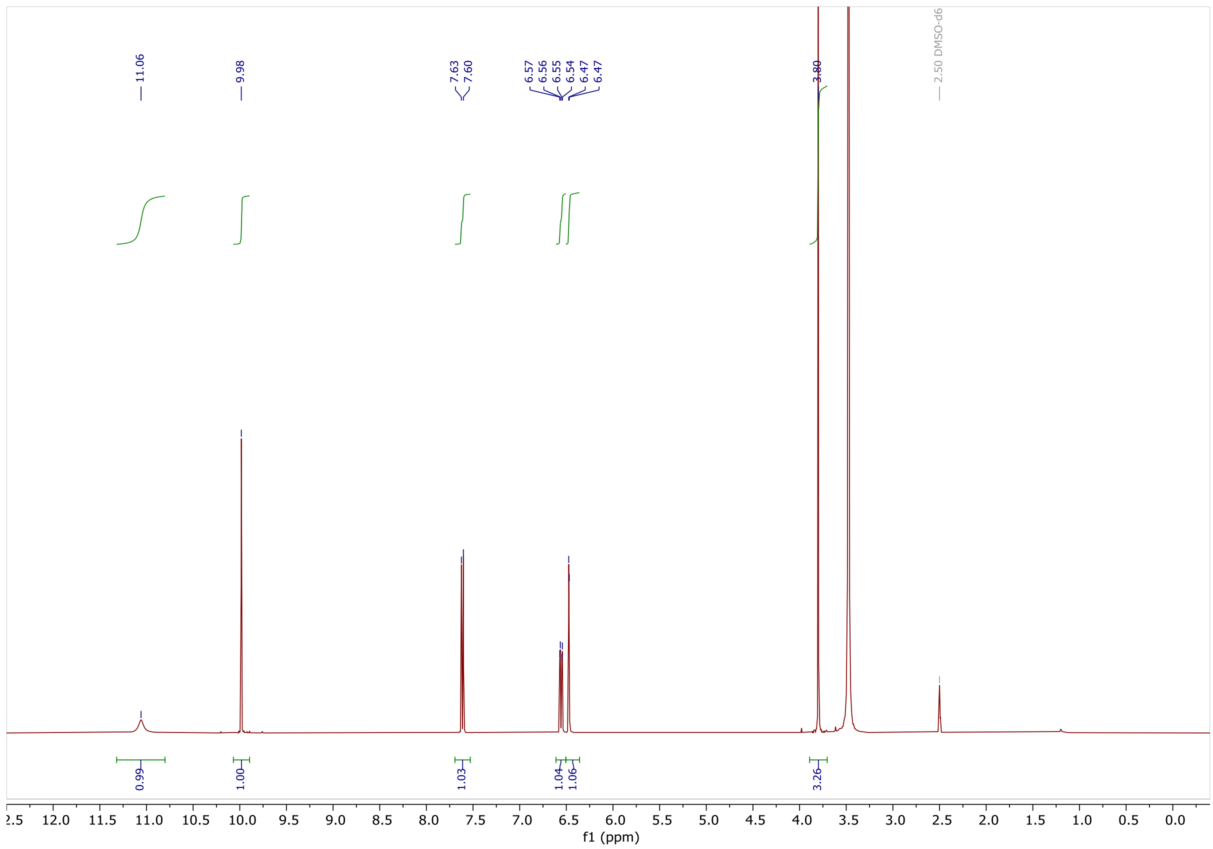

^{13}C NMR spectrum (101 MHz, DMSO-d_6) of compound **8**

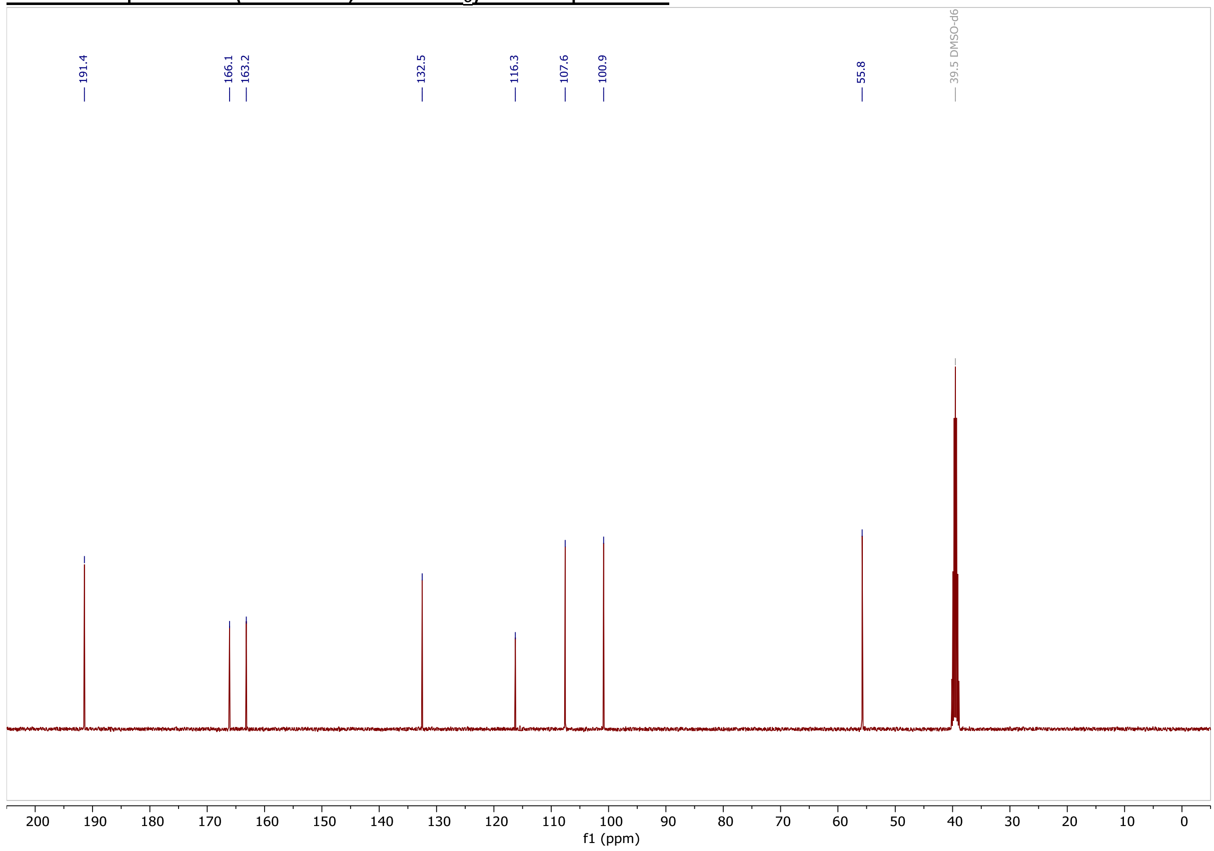

^{1}H NMR spectrum (400 MHz, $CDCl_3$) of compound **9**

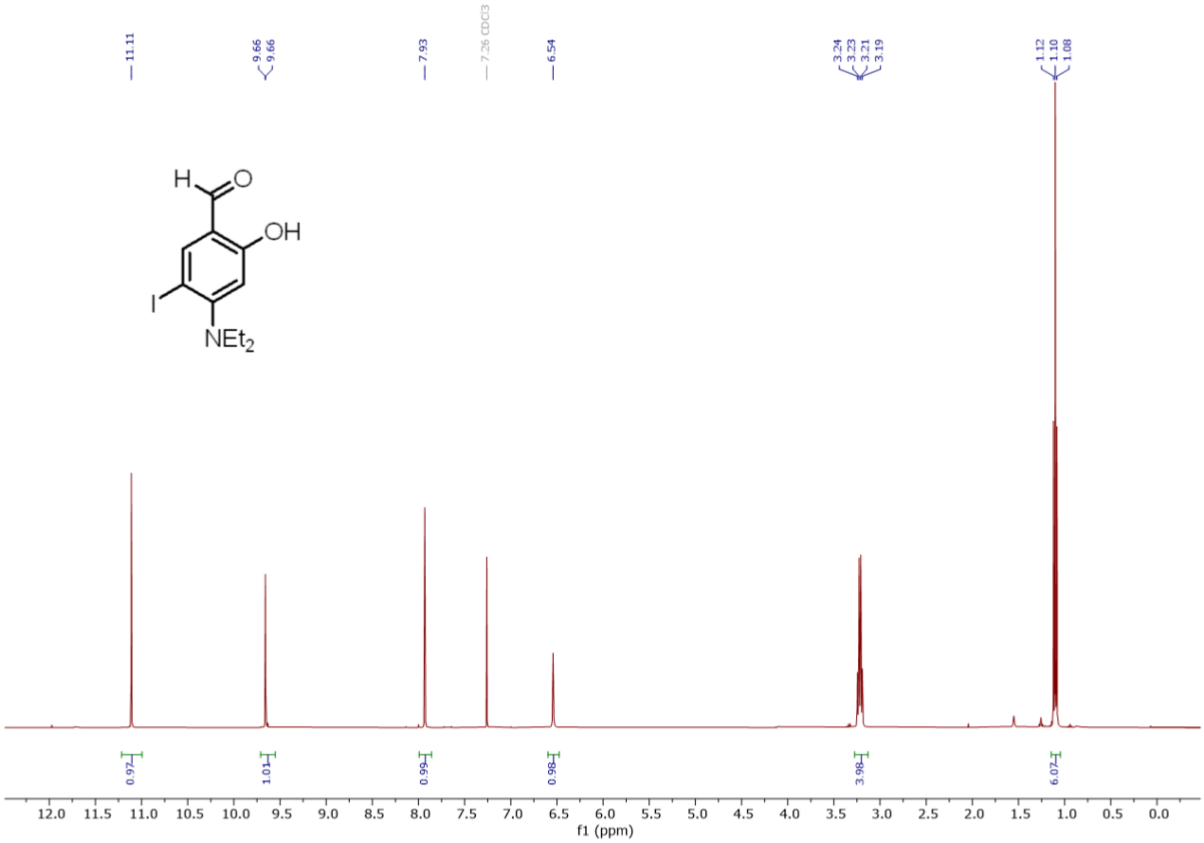

<u>^{13}C NMR spectrum (101 MHz, $CDCl_3$) of compound **9**</u>

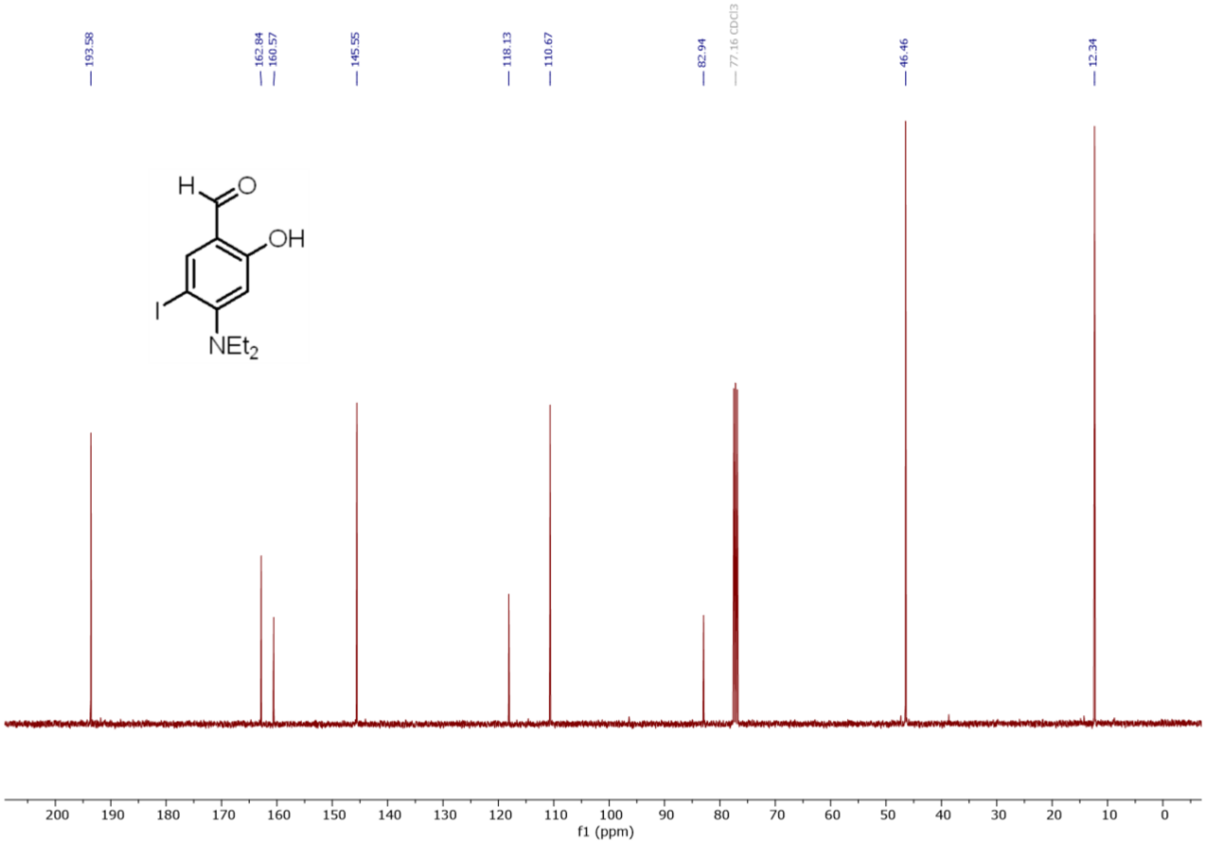

^{1}H NMR spectrum (400 MHz, $CDCl_3$) of compound **10**

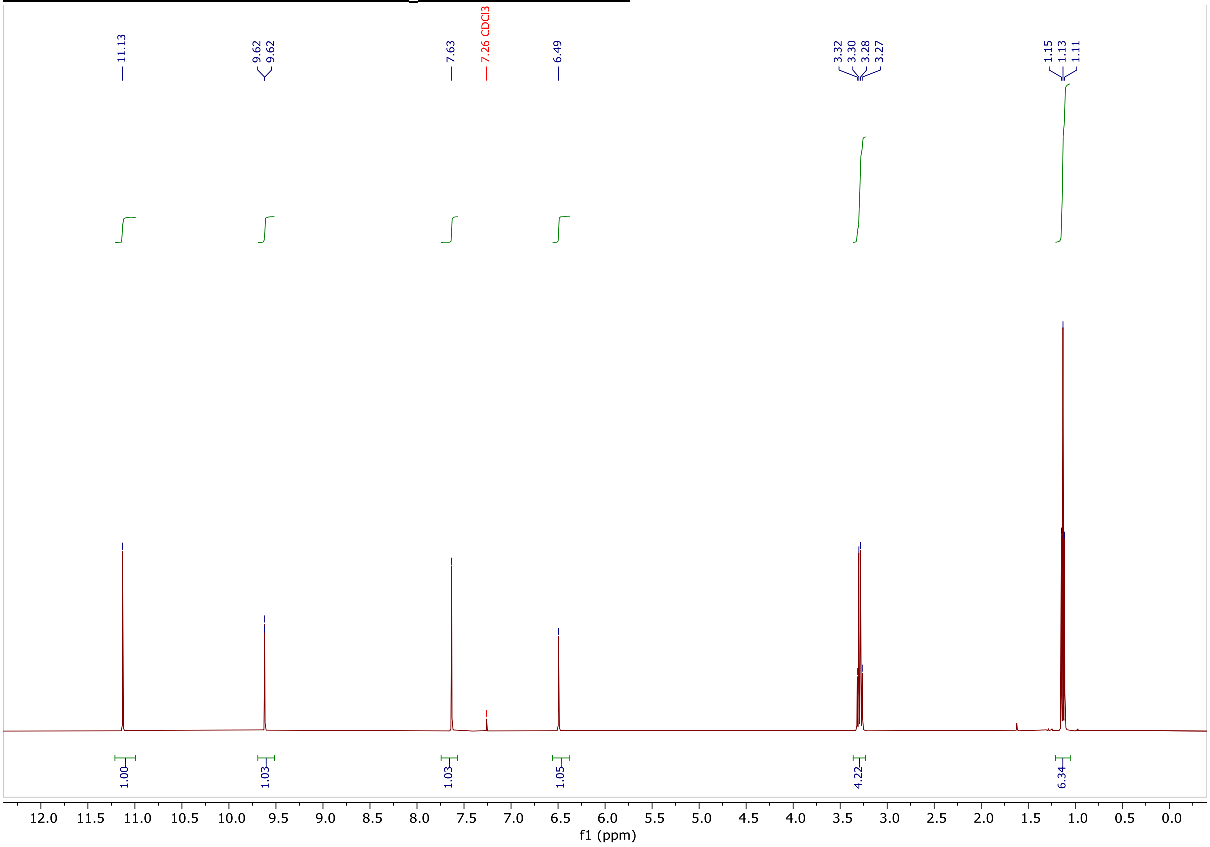

^{13}C NMR spectrum (101 MHz, $CDCl_3$) of compound **10**

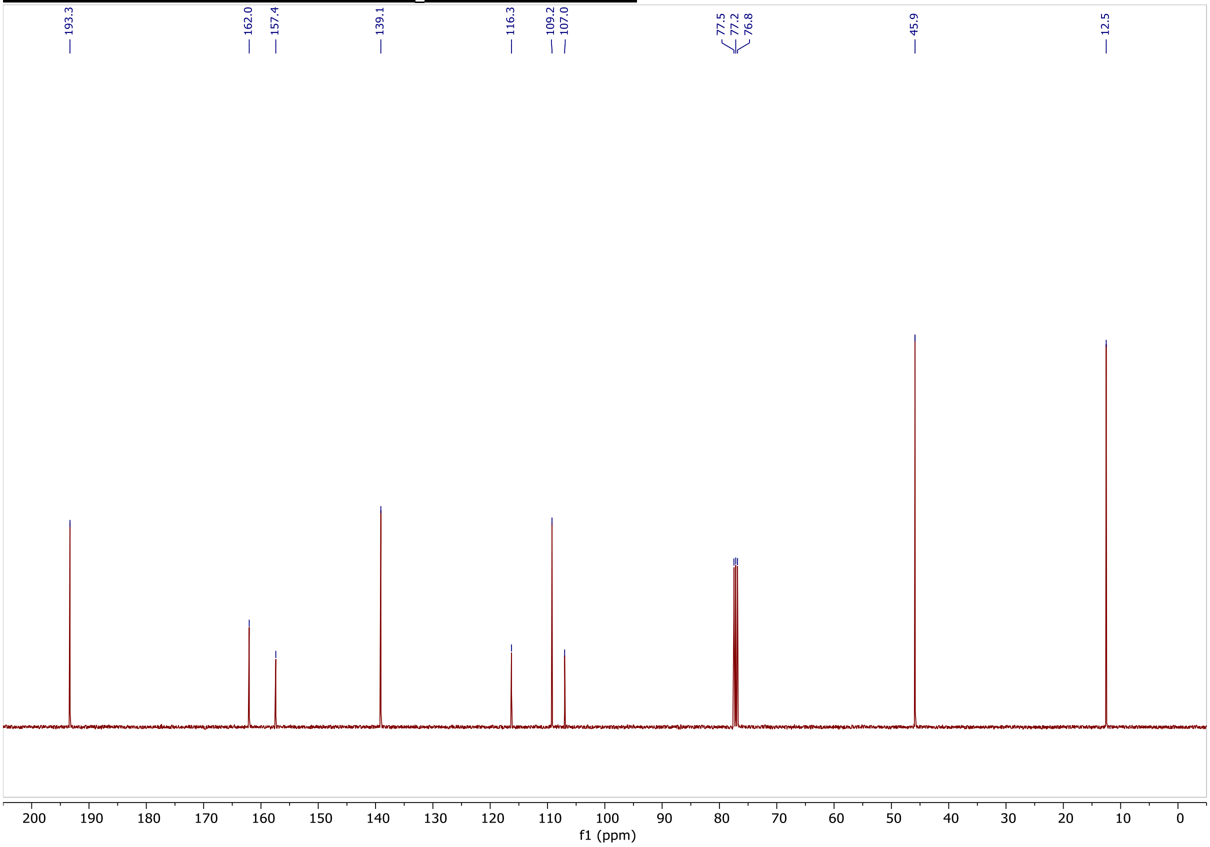

^{1}H NMR spectrum (400 MHz, $CDCl_3$) of compound **11**

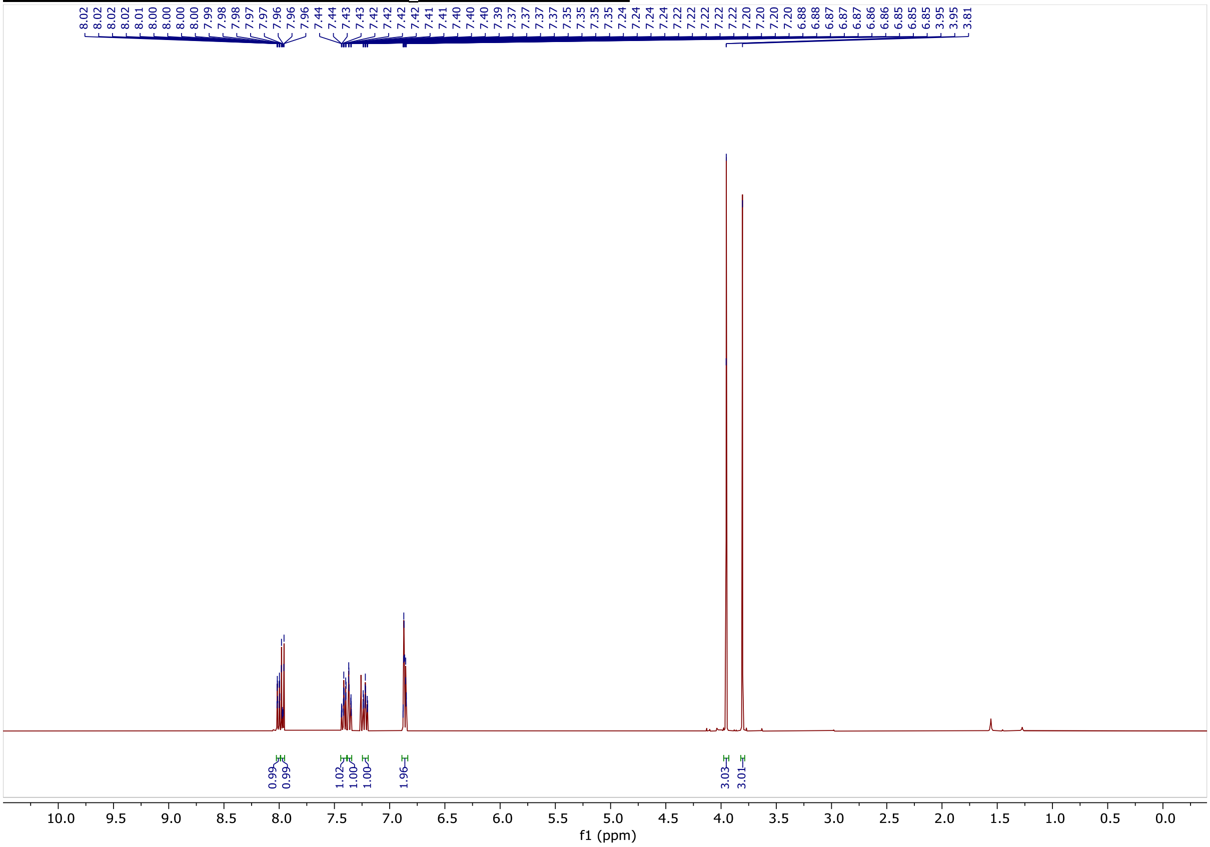

^{13}C NMR spectrum (101 MHz, $CDCl_3$) of compound **11**

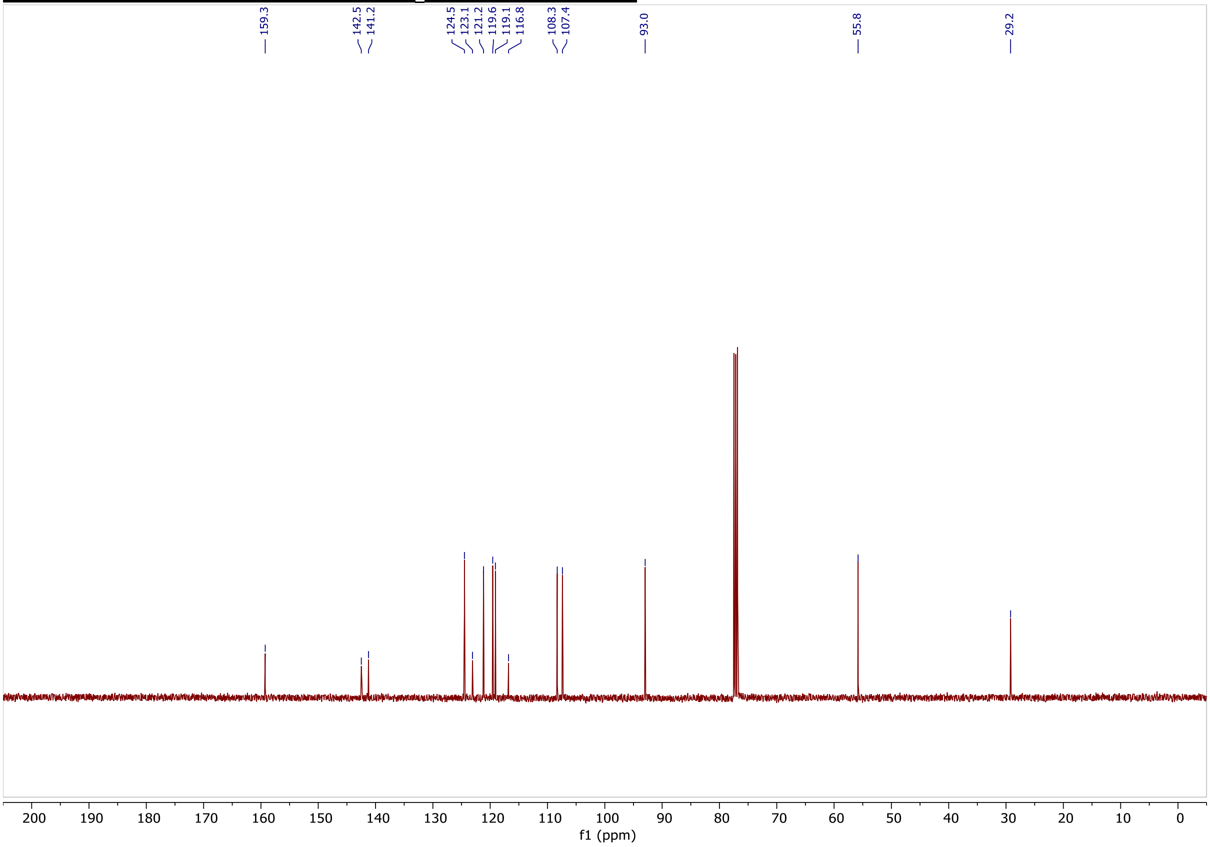

^{1}H NMR spectrum (400 MHz, $CDCl_3$) of compound **12**

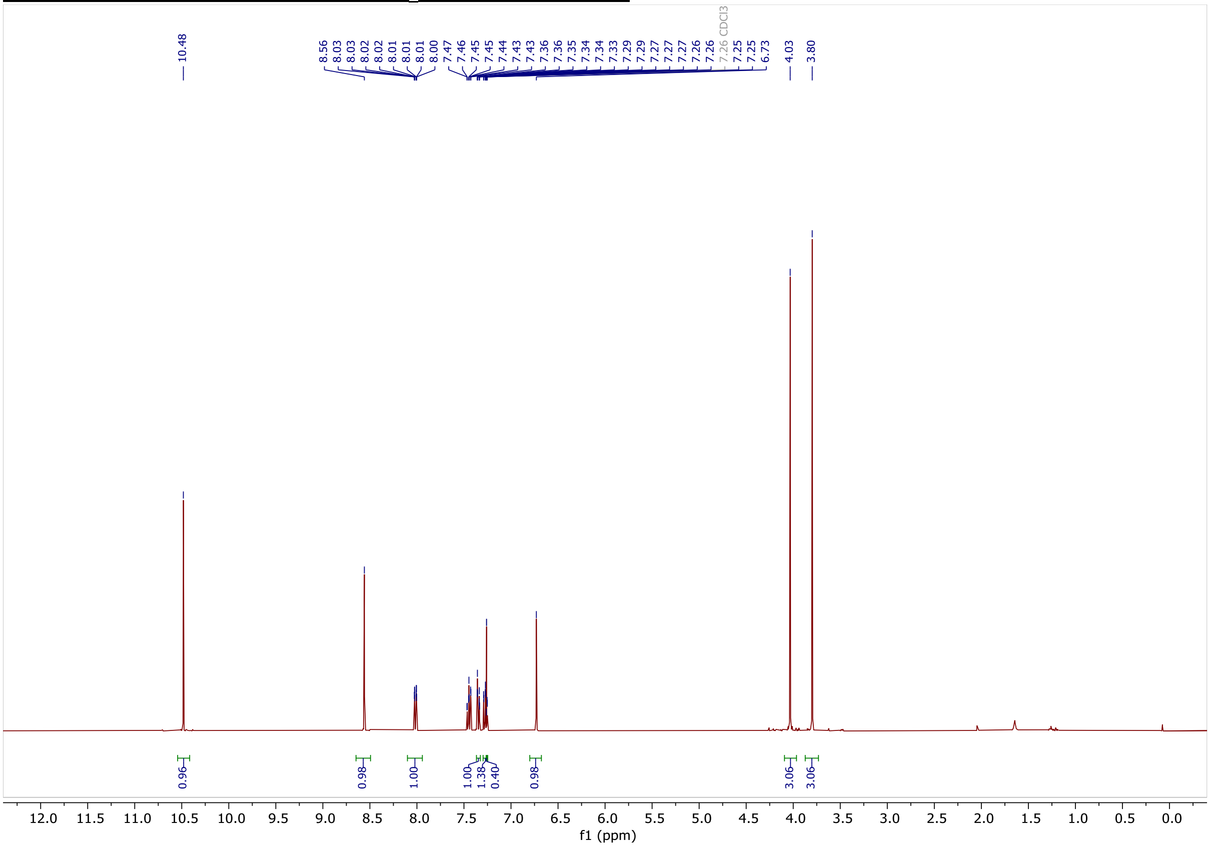

^{13}C NMR spectrum (101 MHz, $CDCl_3$) of compound **12**

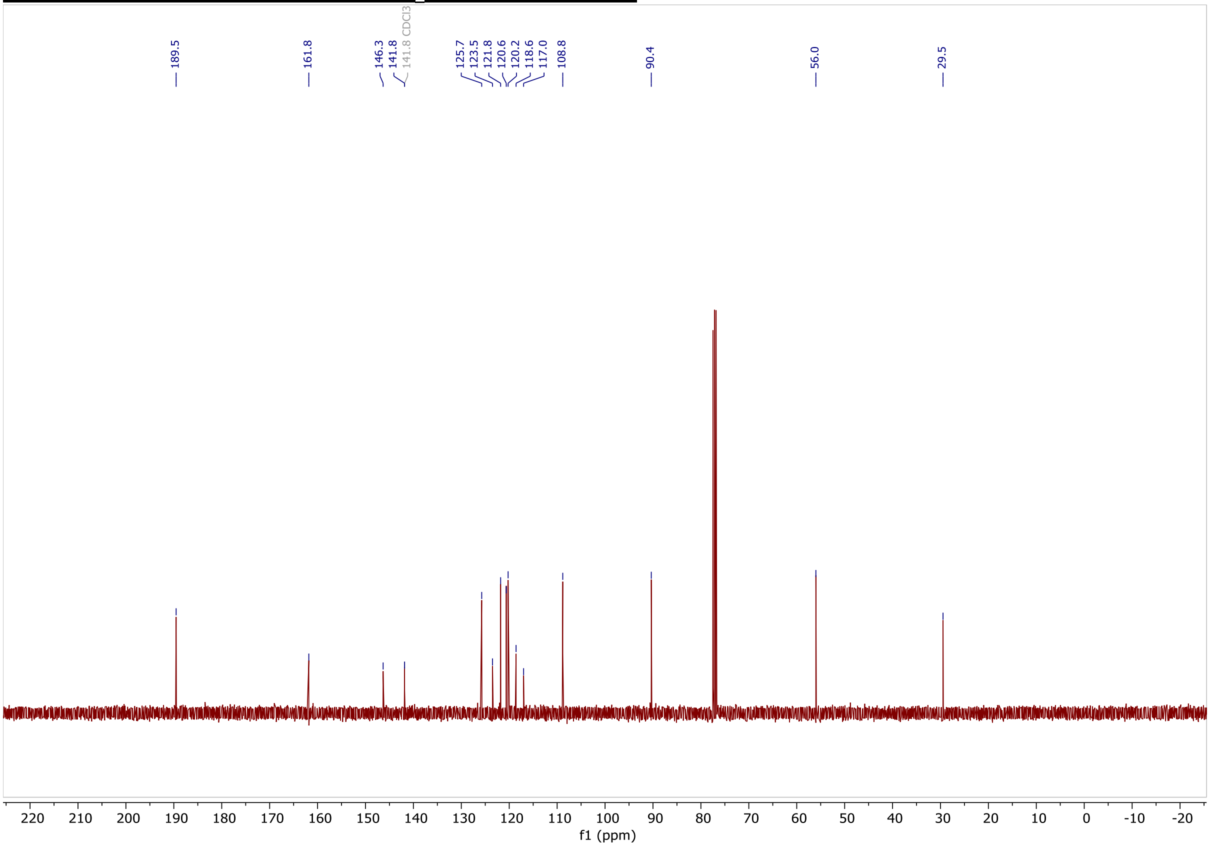

^{1}H NMR spectrum (400 MHz, $CDCl_3$) of compound **13**

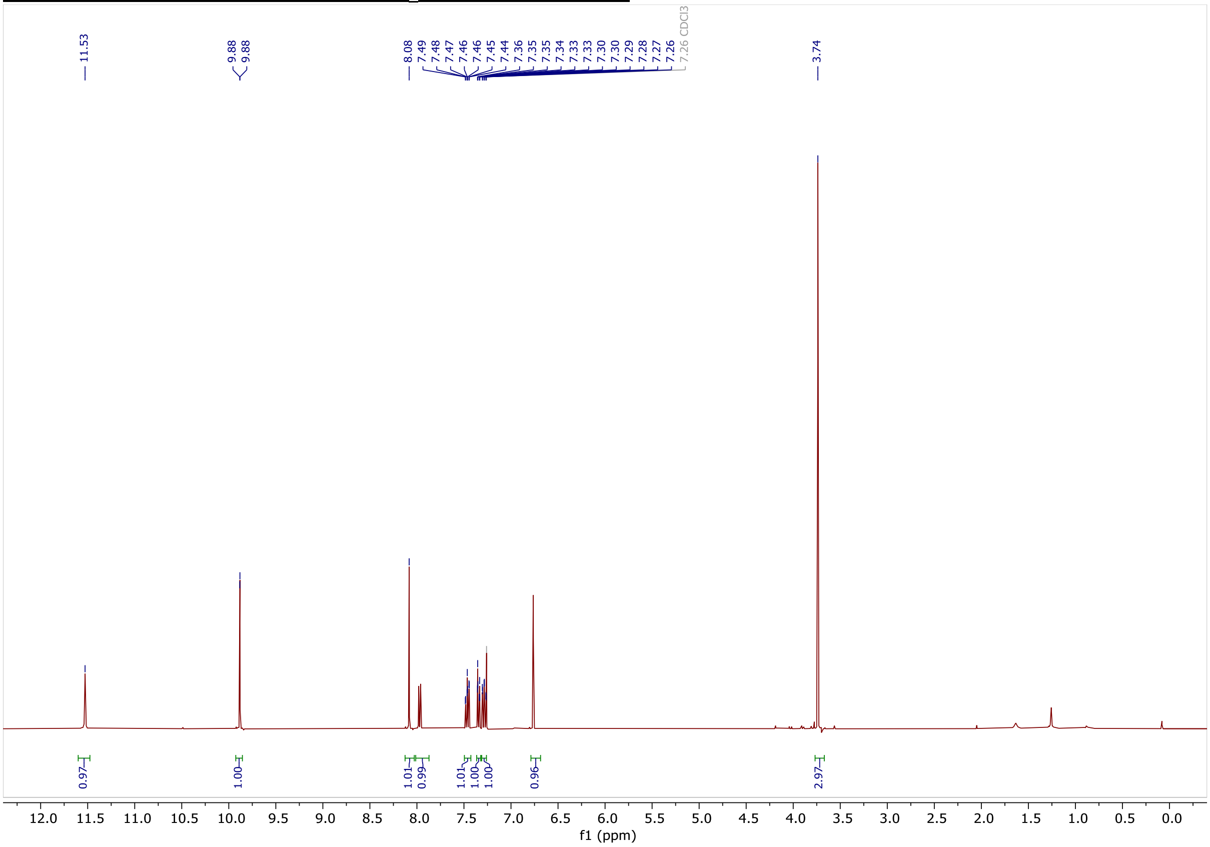

^{13}C NMR spectrum (101 MHz, $CDCl_3$) of compound **13**

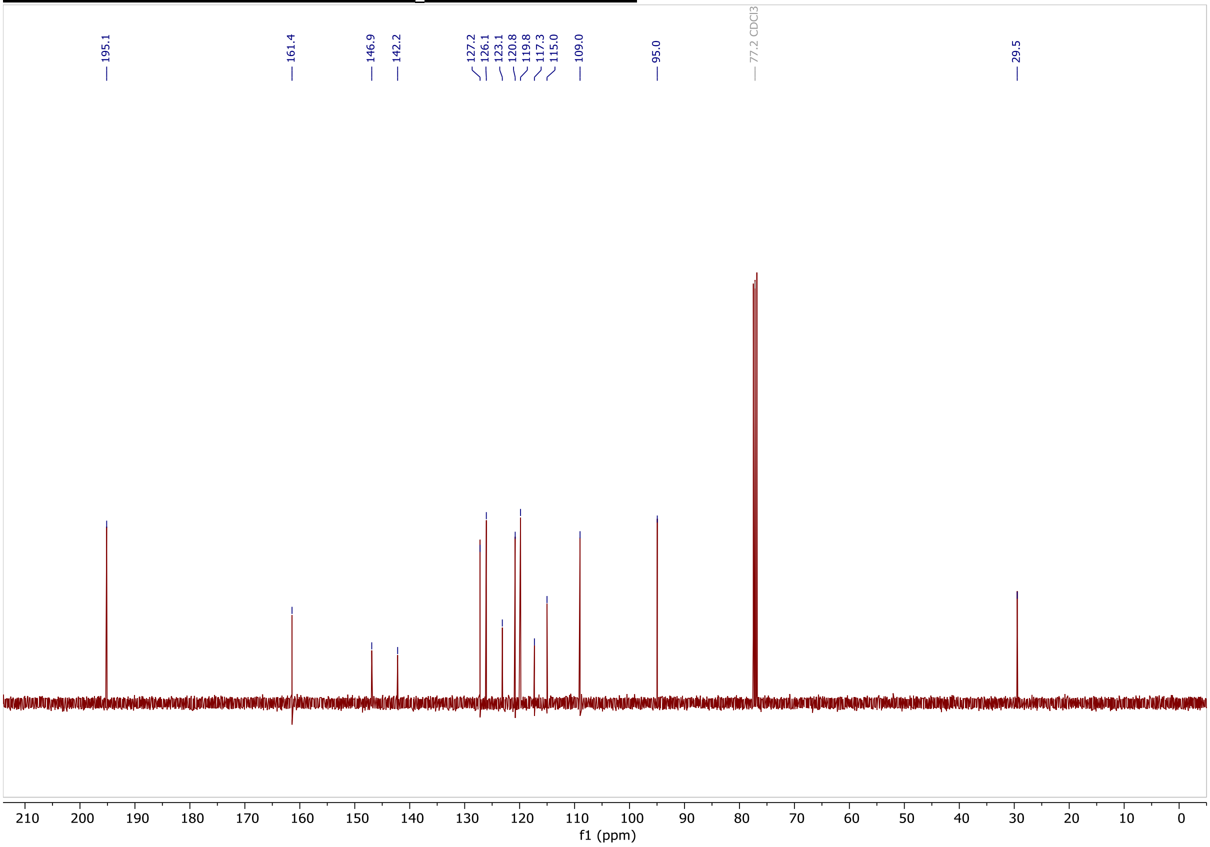

^{1}H NMR spectrum (400 MHz, $CDCl_3$) of compound **14**

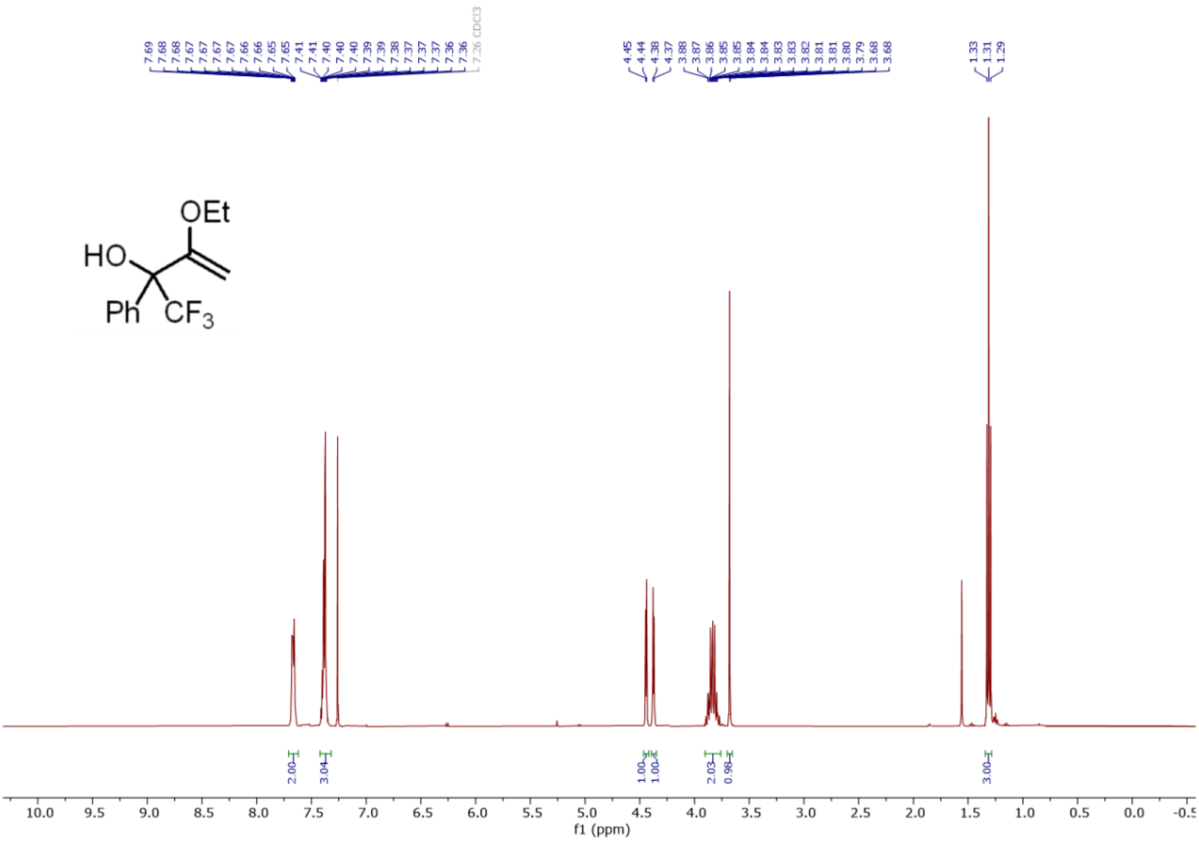

^{13}C NMR spectrum (101 MHz, $CDCl_3$) of compound **14**

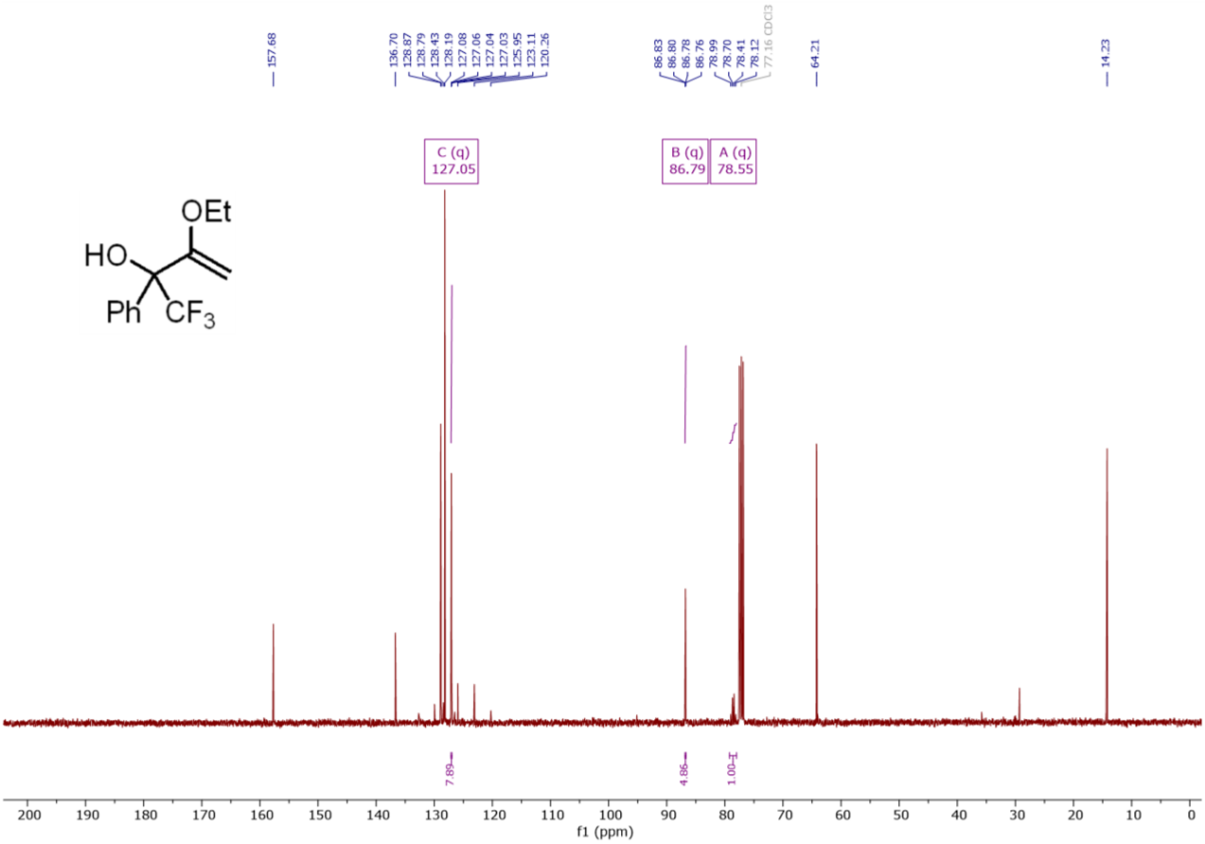

^1H NMR spectrum (400 MHz, $CDCl_3$) of compound **15**

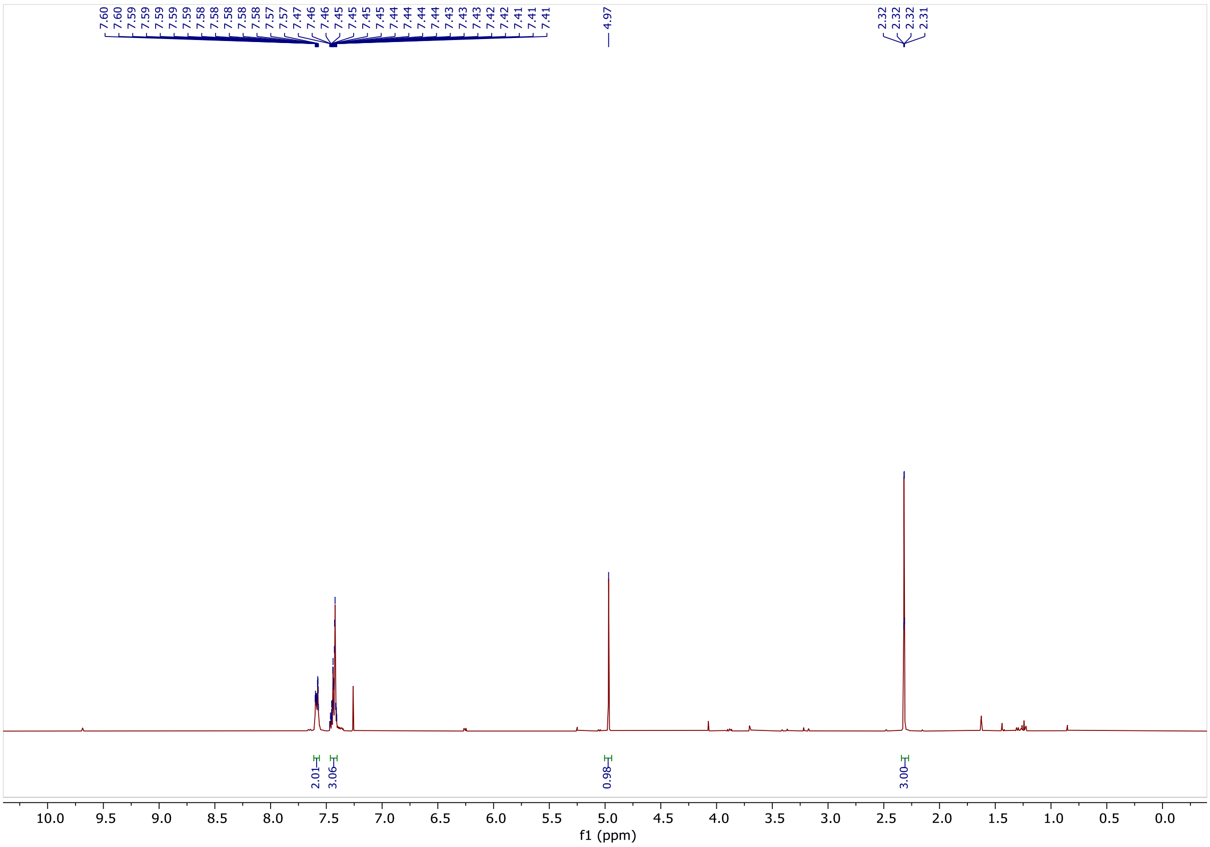

^{13}C NMR spectrum (101 MHz, $CDCl_3$) of compound **15**

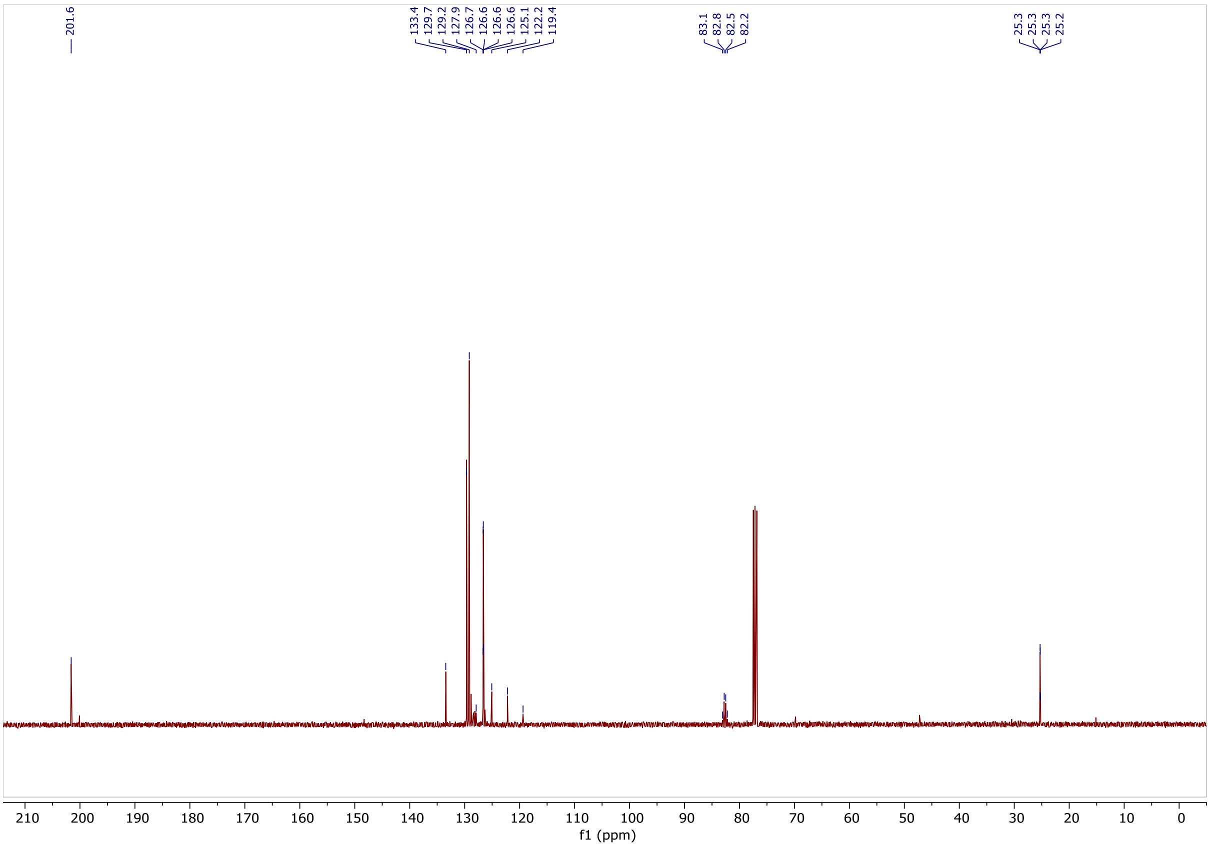

^{1}H NMR spectrum (400 MHz, $CDCl_3$) of compound **16**

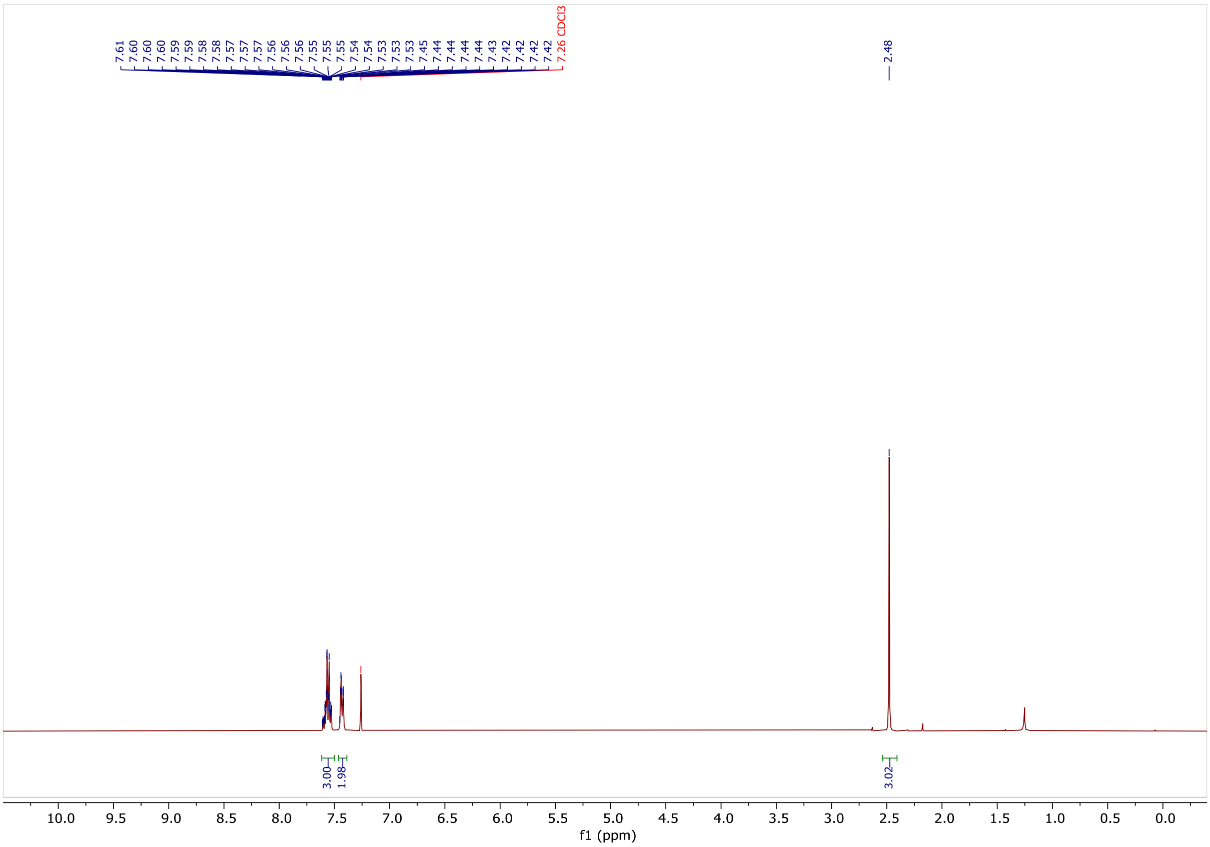

^{13}C NMR spectrum (101 MHz, $CDCl_3$) of compound **16**

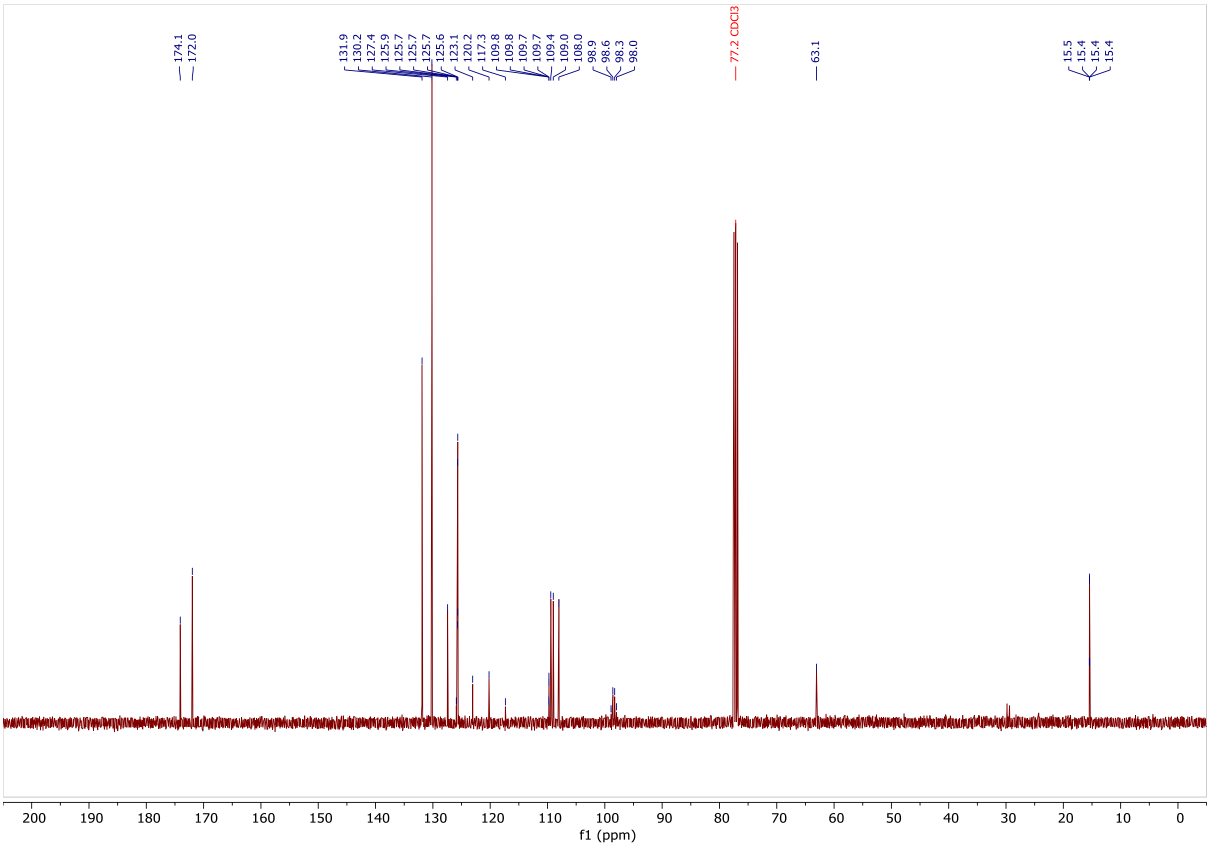

S7 References

1 M. M. Wootten, B. A. F. Le Bailly, S. Tshepelevitsh, I. Leito and J. Clayden, *Chem. Sci.*, 2022, **13**, 2258–2269.

2 D. Villemin and L. Liao, *Synth. Commun.*, 2001, **31**, 1771–1780.

3 J. P. Ferris, R. A. Sanchez and R. W. Mancuso, *Org. Synth.*, 1968, **48**, 1.

4 M. C. Davis, A. P. Chafin, R. A. Hollins, L. C. Baldwin, E. D. Erickson, P. Zarras and E. C. Drury, *Synth. Commun.*, 2004, **34**, 3419–3429.

5 C. Yang, T. Khalil and Y. Liao, *RSC Adv.*, 2016, **6**, 85420–85426.

6 D. Sang, H. Yue, Z. Zhao, P. Yang and J. Tian, *J. Org. Chem.*, 2020, **85**, 6429–6440.

7 J. T. Hou, J. Yang, K. Li, K. K. Yu and X. Q. Yu, *Sens. Actuators, B Chem.*, 2015, **214**, 92–100.

8 R. J. Tang, T. Milcent and B. Crousse, *J. Org. Chem.*, 2018, **83**, 930–938.

9 S. Song, X. Sun, X. Li, Y. Yuan and N. Jiao, *Org. Lett.*, 2015, **17**, 2886–2889.

10 J. E. T. Corrie, V. R. N. Munasinghe and W. Rettig, *J. Heterocycl. Chem.*, 2000, **37**, 1447–1455.

11 Wang, L. Zhao, S. Bi, Y. Gao, D. Wang, H. Chen, Y. Liu, C. Zhang, W. Zhang, X. Guo and Y. Nan, WO2014194826, 2014.

12 G. Bubniene, T. Malinauskas, M. Daskeviciene, V. Jankauskas and V. Getautis, *Tetrahedron*, 2010, **66**, 3199–3206.

13 H. Chen and Y. Liao, *J. Photochem. Photobiol. A*, 2015, **300**, 22–26.

14 S. Ramesh and R. Nagarajan, *J. Chem. Sci.*, 2014, **126**, 1049–1054.

15 G. J. Gainsford, M. Ashraf and A. J. Kay, *Acta Crystallogr. Sect. E Struct. Rep. Online*, 2012, **68**, o2991–o2992.

16 M. He, T. M. Leslie and J. A. Sinicropi, *Chem. Mater.*, 2002, **14**, 2393–2400.

17 H. Deng, Y. Fang, M. He, H. Hu, W. Niu and H. Sun, WO2012012278A2, 2013.

18 BindFit, http://app.supramolecular.org/bindfit/ (accessed 13 March 2023).

19 A. Klamt, *J. Phys. Chem.*, 1995, **99**, 2224–2235.

20 A. Klamt, V. Jonas, T. Bürger and J. C. W. Lohrenz, *J. Phys. Chem. A*, 1998, **102**, 5074–5085.

21 F. Eckert and A. Klamt, *AIChE J.*, 2002, **48**, 369–385.

22 TURBOMOLE V7.7 2022, a development of University of Karlsruhe and Forschungszentrum Karlsruhe GmbH, 1989-2007, TURBOMOLE GmbH, since 2007; available from https://www.turbomole.org.

23 BIOVIA COSMOtherm, Release 2023; Dassault Systèmes. http://www.3ds.com

24 A. Kütt, S. Tshepelevitsh, J. Saame, M. Lõkov, I. Kaljurand, S. Selberg and I. Leito, *European J. Org. Chem.*, 2021, **2021**, 1407–1419.

25 K. Kaupmees, I. Kaljurand and I. Leito, *J. Phys. Chem. A*, 2010, **114**, 11788–11793.
